# Triamine and Tetramine Edge-Length Matching Drives Heteroleptic Triangular and Tetragonal Prism Assembly

**DOI:** 10.1021/jacs.3c11320

**Published:** 2024-02-13

**Authors:** Jack A. Davies, Tanya K. Ronson, Jonathan R. Nitschke

**Affiliations:** Yusuf Hamied Department of Chemistry, University of Cambridge, Lensfield Road, Cambridge CB2 1EW, United Kingdom

## Abstract

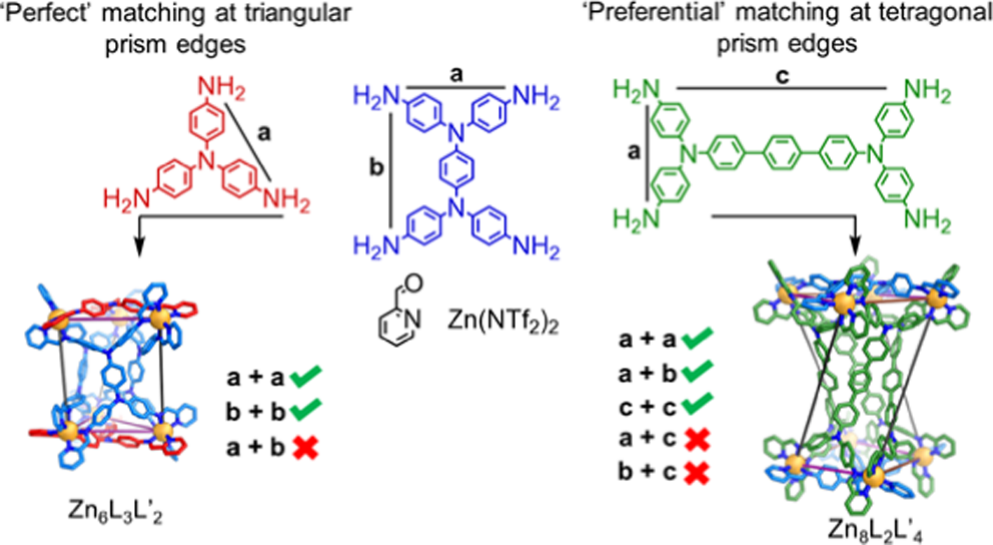

Heteroleptic metal–organic capsules, which incorporate more than one type of ligand, can provide enclosed, anisotropic interior cavities for binding low-symmetry molecules of biological and industrial importance. However, the selective self-assembly of a single mixed-ligand architecture, as opposed to the numerous other possible self-assembly outcomes, remains a challenge. Here, we develop a design strategy for the subcomponent self-assembly of heteroleptic metal–organic architectures with anisotropic internal void spaces. Zn_6_Tet_3_Tri_2_ triangular prismatic and Zn_8_Tet_2_Tet′_4_ tetragonal prismatic architectures were prepared through careful matching of the side lengths of the tritopic (Tri) or tetratopic (Tet, Tet′) and panels.

## Introduction

The self-assembly of more than one type of ligand into a single metal–organic architecture results in the generation of a heteroleptic assembly. If selective, this process provides a route to complex architectures without a need to build complexity into the ligands themselves.^1^ The inherently lower symmetries of heteroleptic architectures can lend anisotropy to their cavities, thus priming them to bind lower-symmetry guest molecules.^2^

In order to selectively prepare a single heteroleptic structure, the different ligands must be directed to assemble together integratively instead of undergoing narcissistic self-sorting, where homoleptic assemblies form together in parallel.^3^ Competing assembly pathways where mixtures of heteroleptic assemblies are formed,^4^ as opposed to a single one, must also be avoided.^5^

Stang,^6^ Schmittel,^7^ Fujita,^8^ and others^9^ have developed elegant approaches to drive the selective self-sorting of mixtures of subunits into single heteroleptic metal–organic assemblies. Approaches pioneered by Clever,^ ^1c,2c,4b,10^^ Wang,^11^ and others^12^ have leveraged a good geometric match between different ligand types. Zhang and co-workers have utilized both geometric matching between ligands and principles of charge separation^6c^ to generate heteroleptic architectures from paneling ligands.^13^

We have recently reported triangular prismatic structures, assembled from the combination of tri- and tetratopic ligands.^ ^2a,14^^ The ligand panels provide enclosed internal volumes that enable guest binding. These heteroleptic structures were found to have a favorable entropy of formation relative to the corresponding homoleptic species.^2a^ We infer this favorable entropy, arising from the increased conformational flexibility of the triangular prism ligand panels and the encapsulation of fewer solvent molecules in the smaller cavity of the heteroleptic structure, to compensate for an enthalpic penalty. This unfavorable enthalpy change may be associated with the joining of subcomponent sides having different lengths at the edges making up the triangular faces of the triangular prism, compared with the matching of identical subcomponent sides at all edges in the homoleptic tetrahedron and cube. When the tetratopic ligands corresponded to rectangular as opposed to square panels, heteroleptic cages formed in which subcomponents adopt multiple different configurations within a system of interconverting diastereomeric structures.^14^

This work establishes a general geometric design method for the subcomponent self-assembly of heteroleptic triangular prisms (as single diastereomers) and a tetragonal prismatic structure type. The subcomponent self-assembly of rectangular tetra-anilines with a threefold-symmetric trianiline, zinc(II) *bis*(trifluoromethanesulfonyl)imide (triflimide, ^–^NTf_2_) and 2-formylpyridine in acetonitrile yielded Zn_6_L_3_L′_2_ triangular prismatic assemblies. The selective formation of a single product in each case was driven by matching the separations between adjacent aniline groups of the trianiline with one of the rectangular axes of the tetra-aniline. Pairing a low-aspect-ratio rectangular subcomponent with a more elongated rectangular subcomponent similarly resulted in the formation of a Zn_8_L_2_L′_4_ tetragonal prism. Utilizing the design principles deciphered from these systems, a heteroleptic architecture was then conceived and assembled from two distinct classes of tetra-aniline subcomponent.

## Results and Discussion

**Figure 1:**
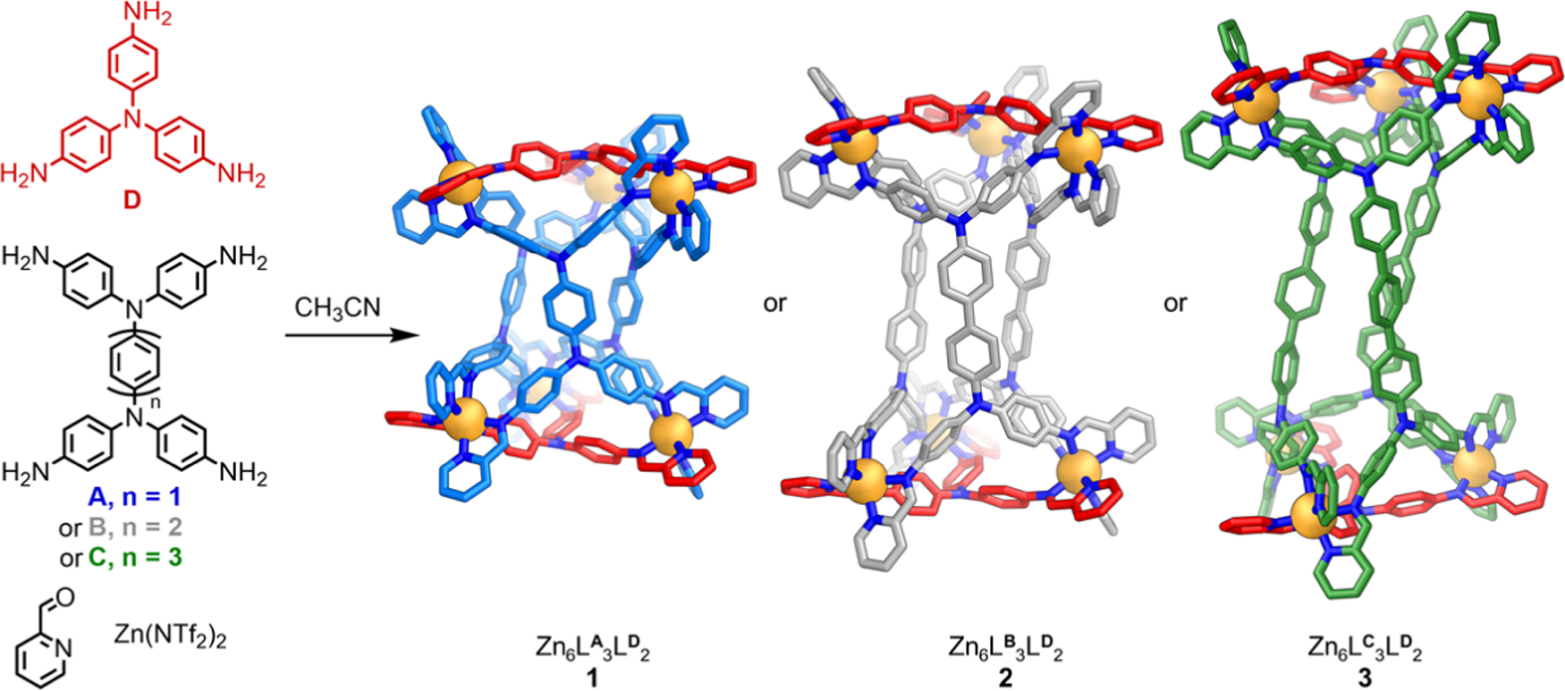
Subcomponent self-assembly of Zn_6_L_3_L′_2_ distorted triangular prisms **1**–**3**. Products **1**–**3** are displayed as the crystal structures, with solvent, including acetonitrile molecules residing within the interior cavity of each structure, counterions, disorder, and hydrogen atoms omitted for clarity. Zn^II^: orange, N: blue, C: red, light blue, gray or green, depending on the multitopic aniline residue.

Aniline subcomponents **A**–**E** were either purchased from commercial suppliers or synthesized as described in Supporting Information Section 2. The reaction between tetra-aniline **A**, trianiline **D**, Zn(NTf_2_)_2_, and 2-formylpyridine in acetonitrile yielded metal–organic architecture **1** (Figure 1). As detailed in Supporting Information Section 3.1, maximization of the yield of **1** required an excess of tetra-aniline **A**, Zn(NTf_2_)_2_ and 2-formylpyridine, which we inferred to be due to these subcomponents forming insoluble side products, as well as forming **1** in combination with **D**, under the conditions used. A digestion experiment, in which the insoluble material and metal–organic cage **1** were separately dissolved in acidic DMSO-*d*_6_, supported this inference. ^1^H NMR spectroscopy (Figure S15) indicated the presence of tetra-aniline **A** in the digested insoluble side product, whereas both **A** and **D** were observed in the ^1^H NMR spectrum of digested prism **1**.

Crystals were obtained as detailed in Supporting Information, Section 4. The solid-state structure of **1** was elucidated by single-crystal X-ray diffraction (XRD) using synchrotron radiation.^15^ The crystal structure revealed a [Zn_6_L^**A**^_3_L^**D**^_2_]^12+^ assembly, where L^**A**^ and L^**D**^ are the *tetrakis*(bidentate) and *tris*(bidentate) ligands formed from the condensation of the corresponding multitopic aniline with 2-formylpyridine.^16^ The six Zn^II^ centers reside at the corners of a distorted triangular prism, with the tritopic and tetratopic ligands paneling triangular and quadrilateral faces, respectively. All six Zn^II^ centers within **1** have the same handedness, Λ in Figure 1, with both enantiomers of **1** related by inversion present within the crystal.

**Figure 2:**
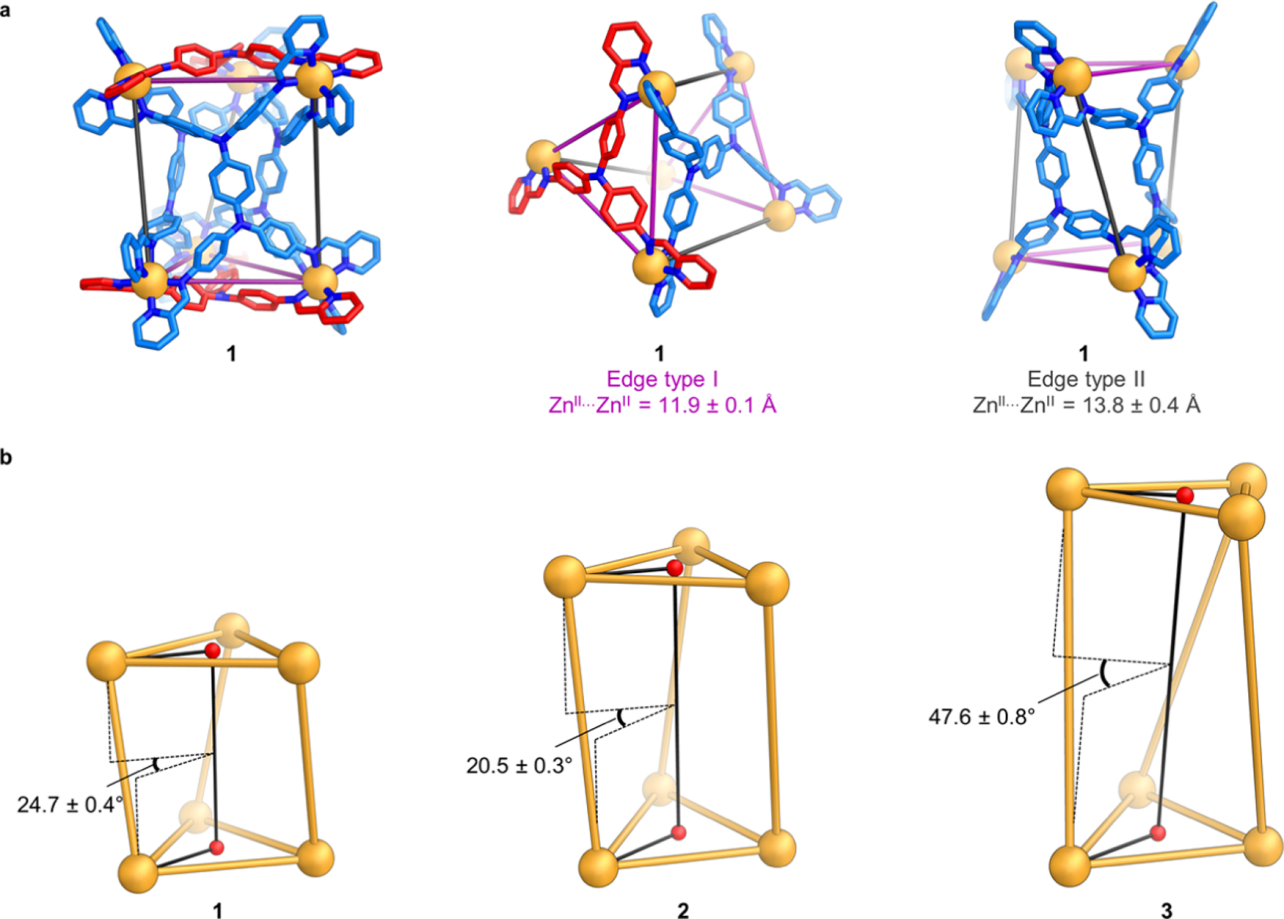
(a) Partial views of the crystal structure of **1**, showing the two edge types in magenta and gray. Zn^II^: orange, N: blue, C: red or light blue, depending on the multitopic aniline residue. (b) Twists in triangular prisms **1**–**3**, described by the Zn^II^ (top face)···centroid···(top face)···centroid (bottom face)···Zn^II^ (bottom face) dihedral angle. The selection of Zn^II^ (top face) and Zn^II^ (bottom face) for calculating the dihedral angle is such that they form a triangular prism edge where the long axes of two tetra-aniline residues meet. The mean angle for each structure was calculated from the three values of this dihedral angle measured from the corresponding crystal structures.

The three rectangular ligand panels within **1** adopt a single orientational configuration in the crystal. At the edges that make up the two triangular faces, the short rectangular axis of tetra-aniline **A** meets trianiline **D**, labeled as edge type I in Figure 2a. The mean Zn^II^···Zn^II^ distance for edge type I is 11.9 ± 0.1 Å. At the remaining three edges, labeled edge type II in Figure 2a, the long axes of two tetra-aniline **A** residues meet, with a longer Zn^II^···Zn^II^ distance of 13.8 ± 0.4 Å.

The electrospray ionization (ESI) mass spectrum of **1** was consistent with a [Zn_6_L^**A**^_3_L^**D**^_2_]^12+^ composition (Figures S12 and S13). The ^1^H NMR spectrum of **1** indicated the presence of three magnetically distinct ligand arms, consistent with a single Zn_6_L^**A**^_3_L^**D**^_2_ diastereomer with idealized *D*_3_ point symmetry (Figure S4), matching the solid-state structure.

Triangular prisms **2** and **3** were prepared by mixing trianiline **D**, 2-formylpyridine, and Zn(NTf_2_)_2_ in acetonitrile with **B** or **C**, respectively (Supporting Information Sections 3.2 and 3.3). Signals matching those expected for assemblies with the formulas [Zn_6_L^**B**^_3_L^**D**^_2_]^12+^ and [Zn_6_L^**C**^_3_L^**D**^_2_]^12+^ were identified in the ESI mass spectrum in each case (Figures S25, S26, S37 and S38). The ^1^H NMR spectra of **2** (Figure S17) and **3** (Figure S28) indicated the presence of three magnetically distinct ligand arms, consistent with the formation of a triangular prism with an idealized *D*_3_ symmetry in each case.

The crystal structures of **2** and **3** revealed twisted triangular prismatic structures analogous to **1** (Figure 1).^16^ The mean Zn^II^···Zn^II^ distances for edges at which the short axis of the tetra-aniline meets a trianiline in **2** and **3**—11.9 ± 0.1 Å in both **2** and **3**—match the value for the analogous edge type in **1** (edge type I in Figure 2a). As anticipated, the mean Zn^II^···Zn^II^ distance along the edges where the long axes of two tetra-aniline residues meet is longer in **2** (18.3 ± 0.1 Å) and **3** (22.5 ± 0.1 Å) than in **1** (13.8 ± 0.4 Å). Each of the assemblies **1**–**3** is twisted in the solid state (Figure 1). The twists were calculated to be 24.7 ± 0.4, 20.5 ± 0.3, and 47.6 ± 0.8° for **1**, **2**, and **3**, respectively (Figure 2b). The chirotopic cavities of the all-Δ and all-Λ enantiomers have helical twists of opposite-handedness.

**Figure 3:**
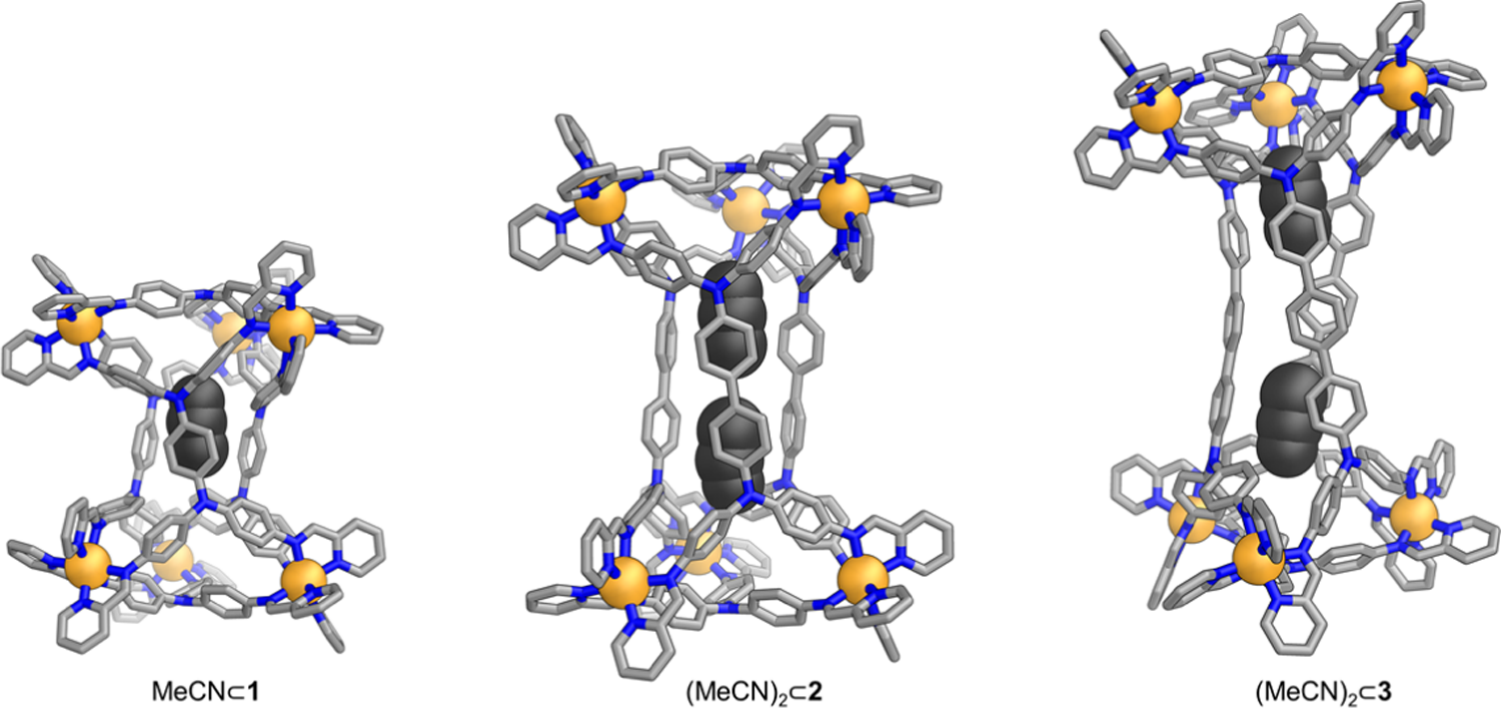
Views of the crystal structures of twisted triangular prisms Zn_6_L^**A**^_3_L^**D**^_2_ (**1**), Zn_6_L^**B**^_3_L^**D**^_2_ (**2**), and Zn_6_L^**C**^_3_L^**D**^_2_ (**3**). Solvent, counterions, disorder, and hydrogen atoms are omitted for clarity, except the acetonitrile molecule(s) residing in the cavity of each architecture. Zn^II^: orange, N: blue, C: light gray. All atoms in the acetonitrile guest molecules are colored dark gray and shown in space-filling mode.

Each of triangular prisms **1**–**3** provides a narrow, prolate internal cavity (Figure S73), which contrasts with the pseudospherical cavities of the Zn_8_L_6_ pseudocubes formed by tetra-anilines **A** and **B**.^17,18^ These cavities are thus well suited to binding matching guest molecules. As shown in Figure 3, in the crystals, an acetonitrile molecule resides within the cavity of prism **1**, while two acetonitrile molecules occupy the cavities of **2** and **3**. The absence of end-on-end disorder of the acetonitrile molecules within the cavities of **2** and **3** implies that the nitrile groups are oriented selectively to face outward, with the methyl groups directed toward the center. We infer that this selectivity in acetonitrile guest orientation may arise from the preference for the δ^–^ region of its dipole to point toward the positively charged Zn^II^ centers at each end of the structure. Furthermore, we infer that the presence and position of acetonitrile guest molecules within the cavities of **1**–**3** may influence the degree of twist observed.

The selective formation of triangular prism **1** as a single diastereomer may thus be explained by the preference to match the long axes of tetra-aniline **A** residues, and the short **A** axis with trianiline **D**. By contrast, in the previously reported Zn_8_L^**A**^_6_ pseudocube, the two distinct rectangular axes of subcomponent **A** residues mismatch at edges formed by pairs of *fac* Zn^II^ centers with the same handedness.^17^ This ability to form polyhedron edges where the axes of subcomponent **A** residues mismatch inspired the design and construction of the heteroleptic tetragonal prism **4**.

**Figure 4:**
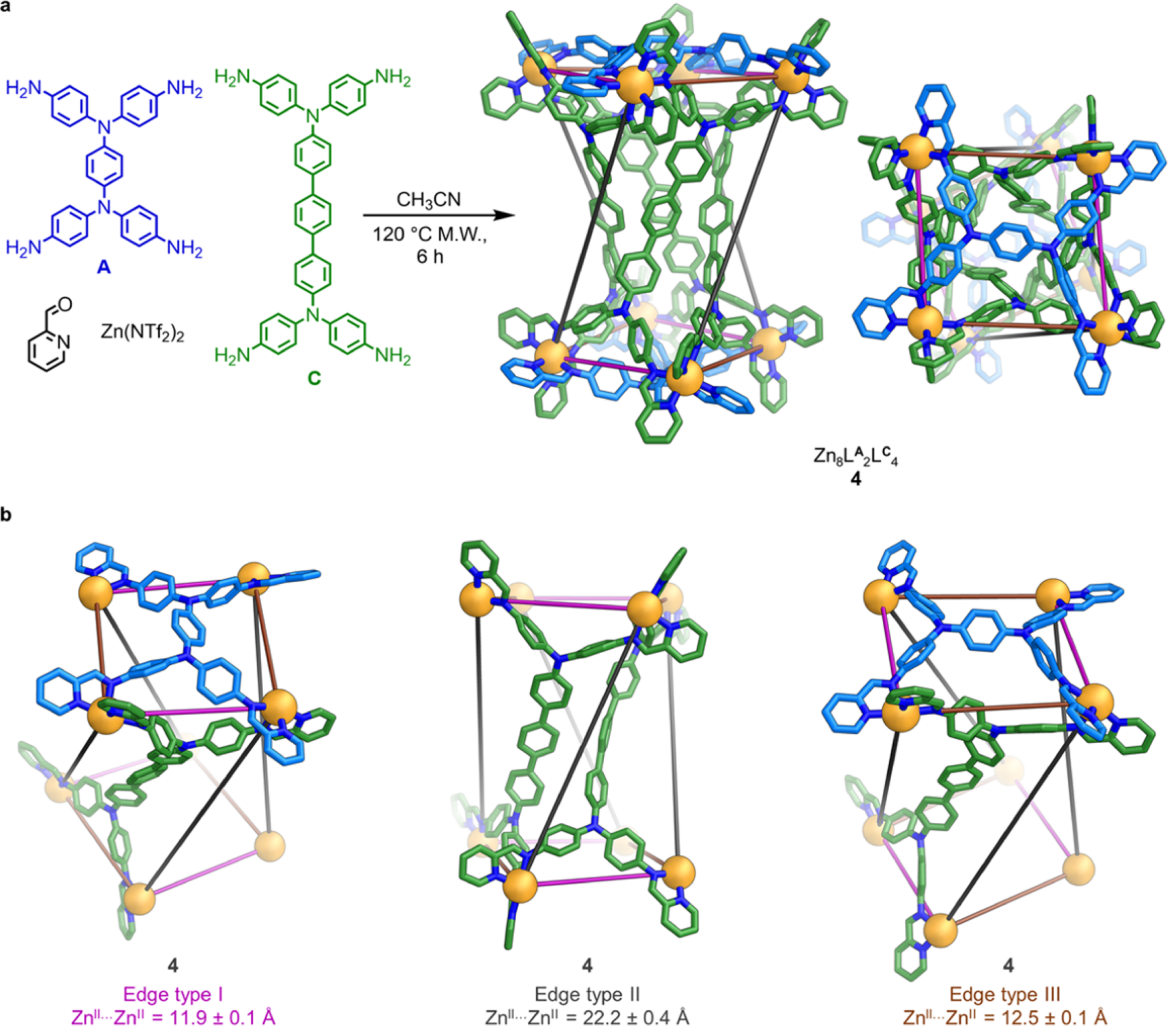
(a) Subcomponent self-assembly of Zn_8_L^**A**^_2_L^**C**^_4_ twisted tetragonal prism **4**, which is shown as the X-ray crystal structure. Solvent, counterions, disorder, and hydrogen atoms, including the diisopropyl ether and hexafluorophosphate residing inside the cavity, are omitted from the crystal structure for clarity. (b) Three distinct edge types in **4**, highlighted in partial views of the crystal structure. Zn^II^: orange, N: blue, C: light blue or green, depending on the multitopic aniline residue.

The reaction of tetra-aniline **A**, longer tetra-aniline **C**, Zn(NTf_2_)_2_, and 2-formylpyridine in acetonitrile resulted in the formation of assembly **4** (Figure 4a). The crystal structure of **4** revealed its [Zn_8_L^**A**^_2_L^**C**^_4_]^16+^ architecture.^16^ The eight Zn^II^ centers, all having the same handedness (Δ, in Figure 4a), describe a twisted tetragonal prism. Tetra-aniline **A** residues panel two parallel faces, and **C** residues panel the remaining four quadrilateral faces.

**Figure 5:**
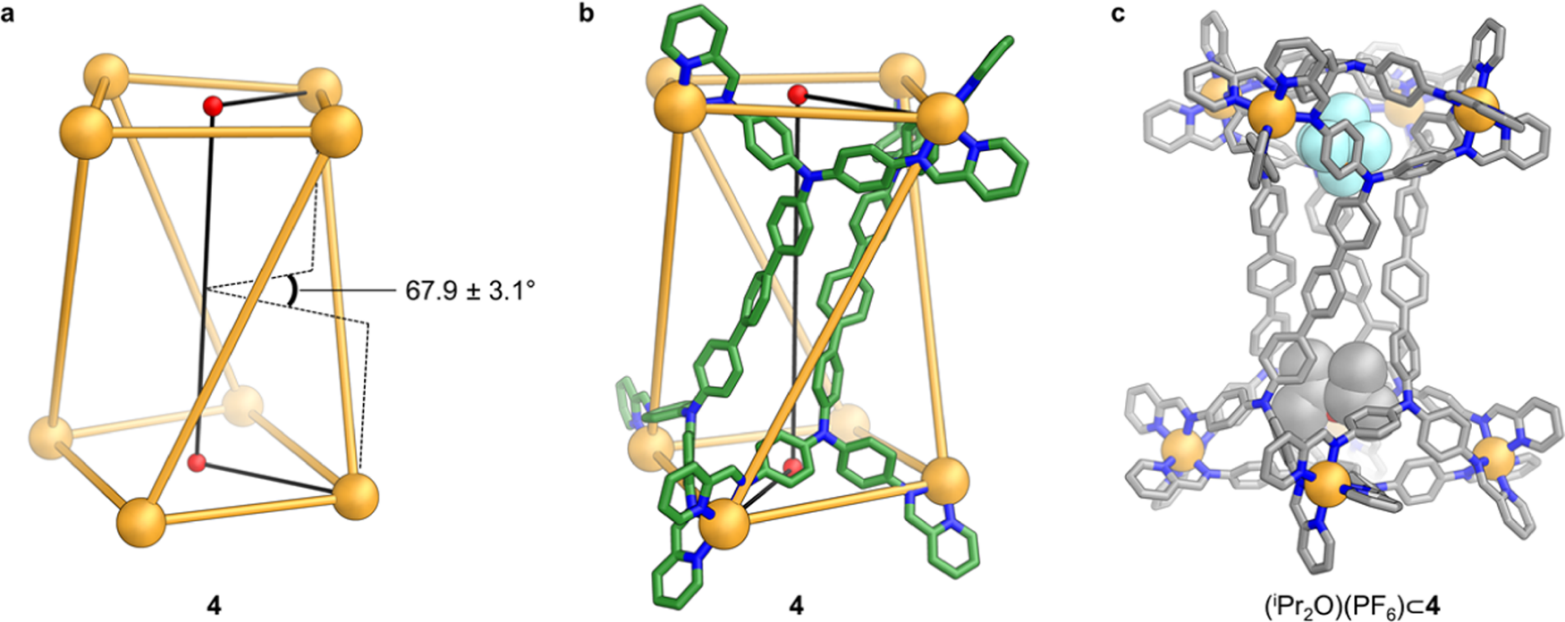
(a) Twist in tetragonal prism **4**, described by the mean Zn^II^ (top face)···centroid (top face)···centroid (bottom face)···Zn^II^ (bottom face) dihedral angle. The mean was calculated from the four values of this dihedral angle measured from the crystal structure. (b) Zn^II^_8_ framework, with two L^**C**^ ligands included, which illustrates that the Zn^II^ (top face) and Zn^II^ (bottom face) mean planes used to calculate the twist in the structure form a tetragonal prism edge where two **C** residues meet. Zn^II^: orange, N: blue, C: green. (c) X-ray crystal structure of **4** with PF_6_^–^ and diisopropyl ether (^i^Pr_2_O) residing at opposite ends of the internal cavity, which is effectively split onto two “pockets”. The ^i^Pr_2_O was modeled with partial occupancy. Disorder, anions (other than the bound PF_6_^–^), hydrogen atoms, and solvent molecules (other than the bound ^i^Pr_2_O) are omitted from the crystal structure for clarity. Zn^II^: orange, N: blue, C: gray, O: red, F: pale blue. The guest molecules are shown in space-filling mode.

Tetragonal prism **4** contains three distinct edge types (Figure 4b). At edges of type I, the short axis of an **A** residue meets the short axis of a **C** residue; the mean Zn^II^···Zn^II^ distance of 11.9 ± 0.1 Å for edge type I in structure **4** matches well with the observed distances in the analogous edge type in triangular prisms **1**–**3**. The long axes of two **C** residues meet at edge type II, with a mean Zn^II^···Zn^II^ distance of 22.2 ± 0.4 Å. The long axis of a tetra-aniline **A** residue meets the short axis of a **C** residue at edge type III, analogous to the edge type observed in the homoleptic Zn_8_L^**A**^_6_ pseudocube.^17^ The mean Zn^II^···Zn^II^ separation for this edge type in tetragonal prism **4** (12.5 ± 0.1 Å) is similar to that observed in the pseudocube (12.6 ± 0.2 Å). Structure **4** has a twist of 67.9 ± 3.1° (Figure 5). This twist causes a pinching inward of the terphenyl cores of the four **C** residues, creating a narrow channel connecting two wider pockets located at each end of the interior cavity of **4** (Figure S73). As shown in Figure 5c, in the crystal, these two pockets were observed to bind different guests. A diisopropyl ether molecule, modeled with partial occupancy, was located in one pocket, and a hexafluorophosphate (PF_6_^–^) anion in the other.

The ESI mass spectrum of **4** (Figures S53 and S54) confirmed its [Zn_8_L^**A**^_2_L^**C**^_4_]^16+^ composition in solution. The ^1^H NMR spectrum of **4** appeared to show two sets of signals (Figure S40), a major set and a minor set with lower integrated peak intensities. The ^1^H–^13^C HSQC spectrum (Figure S44) indicates that the major set has six imine signals, indicating that the corresponding structure has six magnetically distinct ligand arms. Reduced signal intensity and peak overlaps precluded us from determining the number of imine ^1^H signals for the minor species. The ^1^H DOSY diffusion constants for signals attributed to the minor species appeared similar to the values for the major one (Figure S48).

Based upon these observations, we thus infer the presence of two Zn_8_L^**A**^_2_L^**C**^_4_ diastereomers in solution, with a relative abundance of ca. 7:1 based upon ^1^H NMR signal integration (Figure S51). Varying the self-assembly conditions did not appear to significantly impact the observed diastereomeric ratio (Figure S52). At both reaction temperatures investigated (70 °C and 120 °C) no homoleptic species were detected in the ^1^H NMR spectra.T his absence of an effect of temperature on product composition, in contrast to a previously reported system,^2a^ precluded van ‘t Hoff analysis for determining the enthalpy and entropy changes of tetragonal prism formation.

The NMR spectroscopic data are consistent with one of the diastereomers corresponding to the structure of **4** in the crystal; however, this could be either the major or minor product. In the diastereomer observed in the crystal structure, the long axes of the two **A** residues run perpendicular to each other when the twist along the long axis of **4** is discounted. The other diastereomer may thus have the long axes of the two capping **A** residues aligned parallel (Figure S50). Both configurations have the same number of edge types I–III (Figure 4b), and thus should have a similar degree of strain.

The edge types observed in the structure of triangular prism **1** (Figure 2a) provide estimates for the preferred Zn^II^···Zn^II^ distances along the distinct axes of ligand L^**A**^, where the pair of Zn^II^ centers along a ligand side have the same handedness. Examination of the previously reported Zn_8_L^**A**^_6_ pseudocube^17^ alongside tetragonal prism **4** reveals that the same geometrical principle governs the formation of both structures: when the difference between preferred Zn^II^···Zn^II^ distances, Δ(Zn^II^···Zn^II^), is less than 2 Å, it is energetically favorable for different ligand sides to form together the edge of a polyhedron spanned by Zn^II^ centers with the same handedness. We anticipate that the value of Δ(M^II^···M^II^) at which the ligand sides can share polyhedron edges without incurring significant strain may decrease for cations with smaller ionic radii, and stricter preferences for adhering more closely to an ideal coordination geometry, for example, Fe^II^ (with a low-spin d^6^ electronic configuration).^19^

**Figure 6:**
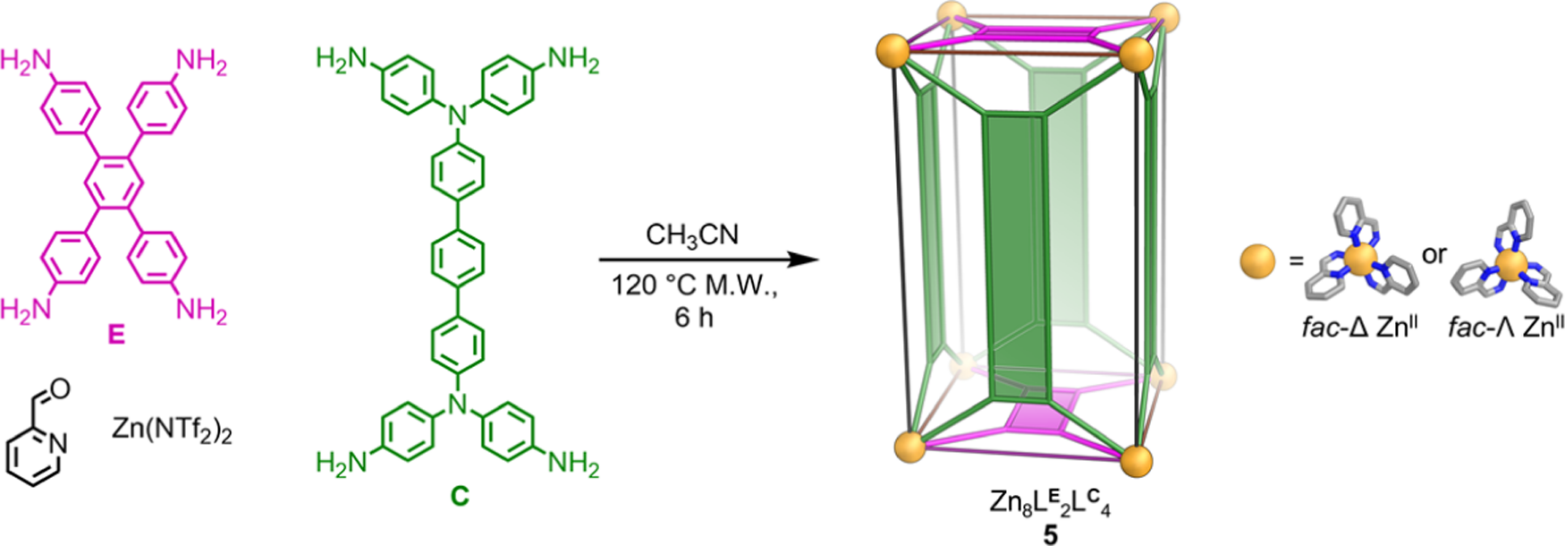
Subcomponent self-assembly of Zn_8_L^**E**^_2_L^**C**^_4_ heteroleptic tetragonal prism **5**, based upon the geometrical principles developed during this study.

From the crystal structure of the Zn_8_L^**E**^_6_ pseudocube,^17^ preferred Zn^II^···Zn^II^ distances along the unique rectangular axes of L^**E**^ (Figure 6), where the two Zn^II^ centers defining a common edge have the same handedness, were 10.4 ± 0.1 and 13.4 ± 0.1 Å. We thus hypothesized that the short axis of L^**C**^ could share an edge with either axis of L^**E**^ without incurring significant strain, despite the differing structure and geometry of L^**C**^. Replacing the two L^**A**^ panels in tetragonal prism **4** with L^**E**^ ligands would result in a tetragonal prism containing three distinct edge types, where (1) the short axis of L^**C**^ meets the short axis of L^**E**^, (2) the short axis of L^**C**^ meets the long axis of L^**E**^, and (3) the long axes of two **C** residues meet. All three of these edge types appear energetically feasible based on our observations thus far.

Tetragonal prism **5** was thus prepared *via* the subcomponent self-assembly of tetramines **E** and **C**, 2-formylpyridine, and Zn(NTf_2_)_2_ in acetonitrile (Figure 6). Crystals of **5** suitable for single-crystal X-ray diffraction were not obtained despite numerous attempts; however, ESI-MS data are consistent with the formation of a [Zn_8_L^**E**^_2_L^**C**^_4_]^16+^ assembly (Figures S71 and S72) and NMR spectroscopic data (Figures S55–S70) are consistent with **5** having a tetragonal prismatic structure similar to that of **4** (Figure 6). Based on these NMR spectra, we infer that **5** exists as two [Zn_8_L^**E**^_2_L^**C**^_4_]^16+^ diastereomers with similar relative abundances as observed for **4** (Figure S70). At 298 K, there was overlap and broadening of some signals in the ^1^H NMR spectrum of **5**. Increasing the temperature to 348 K (Figure S64) sharpened some signals, allowing the assignment of signals in the ^1^H NMR spectrum to proton environments on the ligands. Insights into other fluxional behavior in solution, such as dynamic twisting of **5**, could not be derived from the variable-temperature NMR spectroscopy study (Figures S64 and S65), however.

## Conclusions

Our geometric design strategy involves the matching of subcomponent sides with similar lengths to form edges of a polyhedron with a limited energetic penalty. Future work will focus on the preparation of assemblies that incorporate more than two distinct kinds of ligands by using the rules uncovered here.

Parallel studies are exploring the applications of the prolate, adaptable cavities of the prisms discussed herein, and structural analogues assembled using the design rules presented in this work.^20^ In particular, the simultaneous binding of two different types of guest molecules in separated binding pockets at each of end of the prismatic structures will be further explored.^21^ The helical twists of the heteroleptic prismatic structures reported in this work appear more pronounced than the twists of many of the hosts currently used for discriminating between the enantiomers of chiral guests.^22−24^ Future work will thus focus on making analogous heteroleptic cages stereospecifically.^25^ Furthermore, future work will also explore the potential photophysical functions of these metal–organic cages.^26^

## Supporting Information Available

Experimental procedures, NMR spectroscopy and mass spectrometry characterization data for new compounds, details of X-ray diffraction experiments, and details of the method for the calculation of the volumes of the internal cavities of the prismatic structures (PDF)
