# Supplementary material for: Triamine and Tetramine Edge-Length Matching Drives Heteroleptic Triangular and Tetragonal Prism Assembly
Source: J Am Chem Soc. 2024 Feb 13;146(8):5215–23. doi: 10.1021/jacs.3c11320 (PMC10910536; doi:10.1021/jacs.3c11320)
Supplement: Supplementary file 1 — ja3c11320_si_001.pdf [file ja3c11320_si_001.pdf]

Supporting Information

Triamine and Tetramine Edge-Length Matching Drives Heteroleptic Triangular and Tetragonal Prism Assembly

Jack A. Davies[1], Tanya K. Ronson[1] and Jonathan R. Nitschke[1]*

[1]Yusuf Hamied Department of Chemistry, University of Cambridge, Lensfield Road, Cambridge CB2 1EW, United Kingdom

Table of Contents

1 General Information S3
2 Synthesis of aniline subcomponents....S4
2.1 Subcomponent **C** S4
3 Synthesis and characterization of metal-organic cages S5
3.1 Synthesis and characterization of **1** S5
3.2 Synthesis and characterization of **2** S17
3.3 Synthesis and characterization of **3** S26
3.4 Synthesis and characterization of **4** S38
3.5 Synthesis and characterization of **5** S54
4 X-ray crystallography S67
5 Volume calculations S73
6 References....S73

1 General Information

Unless otherwise stated, all starting materials were sourced from commercial suppliers and used without further purification. Celite refers to AW Standard Super-Cel® NF supplied by Sigma-Aldrich. Self-assembly reactions were conducted in distilled acetonitrile. For the subcomponent self-assembly reactions irradiated with microwaves, a CEM Discover microwave reactor was used. For purification, acetonitrile solutions of the metal-organic cages were filtered through glass fibre.

NMR spectra were recorded using the following NMR spectrometers: Bruker 400 MHz Avance III HD Smart Probe (routine ^{1}H NMR, ^{13}C NMR, ^{19}F NMR and ^{1}H DOSY), 500 MHz Avance III Smart Probe (routine ^{1}H NMR), 500 MHz DCH Cryoprobe (High resolution ^{1}H, ^{13}C and 2D NMR experiments), Bruker 500 MHz Avance III HD Smart Probe (variable temperature ^{1}H NMR study, increased temperature ^{1}H-^{1}H COSY and NOESY), 700 MHz TXO Cryoprobe (High resolution ^{1}H, ^{13}C and 2D NMR experiments). Chemical shifts (δ) were reported in parts per million (ppm) for ^{1}H, ^{13}C and ^{19}F NMR spectra. Chemical shifts were referenced using the residual CD_3CN solvent signal (^{1}H = 1.94 ppm, ^{13}C = 118.26 ppm) or d_6-DMSO solvent signal (^{1}H = 2.50 ppm, ^{13}C = 39.52 ppm). A C_6F_6 internal standard was added for ^{19}F NMR spectra, with the signal referenced at -164.38 ppm.[1] Coupling constants (*J*) were reported in Hz to 1 decimal place. Signal multiplicity in ^{1}H and ^{13}C NMR spectra was described using the following abbreviations: singlet (s), doublet (d), triplet (t), quartet (q), doublet of doublets (dd), triplet of doublets (td), doublet of doublet of doublets (ddd), multiplet (m), broad (br) and apparent (app.).

^{1}H DOSY NMR experiments were conducted on a Bruker 400 MHz Avance III HD Smart Probe spectrometer. Maximum gradient strength was 5.35 G/cm A. The standard Bruker pulse program, ledbpgp2s,[2] employing a stimulated echo and longitudinal eddy-current delay (LED) using bipolar gradient pulses for diffusion using 2 spoil gradients, was utilized. A gradient ramp of 5% to 90% was used. d20 was set to 0.1 s and p30 was optimised for each species, p30 = 1300 μs for **1**·$(NTf_2)_{12}$ and **3**·$(NTf_2)_{12}$, 1200 μs for **2**·$(NTf_2)_{12}$, 1450 μs for **4**·$(NTf_2)_{16}$ and **5**·$(NTf_2)_{16}$.

Edited HSQC sequence results in cross-peaks for CH/CH_3 and CH_2 having opposite phases.

High resolution electrospray ionisation mass spectra (ESI–HRMS), for subcomponent **C** and acetonitrile solutions of metal-organic architectures **1**–**5**, were recorded on a Waters Synapt G2-Si instrument.

2 Synthesis of aniline subcomponents

Subcomponent **A** was purchased from Carbosynth (*N,N,N',N'*-Tetrakis(4-aminophenyl)-1,4-phenylenediamine) and used without further purification. Subcomponent **D** was purchased from TCI and used without further purification. Reported procedures were followed for the synthesis of subcomponents **B** and **E**.[3]

2.1 Subcomponent C

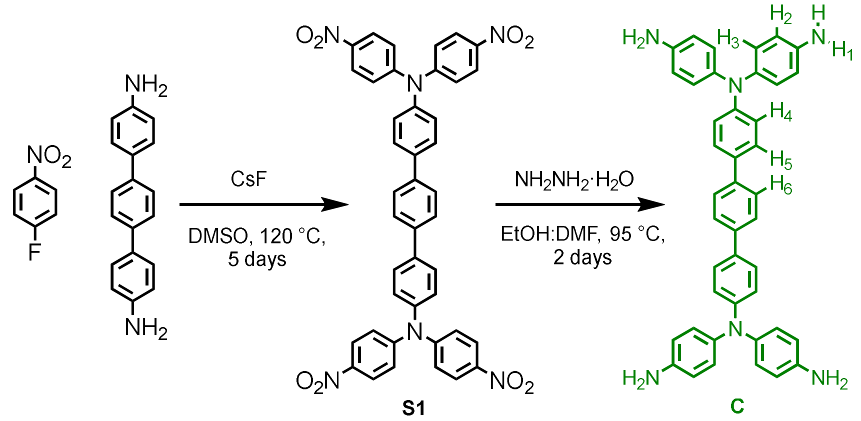

Scheme S1. Synthetic route for subcomponent **C**

An oven-dried Schlenk flask was charged with 4,4''-diamino-p-terphenyl (0.60 g, 2.3 mmol, 1.0 equiv), caesium fluoride (2.10 g, 13.8 mmol, 6.0 equiv), dried dimethyl sulfoxide (23 mL) and 1-Fluoro-4-nitrobenzene (1.6 mL, 15.1 mmol, 6.6 equiv). The mixture was degassed by performing four freeze-pump-thaw cycles and the reaction mixture was heated under a nitrogen atmosphere at 120 °C for 5 days. The orange suspension was allowed to cool to room temperature and water (400 mL) was added. After standing overnight, the solid was obtained by filtration, and washed with water and diethyl ether several times. The crude precursor **S1** was obtained as an orange/yellow solid and used in the following step without further purification.

A mixture of **S1** (304 mg, 0.41 mmol, 1.0 equiv), ethanol (24 mL), *N*,*N*-Dimethylformamide (DMF) (6 mL) and hydrazine monohydrate (1.5 mL, 30.9 mmol, 75 equiv) was degassed by bubbling nitrogen for 30 minutes. To the reaction mixture, 10 wt% Pd/C (125 mg) was added, the reaction mixture was degassed by bubbling nitrogen for 30 minutes and heated at 95 °C under a nitrogen atmosphere for 44 hours. The reaction mixture was allowed to cool to room temperature and water was added to induce precipitation. The resulting solid was collected by vacuum filtration, washed with copious water and DMF was added to the solid. The DMF mixture was passed over a celite plug and the DMF was removed *in vacuo*. The resulting solid was washed with copious diethyl ether. The product **C** was obtained as a dark green solid (191 mg, 0.31 mmol, 76%).

^{1}H NMR (400 MHz, DMSO-d_6, 298 K) δ 7.55 (s, 4H), 7.41 (d, J = 8.8 Hz, 4H), 6.84 (d, J = 8.6 Hz, 8H), 6.65 (d, J = 8.8 Hz, 4H), 6.55 (d, J = 8.6 Hz, 8H), 4.98 (s, 8H).

^{13}**C NMR** (101 MHz, DMSO-d_6, 298 K) δ 149.1, 145.8, 137.9, 135.8, 128.9, 127.6, 126.7, 126.0, 116.9, 115.0.

ESI-MS Found m/z = 624.3047 $[M]^+$, $C_{42}H_{36}N_6$ requires m/z = 624.3001.

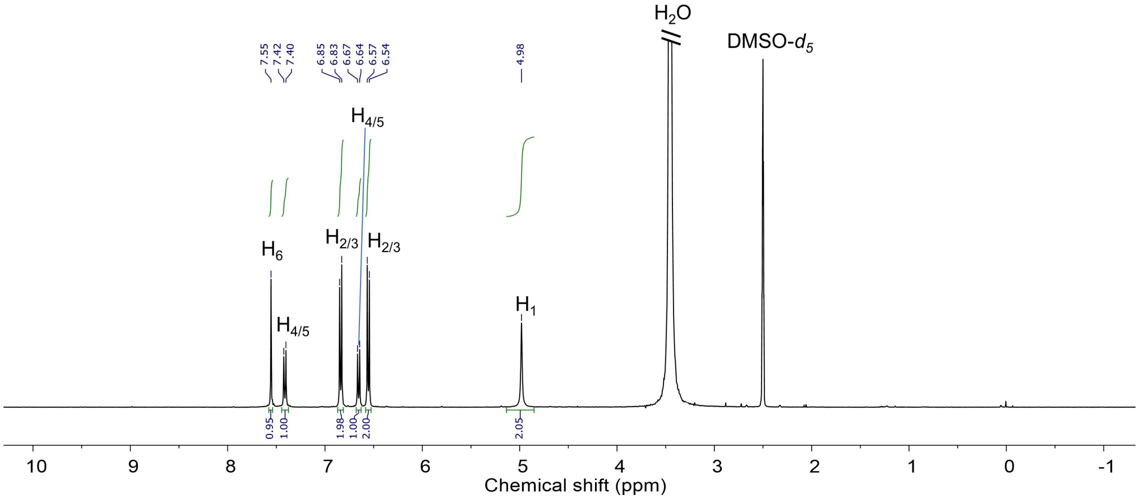

Figure S1. ^{1}H NMR spectrum (400 MHz, DMSO-d_6, 298 K) of **C**, with assignment of signals.

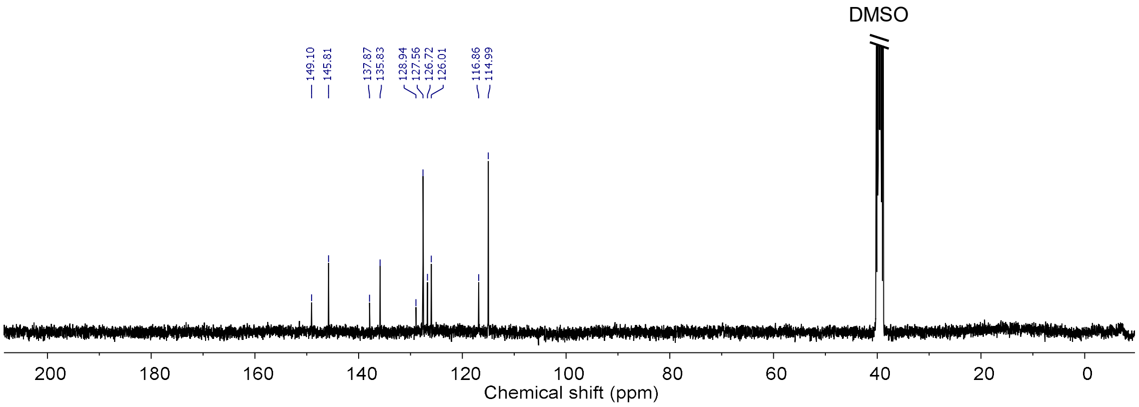

Figure S2. ^{13}C NMR spectrum (101 MHz, DMSO-d_6, 298 K) of **C**.

3 Synthesis and characterization of metal-organic cages

3.1 Synthesis and characterization of 1

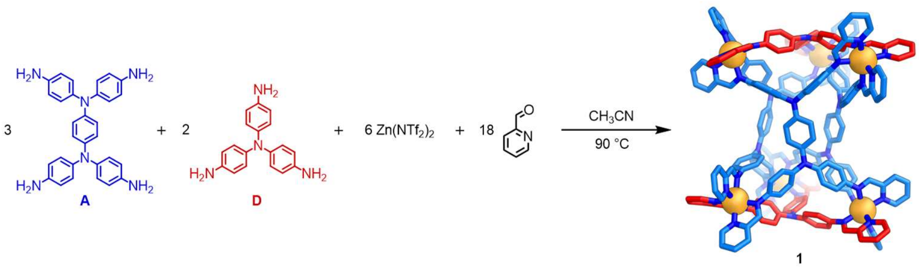

Scheme S2. Subcomponent self-assembly of **1**·$(NTf_2)_{12}$.

To a mixture of tetra-aniline **A** (21.2 mg, 45 μmol, 2.6 equiv), tri-aniline **D** (5.0 mg, 17 μmol, 1.0 equiv) and $Zn(NTf_2)_2$ (49.1 mg, 78 μmol, 4.6 equiv) in distilled acetonitrile (2.5 mL), 2-formylpyridine (23.0 μL, 240 μmol, 14 equiv) was added. The reaction mixture was heated at 90 °C for 24 hours. The reaction mixture was allowed to cool to room temperature and the insoluble byproducts were removed by centrifugation and filtration through a glass fibre plug. The mixture was concentrated to a small volume and diethyl ether (8 mL) was added. The solid was washed with diethyl ether (3 × 8 mL) and the product **1**·$(NTf_2)_{12}$ was obtained as a very dark red/orange solid (70.6 mg, quant.).

^{1}H NMR (500 MHz, CD_3CN, 298 K) δ 8.70 (s, 6H), 8.49–8.42 (m, 24H), 8.34–8.29 (m, 12H), 8.24 (d, *J* = 7.8 Hz, 6H), 8.08 (d, *J* = 7.8 Hz, 6H), 8.02 (d, *J* = 5.0 Hz, 6H), 7.98 (d, *J* = 5.0 Hz, 6H), 7.90 (ddd, *J* = 7.8, 5.1, 1.1 Hz, 6H), 7.86 (ddd, *J* = 7.8, 5.1, 1.2 Hz, 6H), 7.78 (ddd, *J* = 7.9, 5.1, 1.2 Hz, 6H), 7.58 (d, *J* = 5.0 Hz, 6H), 7.08 (d, *J* = 8.7 Hz, 12H), 7.04 (d, *J* = 8.6 Hz, 12H), 6.97 (s, 12H), 6.80 (app. br s, 12H), 6.32 (d, *J* = 8.6 Hz, 12H), 6.26 (d, *J* = 9.1 Hz, 12H), 5.88 (d, *J* = 7.9 Hz, 12H).

^{13}C NMR (126 MHz, CD_3CN, 298 K) δ 166.8, 165.4, 163.7, 150.7, 150.3, 149.6, 147.4, 147.2, 147.1, 147.0, 147.0, 146.9, 146.0, 144.1, 143.6, 143.5, 143.3, 143.1, 140.6, 132.3, 132.0, 131.6 (2 x ^{13}C), 131.5, 131.4, 131.3, 127.0, 124.7, 124.1, 123.9, 120.8 (q, *J* = 321 Hz, $^-NTf_2$), 117.0. (The ^{13}C signal for the carbon bonded to H_7 was not detected, which we attribute to dynamic processes occurring at room temperature).

^{19}F NMR (376 MHz, CD_3CN, 298 K) δ −79.90.

ESI-HRMS ([**1**$(NTf_2)_{12}$] = $C_{234}H_{174}N_{44}Zn_6(C_2F_6NO_4S_2)_{12}$) *m/z* = 770.6639 [**1**$(NTf_2)_5]^{7+}$ (calc. 770.6645), 945.7633 [**1**$(NTf_2)_6]^{6+}$ (calc. 945.7612), 1190.9018 [**1**$(NTf_2)_7]^{5+}$ (calc. 1190.8972), 1558.8586 [**1**$(NTf_2)_8]^{4+}$ (calc. 1558.8506), 2171.7895 [**1**$(NTf_2)_9]^{3+}$ (calc. 2171.7737).

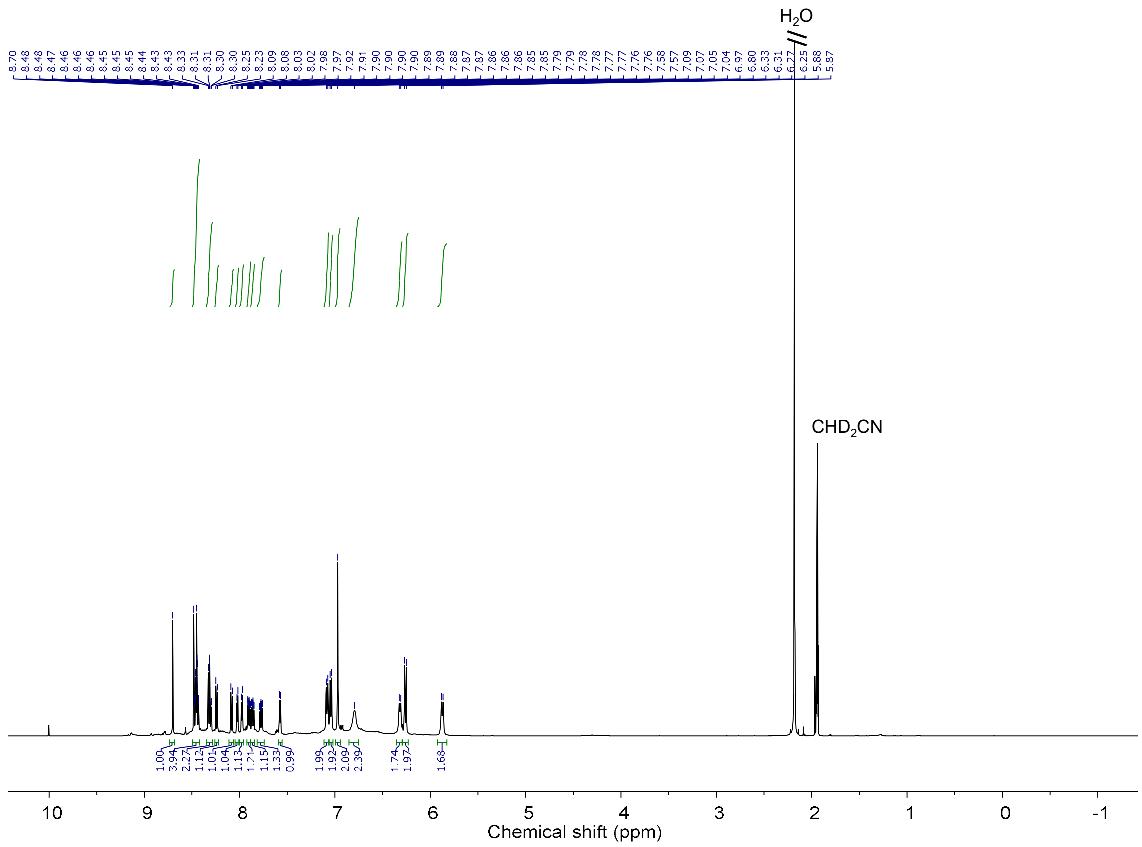

Figure S3. ^{1}H NMR spectrum (500 MHz, CD_3CN, 298 K) of **1**·$(NTf_2)_{12}$.

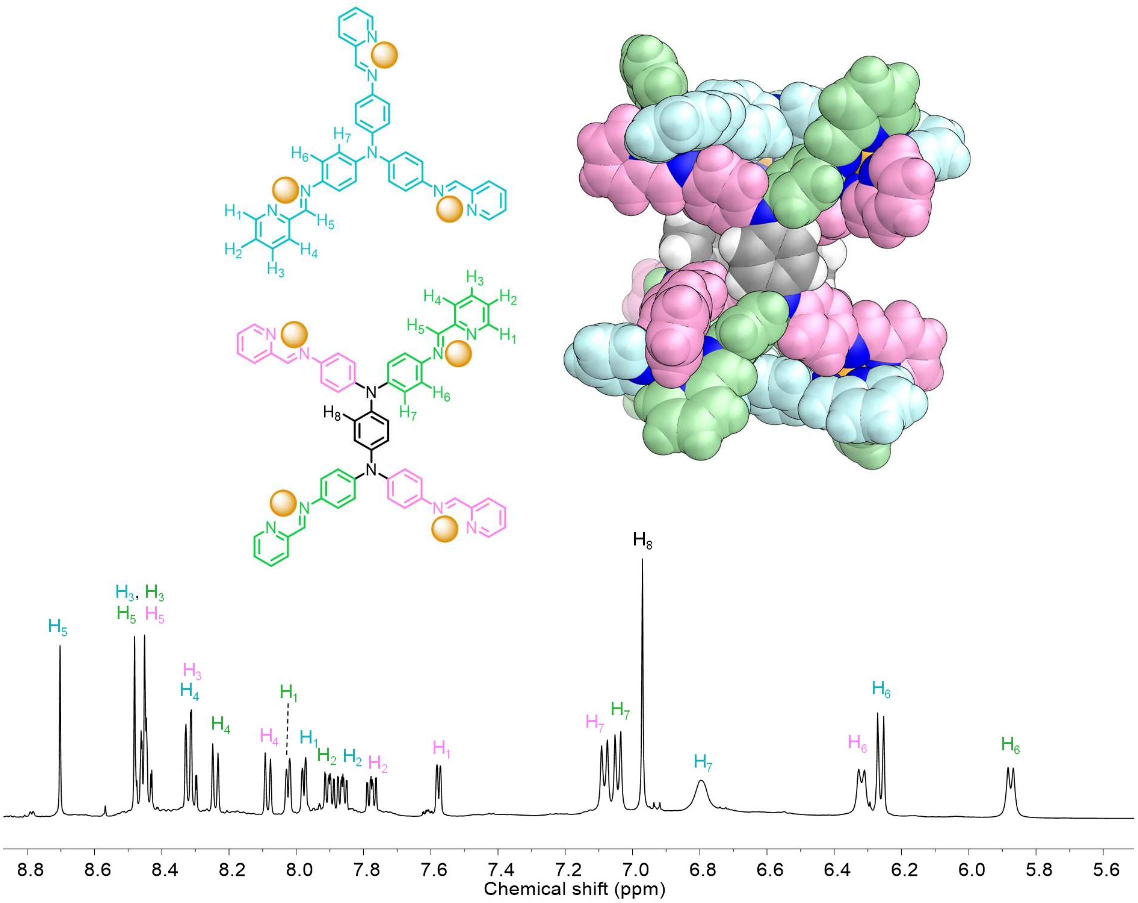

Figure S4. Aromatic region of the ^{1}H NMR spectrum (500 MHz, CD_3CN, 298 K) of **1**·$(NTf_2)_{12}$, with assignment of signals. The ^{1}H-^{13}C edited HSQC spectrum (Figure S8) enabled identification of the three imine (H_5) signals. The ^{1}H-^{1}H NOESY spectrum (Figure S9) then allowed identification of the signals for the three H_4 and H_6 proton environments. The ^{1}H-^{1}H DQF-COSY spectrum (Figure S7) enabled completion of the identification of the three sets of signals for the proton environments H_1-H_7 on the three unique ligand arms. Finally, assignment of the ^{1}H NMR signals to specific proton environments in structure **1** was carried out using the 1H-1H NOESY spectrum (Figure S9).

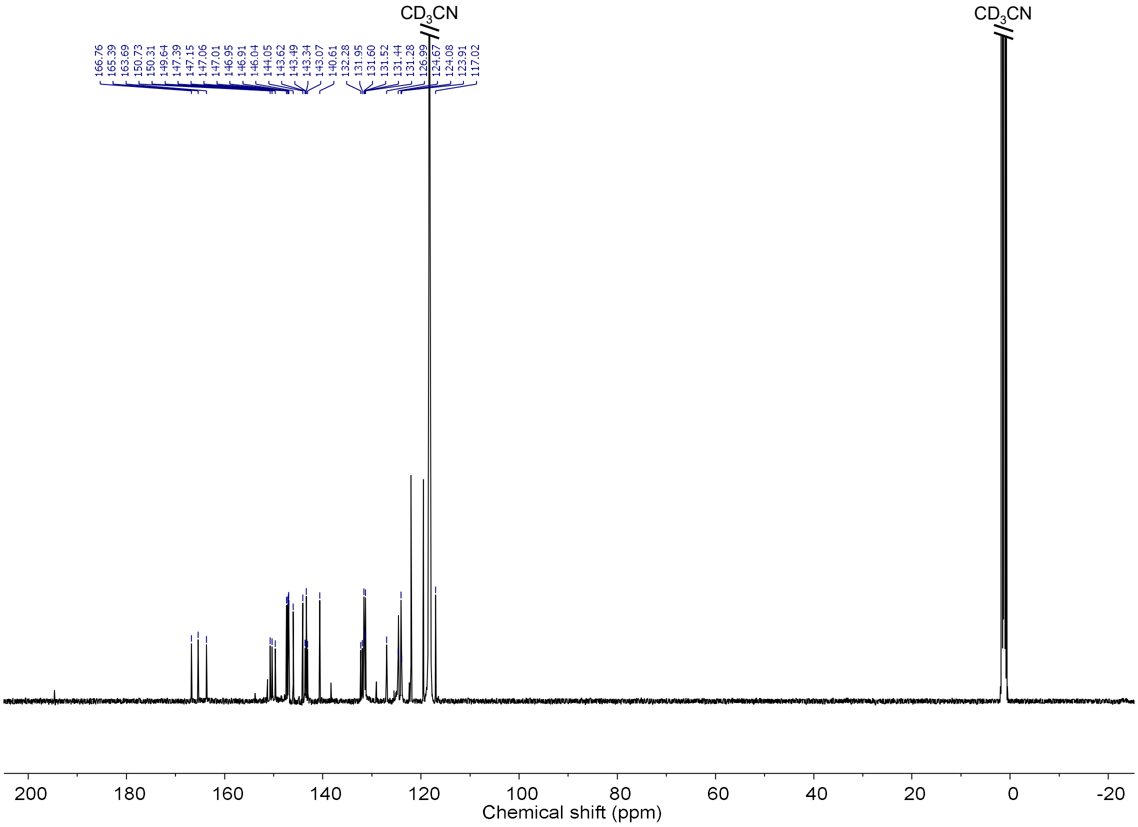

Figure S5. ^{13}C NMR spectrum (126 MHz, CD_3CN, 298 K) of **1**·$(NTf_2)_{12}$.

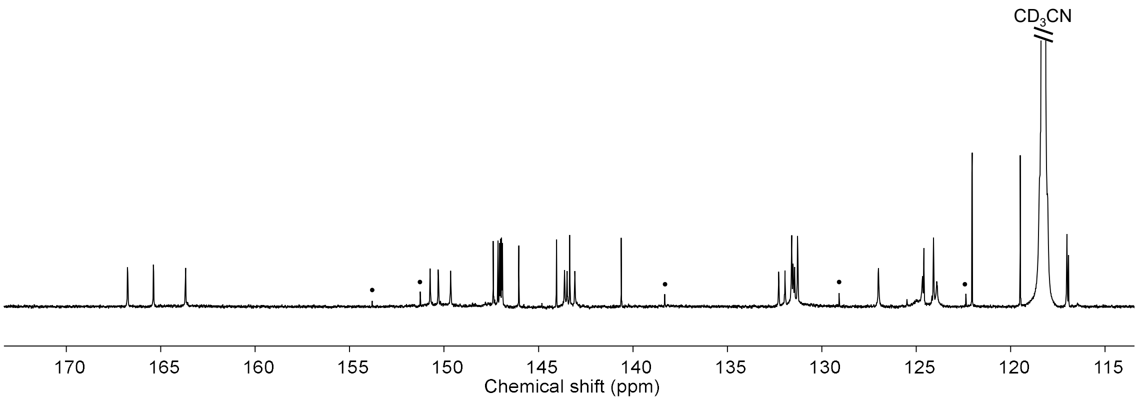

Figure S6. Aromatic region of the ^{13}C NMR spectrum (126 MHz, CD_3CN, 298 K) of **1**·$(NTf_2)_{12}$. Black circles indicate signals for the ^{13}C environments in residual 2-formylpyridine.

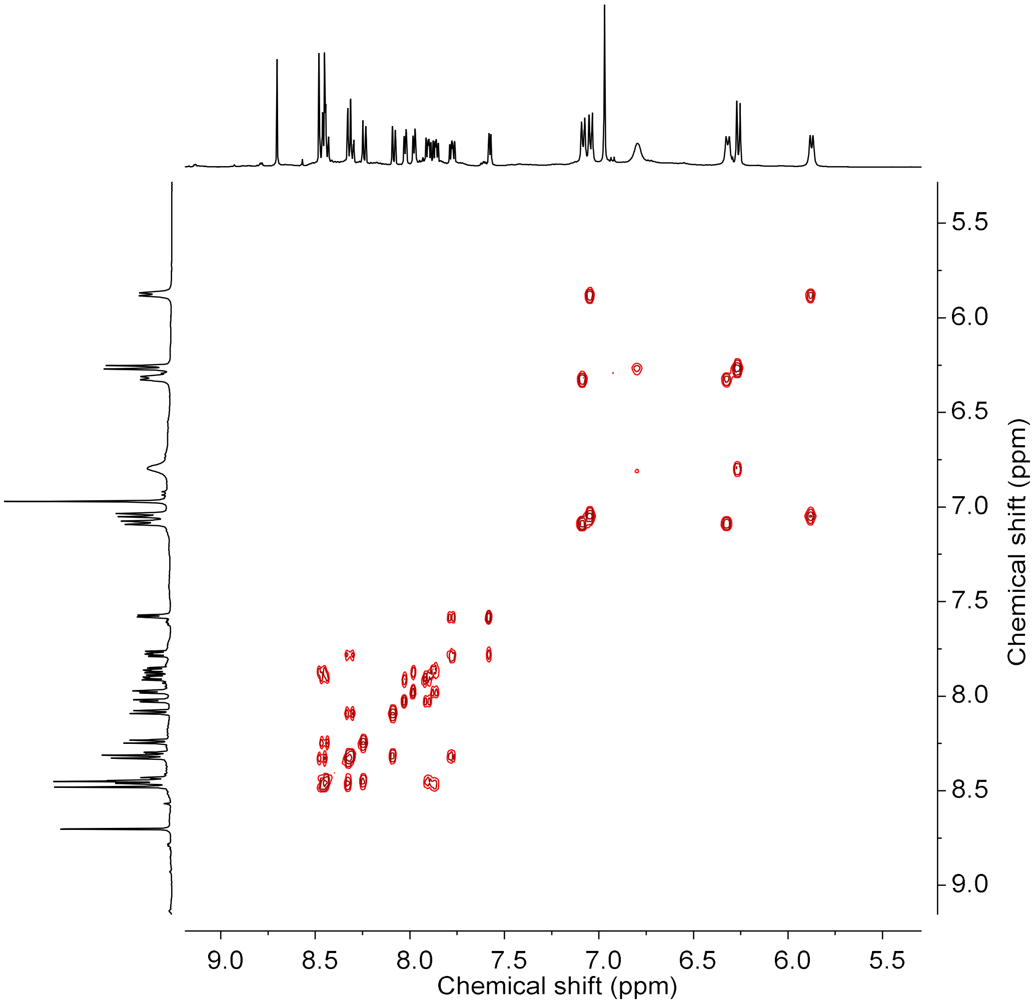

Figure S7. Aromatic region of the ^{1}H-^{1}H DQF-COSY spectrum (500 MHz, CD_3CN, 298 K) of **1**·$(NTf_2)_{12}$.

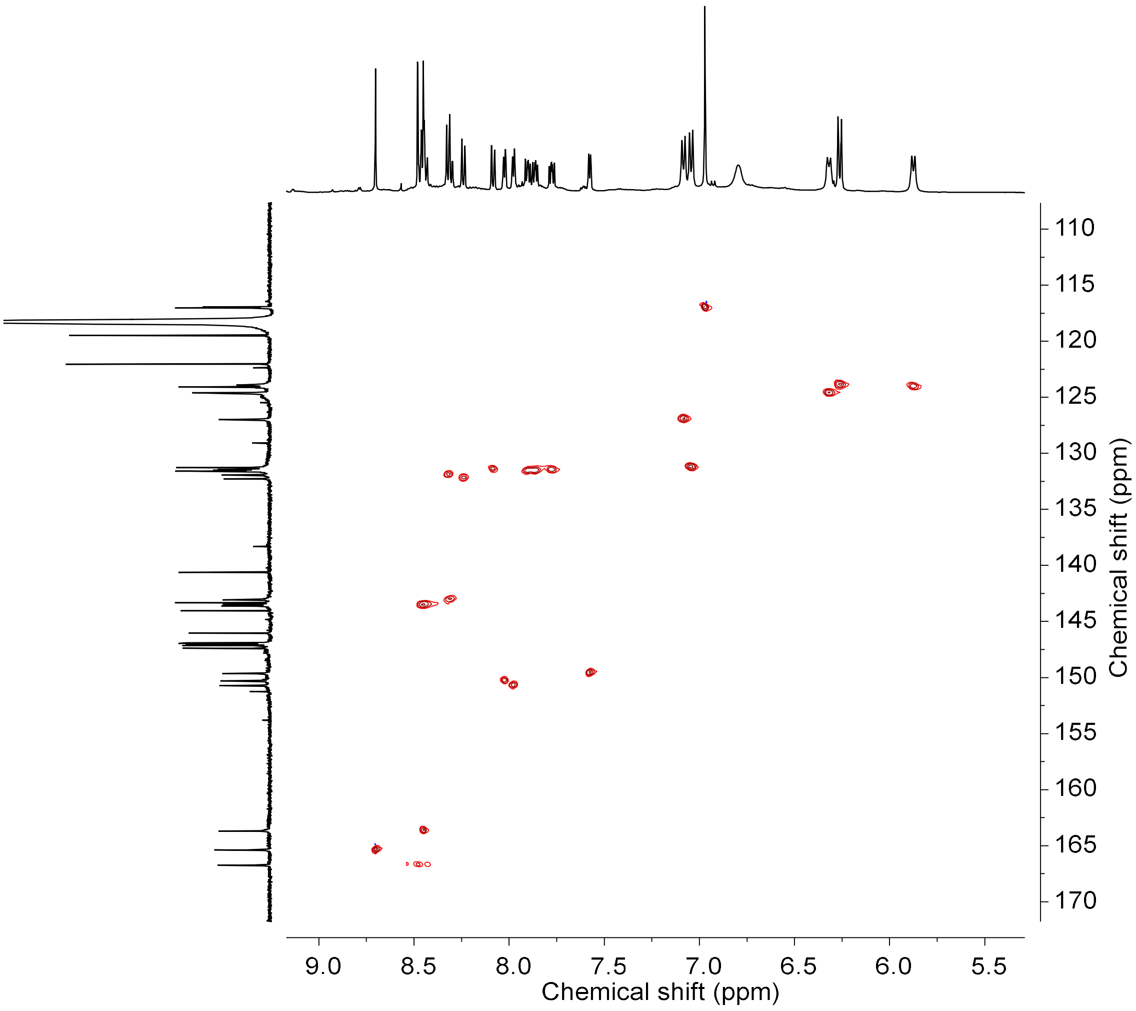

Figure S8. Aromatic region of the ^{1}H-^{13}C edited HSQC spectrum (500 MHz, CD_3CN, 298 K) of **1**·$(NTf_2)_{12}$.

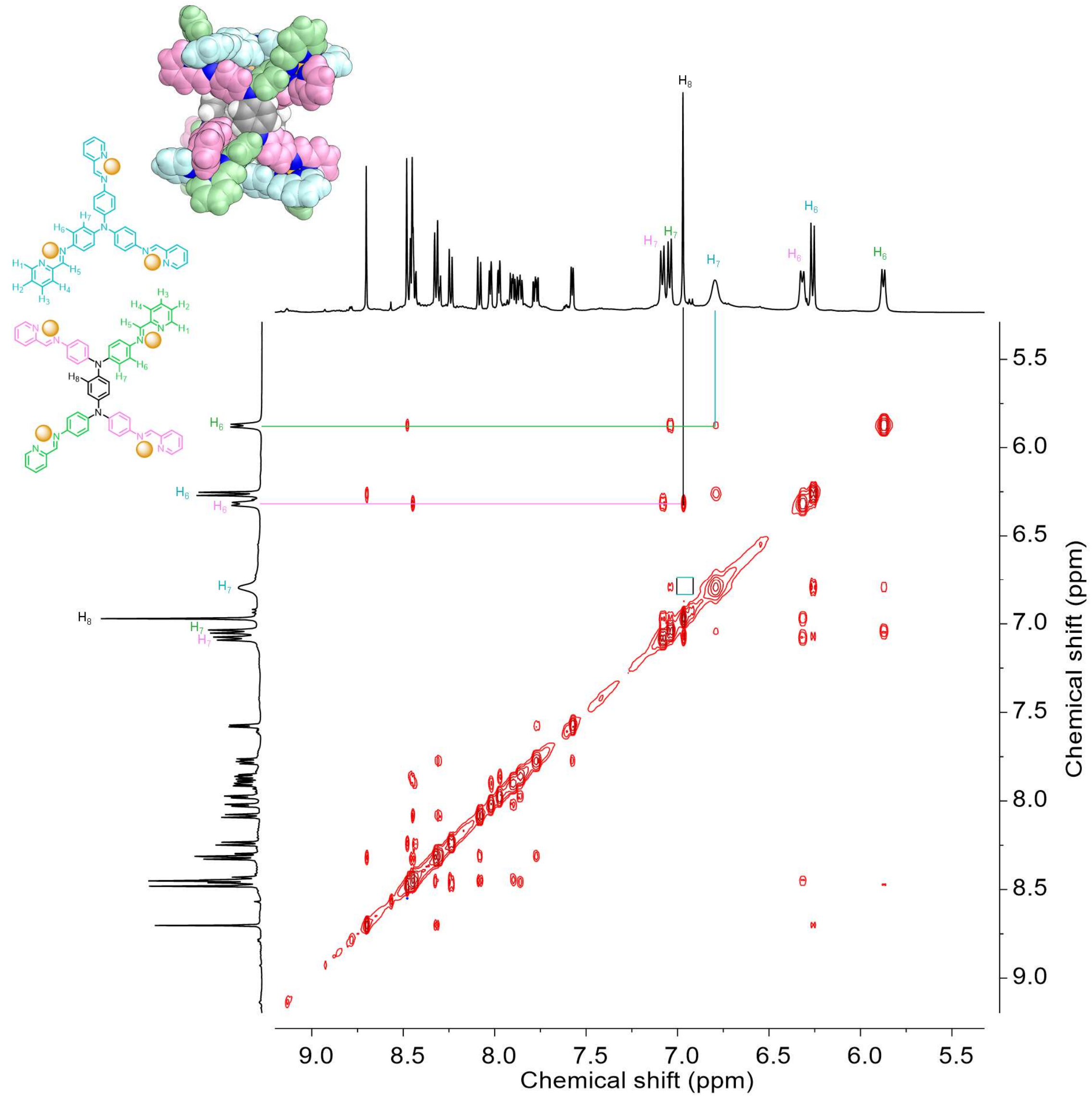

Figure S9. Aromatic region of the ^1H-^1H NOESY spectrum (500 MHz, CD_3CN, 298 K) of **1**·$(NTf_2)_{12}$ with the presence/absence of key NOE correlations for the assignment of the three sets of ^1H NMR signals to specific ligand arms in **1** highlighted.

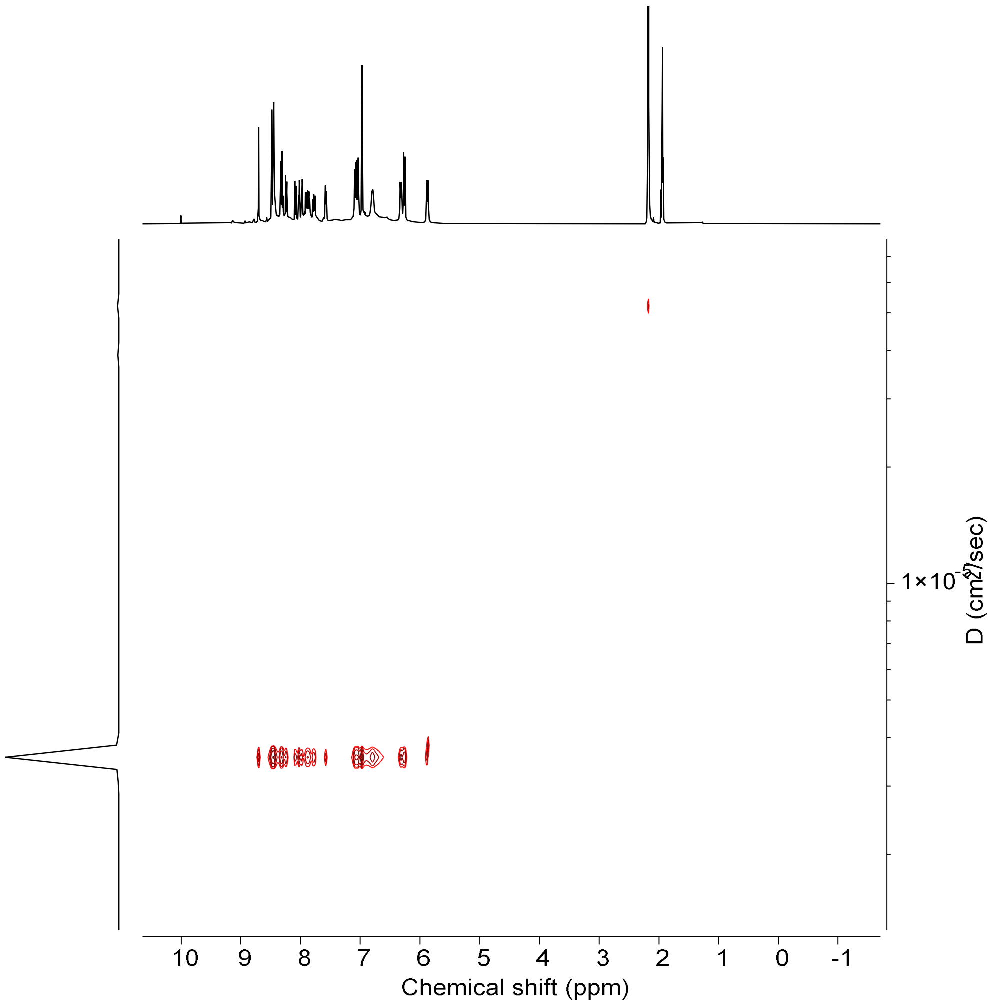

Figure S10. ^{1}H DOSY spectrum (400 MHz, CD_3CN, 298 K) of **1**·$(NTf_2)_{12}$.

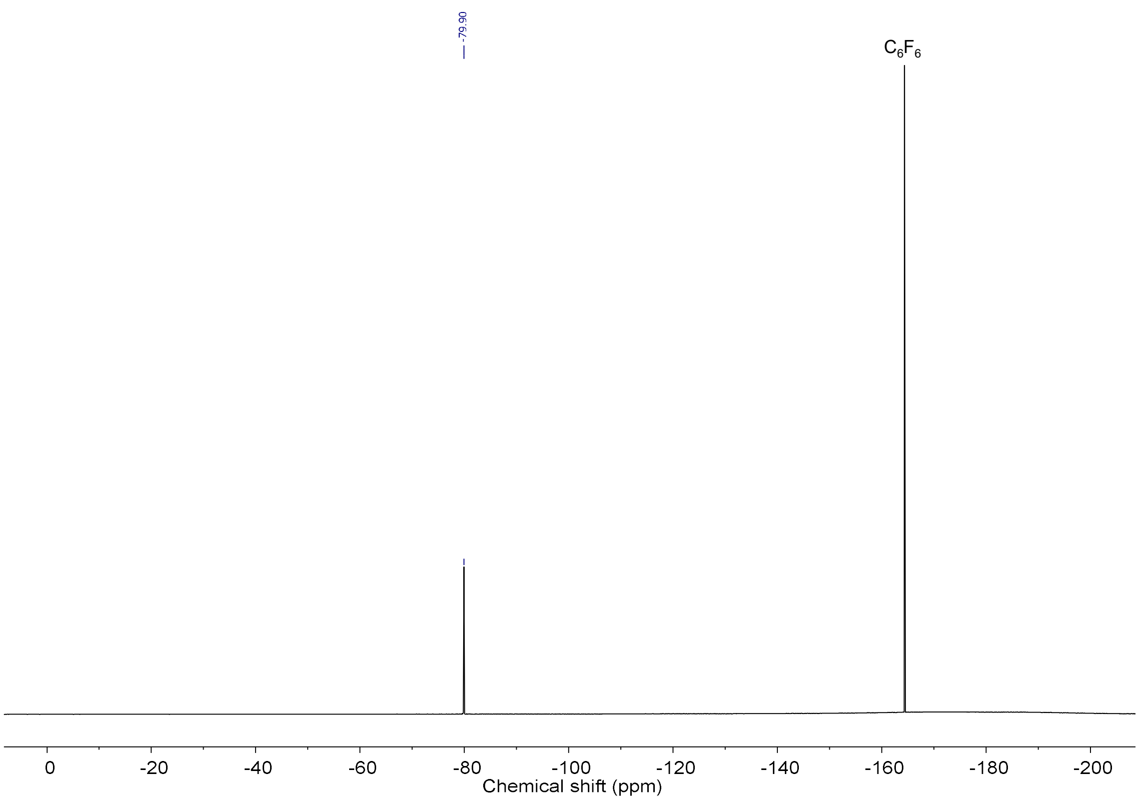

Figure S11. ^{19}F NMR spectrum (376 MHz, CD_3CN, 298 K) of **1**·$(NTf_2)_{12}$.

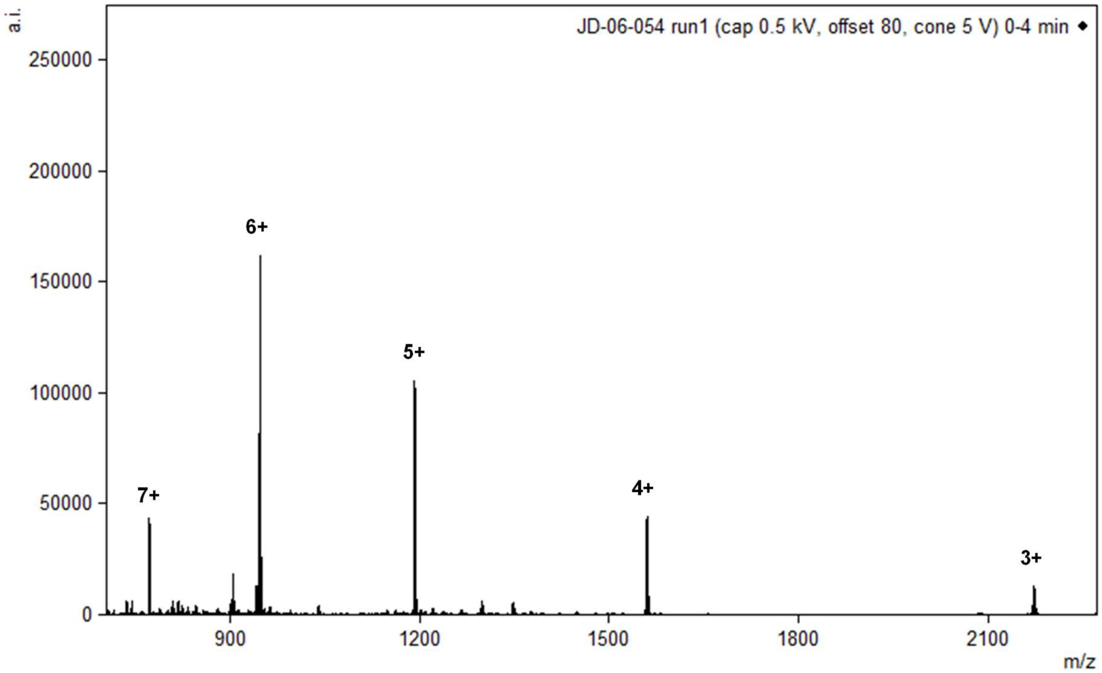

Figure S12. High resolution ESI-mass spectrum for **1**·$(NTf_2)_{12}$.

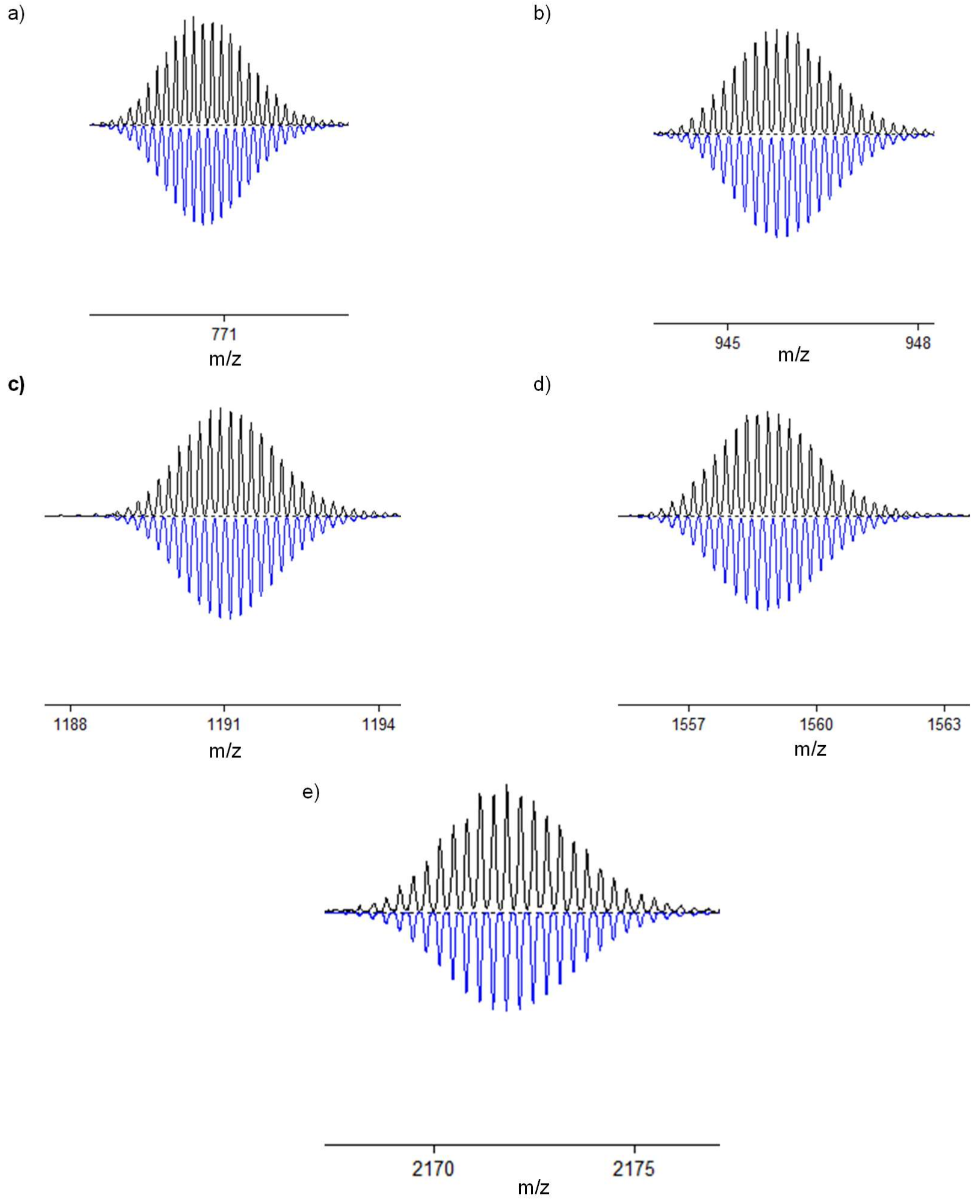

Figure S13. Signals from the high resolution ESI-mass spectrum for **1**·$(NTf_2)_{12}$. Experimental (black) and calculated (blue) signals for a) [**1**$(NTf_2)_5]^{7+}$ b) [**1**$(NTf_2)_6]^{6+}$ c) [**1**$(NTf_2)_7]^{5+}$ d) [**1**$(NTf_2)_8]^{4+}$ e) [**1**$(NTf_2)_9]^{3+}$.

Investigating the subcomponent self-assembly of triangular prism 1

We initially attempted the synthesis of **1** using stoichiometric quantities of subcomponents (i.e 1.5 equiv of tetra-aniline **A**). After heating the reaction mixture at 90 °C for 20 hours, peaks corresponding to the $Zn_4L^{\mathbf{D}}_4$ tetrahedron were observed in the ^{1}H NMR spectrum of the reaction mixture. Extra tetra-aniline **A** (with the corresponding additional $Zn(NTf_2)_2$ and 2-formylpyridine) were added and the reaction mixture was heated prior to the acquisition of its ^{1}H NMR spectrum. This process was repeated until peaks for the tetrahedron were no longer observed (Figure S14). Gradual addition of the extra

subcomponents versus addition at the start of the reaction (as described above in the synthetic procedure for **1**) appeared to have little effect on the purity of the final product.

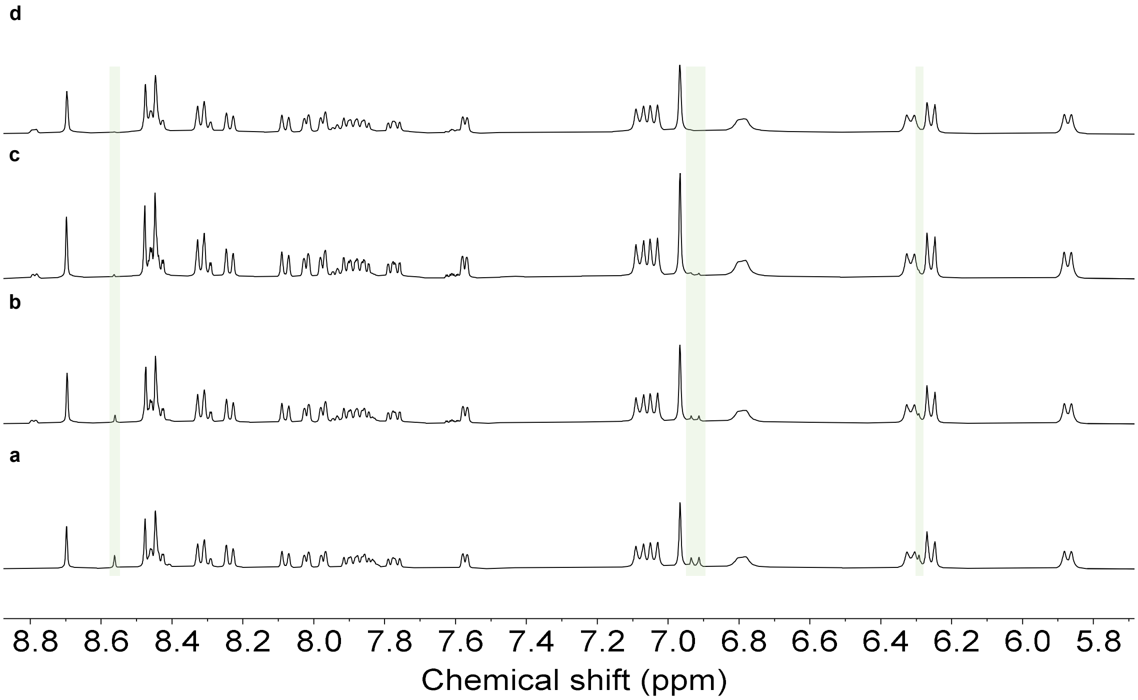

Figure S14. Partial ^{1}H NMR spectra (400 MHz, CD_3CN, 298 K) of aliquots of the subcomponent self-assembly reaction mixture for the preparation of **1**·$(NTf_2)_{12}$, after a) mixing of tri-aniline **D** (10.0 mg, 34 µmol, 1.0 equiv), tetra-aniline **A** (24.5 mg, 52 µmol, 1.5 equiv), $Zn(NTf_2)_2$ (68.0 mg, 110 µmol, 3.2 equiv) and 2-formylpyridine (32.0 µL, 340 µmol, 10 equiv) in distilled acetonitrile (5 mL), and heating at 90 °C for 20 hours. b) Addition of tetra-aniline **A** (6.0 mg, 13 µmol, 0.4 equiv), $Zn(NTf_2)_2$ (11.1 mg, 18 µmol, 0.5 equiv) and 2-formylpyridine (5.0 µL, 53 µmol, 1.6 equiv), and the reaction mixture was heated at 90 °C for 7 hours. c) Addition of tetra-aniline **A** (5.9 mg, 12 µmol, 0.4 equiv), $Zn(NTf_2)_2$ (11.2 mg, 18 µmol, 0.5 equiv) and 2-formylpyridine (5.0 µL, 53 µmol, 1.6 equiv), and the reaction mixture was heated at 90 °C for 14 hours. d). Tetra-aniline **A** (6.0 mg, 13 µmol, 0.4 equiv), $Zn(NTf_2)_2$ (11.1 mg, 18 µmol, 0.5 equiv) and 2-formylpyridine (5.0 µL, 53 µmol, 1.6 equiv) were added. The reaction mixture was heated at 90 °C for 6 hours. Green boxes highlight ^{1}H NMR signals that are attributed to the $Zn_4L^{\mathbf{D}}_4$ tetrahedron, the other signals for this species are not easily identifiable in the spectra a–d due to overlap with signals for the $Zn_6L^{\mathbf{A}}_3L^{\mathbf{D}}_2$ triangular prism. The ratio of the integrated peak intensities for the signal at δ = 8.70 ppm for the heteroleptic triangular prism and δ = 8.56 ppm for the homoleptic tetrahedron are 1:0.29, 1:015 and 1:0.06 in spectra a–c, respectively. The analogous ratio in the ^{1}H NMR spectrum for **1** synthesized following the procedure on page S6 is approximately 1:0.07.

Using a batch of subcomponent **A** synthesised by ourselves, or heating the subcomponent self-assembly reaction for a longer time (4 days), did not circumvent the requirement for the use of excess subcomponent **A** (and $Zn(NTf_2)_2$ and 2-formylpyridine).

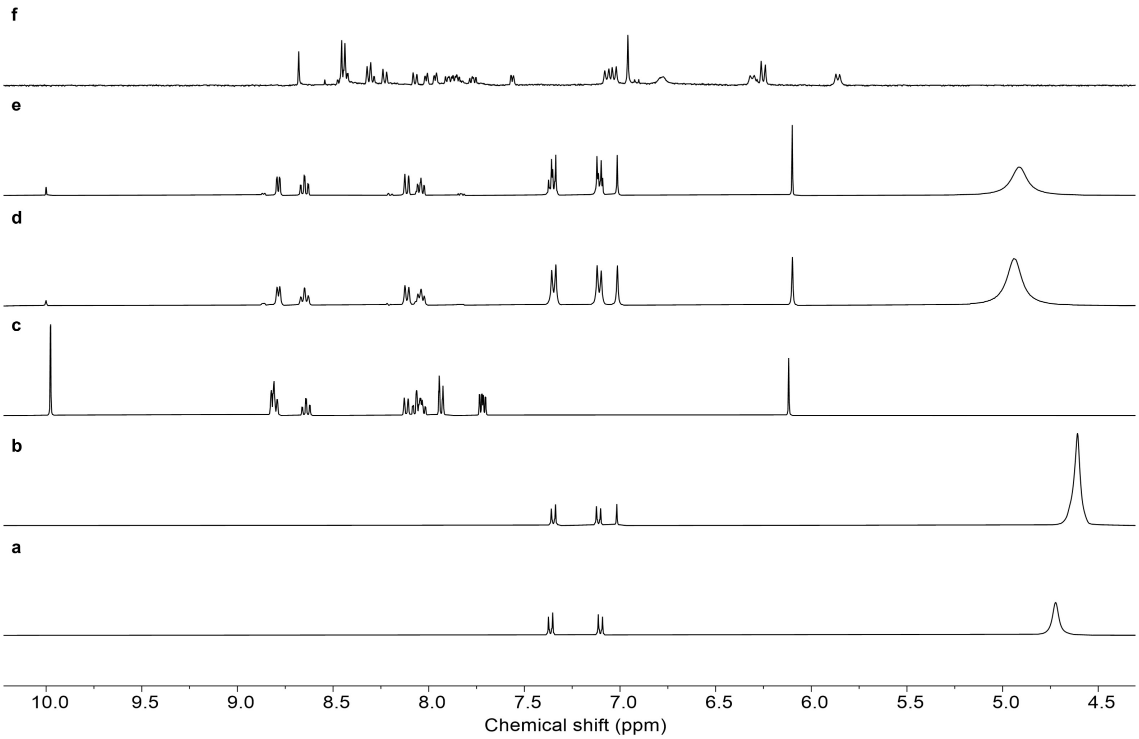

Figure S15. Digestion experiment. a–e) Partial ^{1}H NMR spectra (400 MHz, DMSO-d_6 containing DCl, 298 K). 20 µL of DCl (20 wt% in D_2O) was added to each sample dissolved in DMSO-d_6. a) Tri-aniline **D** b) Tetra-aniline **A** c) 2-formylpyridine d) Insoluble material formed during the preparation of triangular prism **1** e) Triangular prism **1** f) Partial ^{1}H NMR spectrum (400 MHz, CD_3CN, 298 K) of the batch of **1** used for the digestion experiment.

3.2 Synthesis and characterization of 2

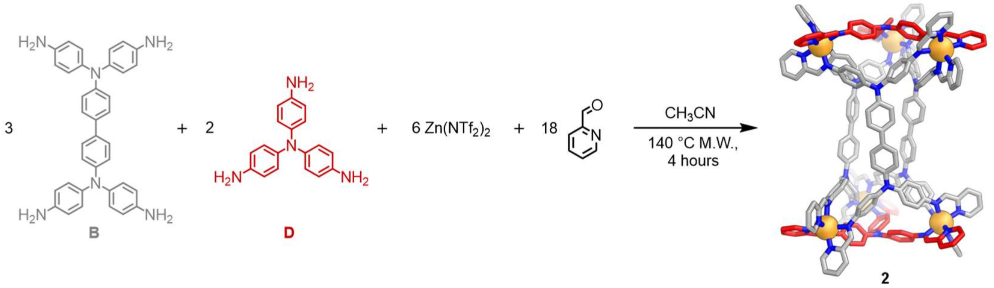

Scheme S3. Subcomponent self-assembly of **2**·$(NTf_2)_{12}$.

To a mixture of tetra-aniline **B** (6.4 mg, 11.7 µmol, 1.7 equiv), tri-aniline **D** (2.0 mg, 6.9 µmol, 1.0 equiv) and $Zn(NTf_2)_2$ (14.5 mg, 23.2 µmol, 3.4 equiv) in acetonitrile (4.0 mL), 2-formylpyridine (6.9 µL, 72.5 µmol, 10.5 equiv) was added. The reaction mixture was heated at 140 °C for 4 hours in a microwave reactor. The reaction mixture was allowed to cool to room temperature, filtered through a glass fibre plug, concentrated to a small volume and diethyl ether (8 mL) was added. The solid was

washed with diethyl ether (3 × 8 mL) and the product **2**·$(NTf_2)_{12}$ was obtained as a very dark red/orange solid (26.6 mg, quant.).

^{1}H NMR (500 MHz, CD_3CN, 298 K) δ 8.66 (s, 6H), 8.50 (s, 6H), 8.48–8.42 (m, 18H), 8.35 (td, *J* = 7.8, 1.5 Hz, 6H), 8.31 (d, *J* = 7.7 Hz, 6H), 8.24 (d, *J* = 7.8 Hz, 6H), 8.15 (d, *J* = 7.8 Hz, 6H), 8.00–7.95 (m, 12H), 7.91–7.85 (m, 12H), 7.79 (ddd, *J* = 7.8, 5.2, 1.2 Hz, 6H), 7.64 (d, 5.1 Hz, 6H), 7.56 (d, *J* = 9.0 Hz, 12H), 7.18 (d, *J* = 8.9 Hz, 12H), 7.14 (d, *J* = 8.8 Hz, 12H), 6.99 (d, *J* = 9.0 Hz, 12H), 6.82 (d, *J* = 8.8 Hz, 12H), 6.42 (d, *J* = 8.9 Hz, 12H), 6.26 (d, *J* = 9.0 Hz, 12H), 5.94 (d, *J* = 8.6 Hz, 12H).

^{13}C NMR (126 MHz, CD_3CN, 298 K) δ 166.5, 165.3, 163.5, 150.6, 150.2, 149.8, 147.9, 147.5, 147.1 (2 x ^{13}C), 147.0 (2 x ^{13}C), 146.7, 146.6, 144.2, 143.6, 143.5, 143.5, 143.2, 132.2, 132.0, 131.8, 131.6 (2 x ^{13}C), 131.5, 131.4, 130.3, 127.0, 126.4, 125.2, 124.3, 123.9, 123.9, 120.8 (q, *J* = 321 Hz, $^-NTf_2$), 120.3.

^{19}F NMR (376 MHz, CD_3CN, 298 K) δ −79.92.

ESI-HRMS ([**2**$(NTf_2)_{12}$] = $C_{252}H_{186}N_{44}Zn_6(C_2F_6NO_4S_2)_{12}$) *m/z* = 803.2481 [**2**$(NTf_2)_5$]$^{7+}$ (calc. 803.2494), 983.7777 [**2**$(NTf_2)_6$]$^{6+}$ (calc. 983.7770), 1236.7186 [**2**$(NTf_2)_7$]$^{5+}$ (calc. 1236.7161), 1615.8774 [**2**$(NTf_2)_8$]$^{4+}$ (calc. 1615.8743), 2247.8203 [**2**$(NTf_2)_9$]$^{3+}$ (calc. 2247.8053).

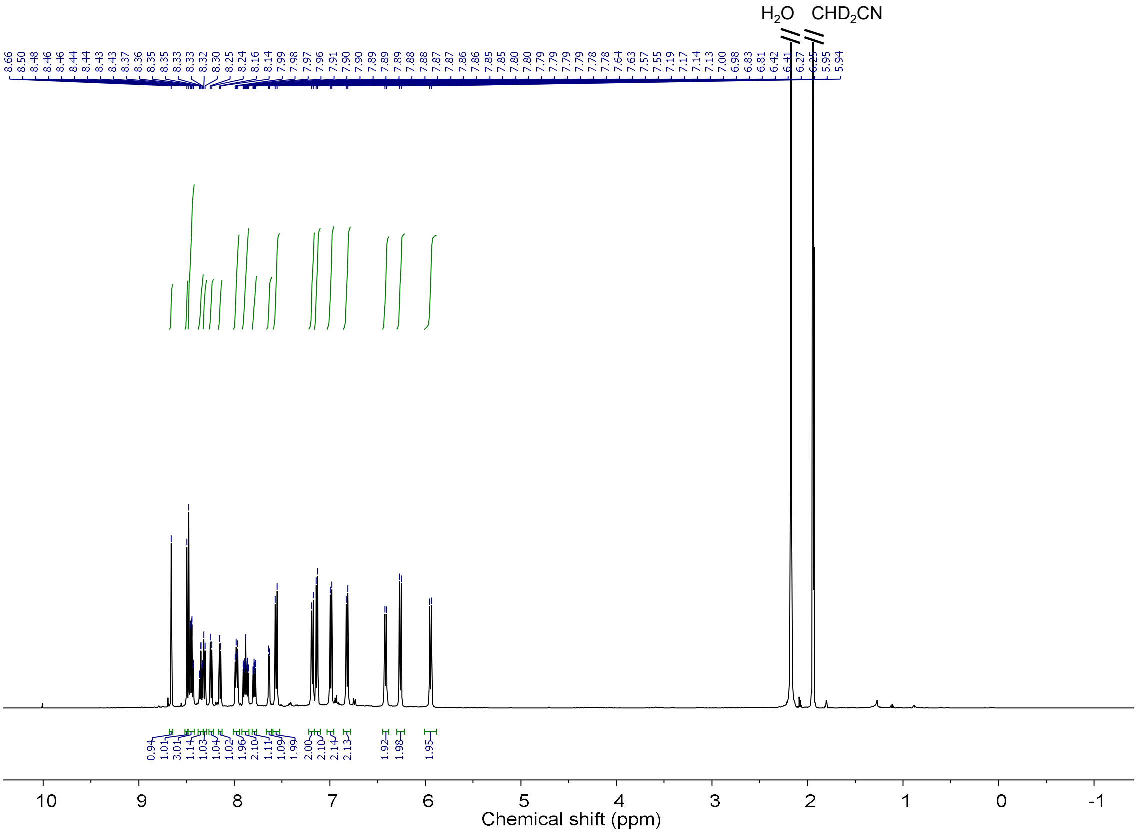

Figure S16. ^{1}H NMR spectrum (500 MHz, CD_3CN, 298 K) of **2**·$(NTf_2)_{12}$.

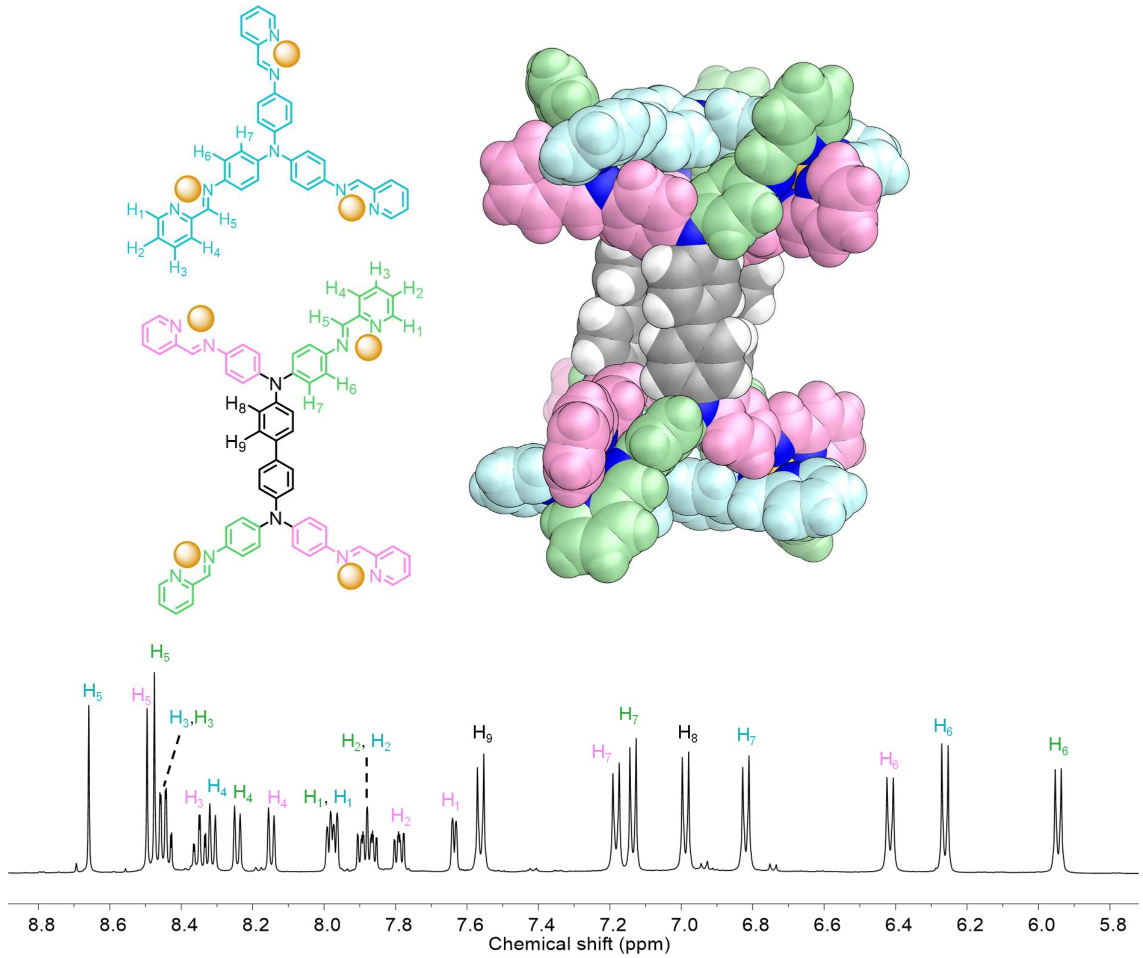

Figure S17. Aromatic region of the ^1H NMR spectrum (500 MHz, CD_3CN, 298 K) of **2**$\cdot(NTf_2)_{12}$, with assignment of signals. The assignment of the signals in the ^1H NMR spectrum to specific protons in structure **2** was carried out using a similar strategy to that outlined for **1** (Supporting Information Section 3.1).

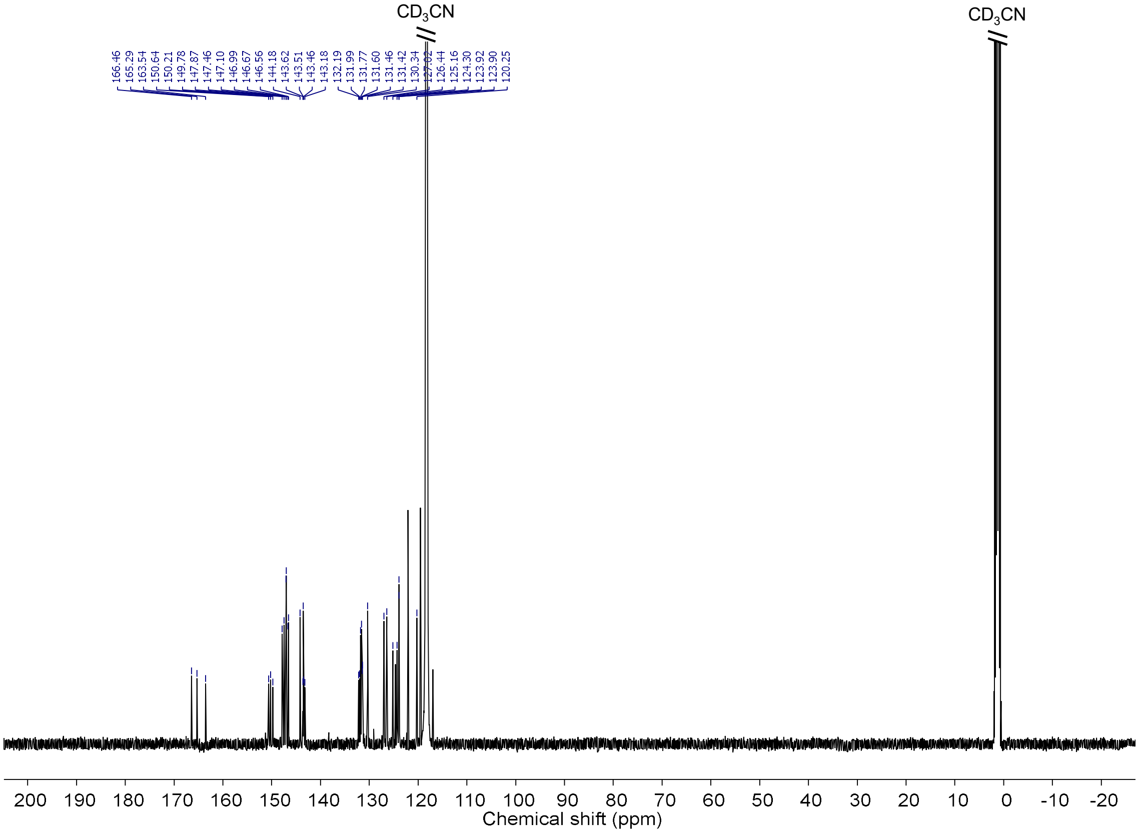

Figure S18. ^{13}C NMR spectrum (126 MHz, CD_3CN, 298 K) of **2**·$(NTf_2)_{12}$.

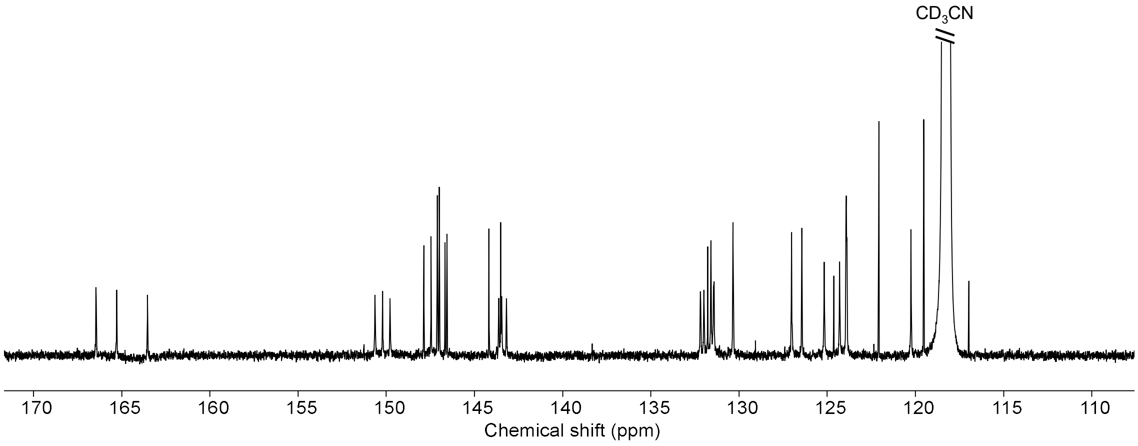

Figure S19. Aromatic region of the ^{13}C NMR spectrum (126 MHz, CD_3CN, 298 K) of **2**·$(NTf_2)_{12}$.

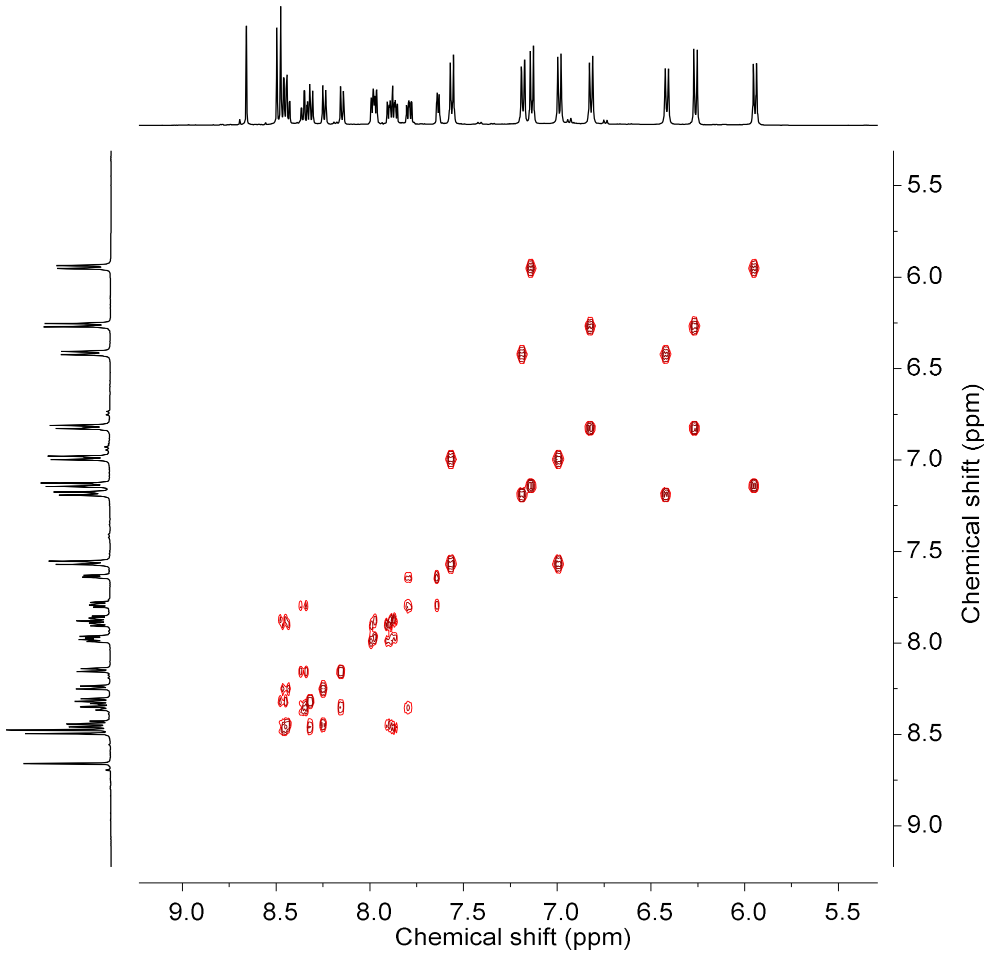

Figure S20. Aromatic region of the ^{1}H-^{1}H DQF-COSY spectrum (500 MHz, CD_3CN, 298 K) of **2**·$(NTf_2)_{12}$.

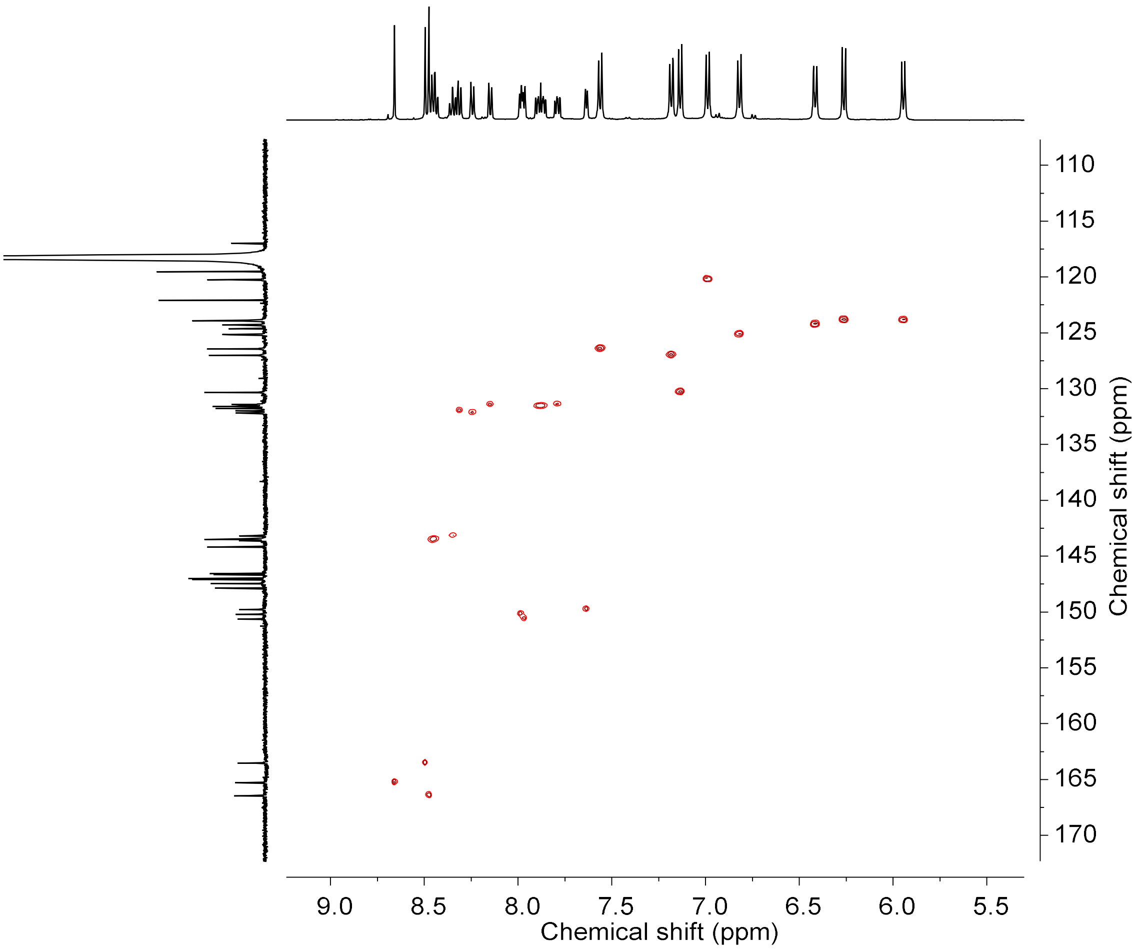

Figure S21. Aromatic region of the ^1H-^{13}C edited HSQC spectrum (500 MHz, CD_3CN, 298 K) of **2**·$(NTf_2)_{12}$.

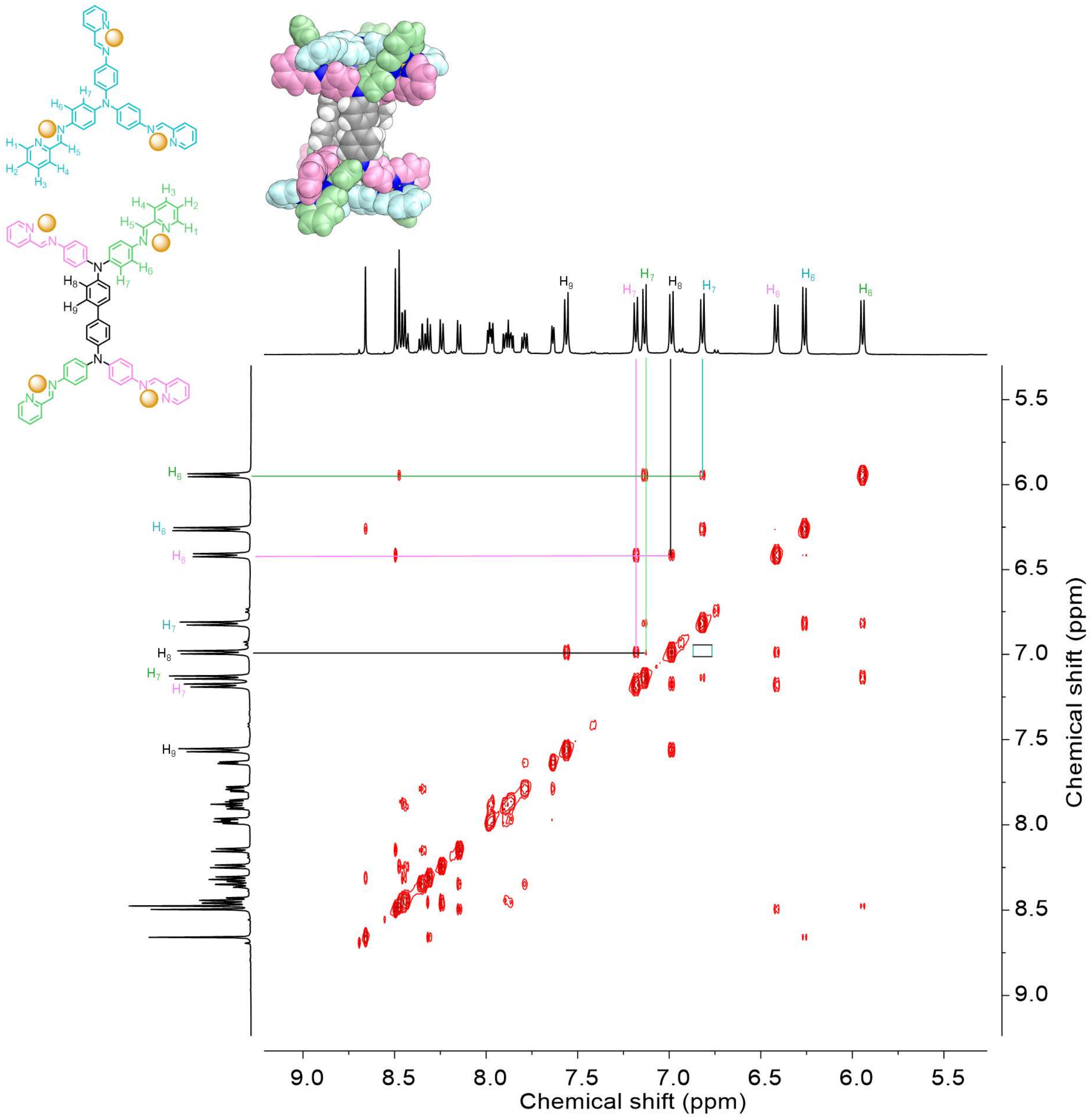

Figure S22. Aromatic region of the ^{1}H-^{1}H NOESY spectrum (500 MHz, CD_3CN, 298 K) of **2**$\cdot(NTf_2)_{12}$ with the presence/absence of key NOE correlations for the assignment of the three sets of signals in the ^{1}H NMR spectrum to specific ligand arms in **2** highlighted.

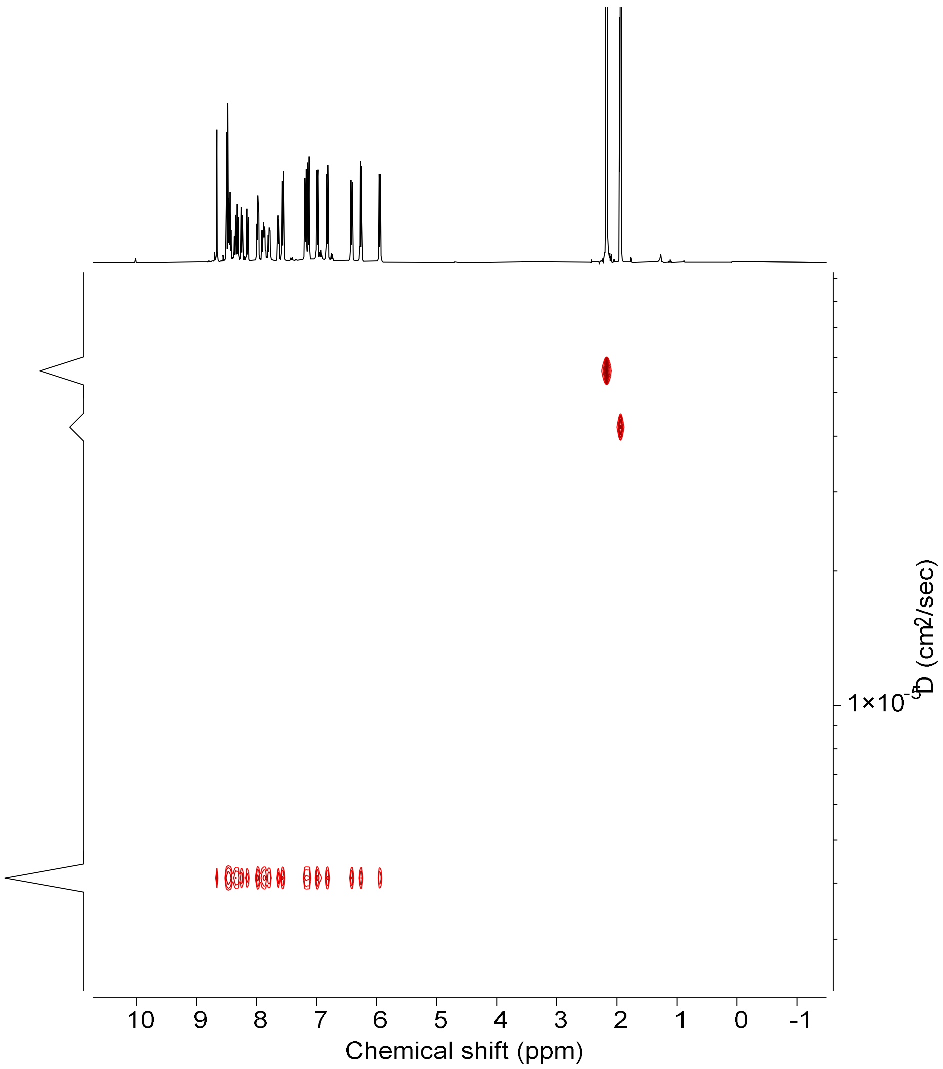

Figure S23. ^{1}H DOSY spectrum (400 MHz, CD_3CN, 298 K) of **2**·$(NTf_2)_{12}$.

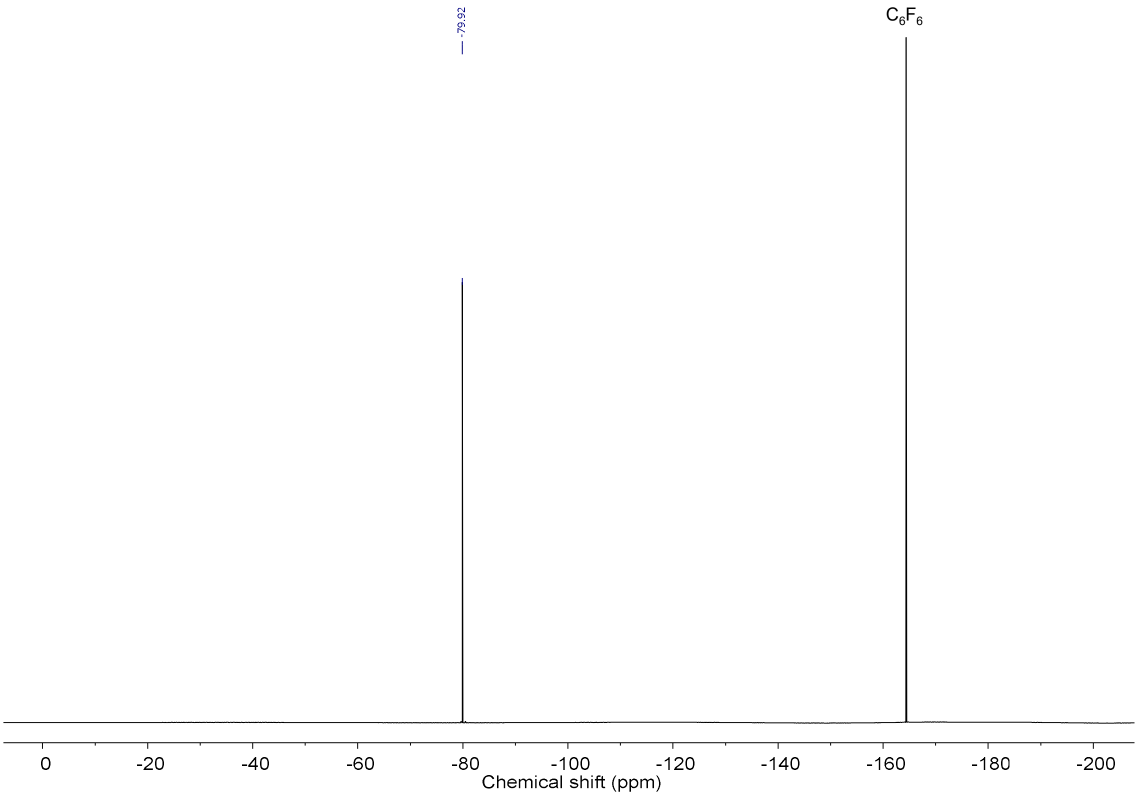

Figure S24. ^{19}F NMR spectrum (376 MHz, CD_3CN, 298 K) of **2**·$(NTf_2)_{12}$.

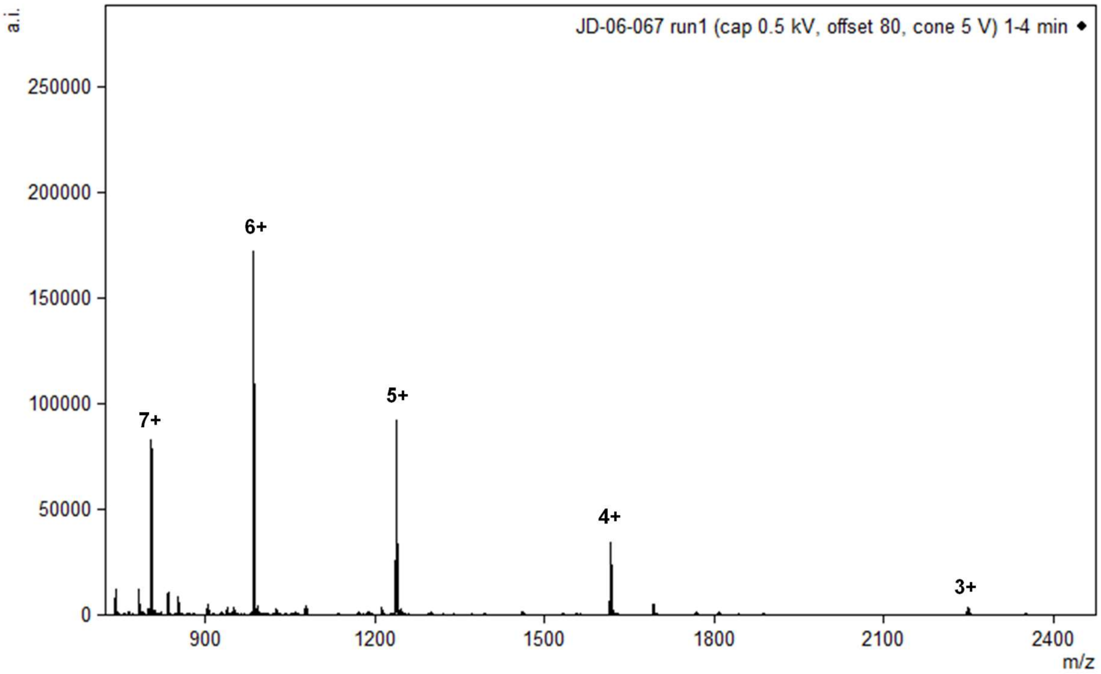

Figure S25. High resolution ESI-mass spectrum for **2**·$(NTf_2)_{12}$.

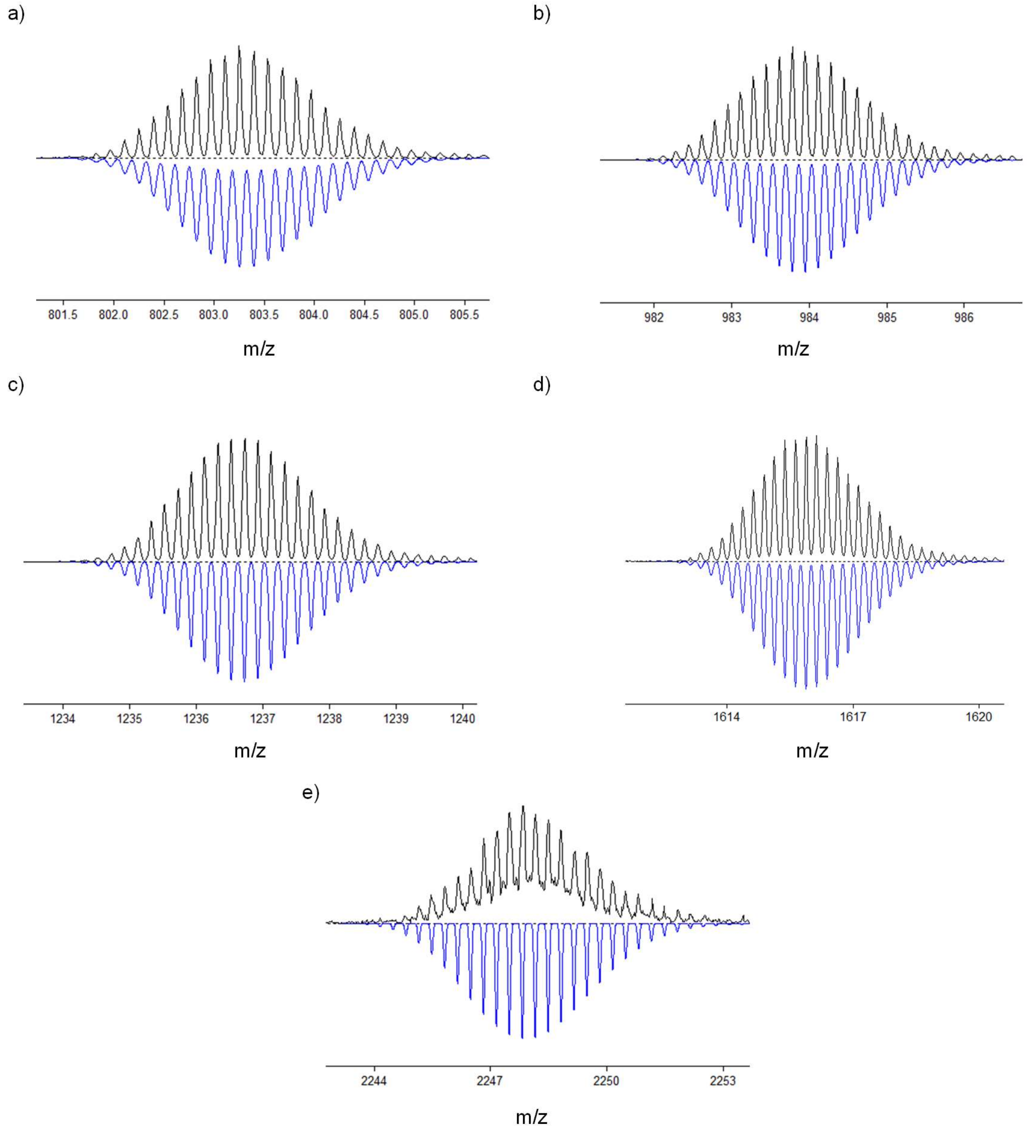

Figure S26. Signals from the high resolution ESI-mass spectrum for **2**·$(NTf_2)_{12}$. Experimental (black) and calculated (blue) signals for a) [**2**$(NTf_2)_5]^{7+}$ b) [**2**$(NTf_2)_6]^{6+}$ c) [**2**$(NTf_2)_7]^{5+}$ d) [**2**$(NTf_2)_8]^{4+}$ e) [**2**$(NTf_2)_9]^{3+}$.

3.3 Synthesis and characterization of 3

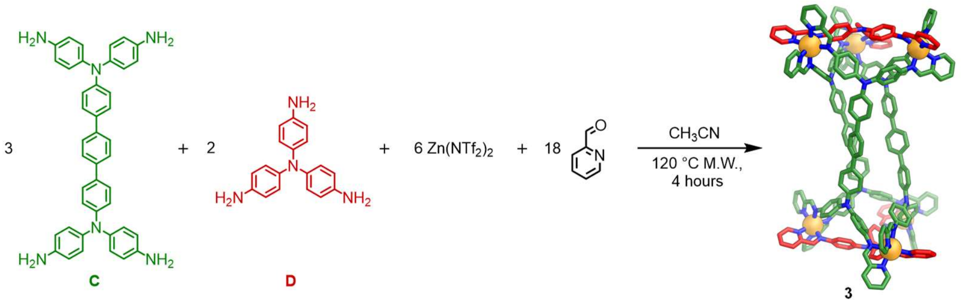

Scheme S4. Subcomponent self-assembly of **3**·$(NTf_2)_{12}$.

To a mixture of tetra-aniline **C** (7.1 mg, 11.4 μmol, 1.7 equiv), tri-aniline **D** (2.0 mg, 6.9 μmol, 1.0 equiv) and $Zn(NTf_2)_2$ (14.4 mg, 23.0 μmol, 3.3 equiv) in acetonitrile (4.0 mL), 2-formylpyridine (6.9 μL, 72.5 μmol, 10.5 equiv) was added. The reaction mixture was heated at 120 °C for 4 hours in a microwave reactor. The reaction mixture was allowed to cool to room temperature, filtered through a glass fibre plug, concentrated to approx. 1 mL and diethyl ether (8 mL) was added. The solid was washed with diethyl ether (3 × 8 mL) and the product **3**$\cdot(NTf_2)_{12}$ was obtained as a very dark red/orange solid (27.4 mg, quant.).

^{1}H NMR (500 MHz, CD_3CN, 298 K) δ 8.65 (s, 6H), 8.52 (s, 6H), 8.49–8.42 (m, 18H), 8.37 (td, *J* = 7.8, 1.5 Hz, 6H), 8.31 (d, *J* = 7.8 Hz, 6H), 8.24 (d, *J* = 7.8 Hz, 6H), 8.15 (d, *J* = 7.8 Hz, 6H), 7.98 (d, *J* = 5.1 Hz, 6H), 7.93 (d, *J* = 5.1 Hz, 6 H), 7.90–7.86 (m, 12H), 7.81 (ddd, *J* = 7.8, 5.1, 1.1 Hz, 6H), 7.69 (d, *J* = 5.1 Hz, 6H), 7.51 (d, *J* = 8.8 Hz, 12H), 7.34 (s, 12H), 7.20–7.14 (m, 24H), 7.07 (d, *J* = 8.8 Hz, 12H), 6.87 (d, *J* = 8.9 Hz, 12H), 6.44 (d, *J* = 8.8 Hz, 12H), 6.30 (d, *J* = 8.9 Hz, 12H), 6.03 (d, *J* = 8.7 Hz, 12H).

^{13}C NMR (126 MHz, CD_3CN, 298 K) δ 166.2, 165.4, 163.9, 150.6, 150.2, 149.9, 148.0, 147.4, 147.3, 147.2, 147.1, 147.0, 146.9, 146.2, 144.4, 143.6, 143.5, 143.5, 143.3, 138.8, 134.4, 132.1, 132.0, 131.6, 131.6, 131.5, 131.5, 129.4, 128.1, 127.3, 126.1, 125.4, 124.3, 124.0 (2 x ^{13}C), 121.7, 120.8 (q, *J* = 321 Hz, $^-NTf_2$).

^{19}F NMR (376 MHz, CD_3CN, 298 K) δ −79.95.

ESI-HRMS ([**3**$(NTf_2)_{12}$] = $C_{270}H_{198}N_{44}Zn_6(C_2F_6NO_4S_2)_{12}$) *m/z* = 696.3642 [**3**$(NTf_2)_4]^{8+}$ (calc. 696.3653), 835.9767 [**3**$(NTf_2)_5]^{7+}$ (calc. 835.9771), 1021.7946 [**3**$(NTf_2)_6]^{6+}$ (calc. 1021.7928), 1282.3379 [**3**$(NTf_2)_7]^{5+}$ (calc. 1282.3351), 1672.9034 [**3**$(NTf_2)_8]^{4+}$ (calc. 1672.8980).

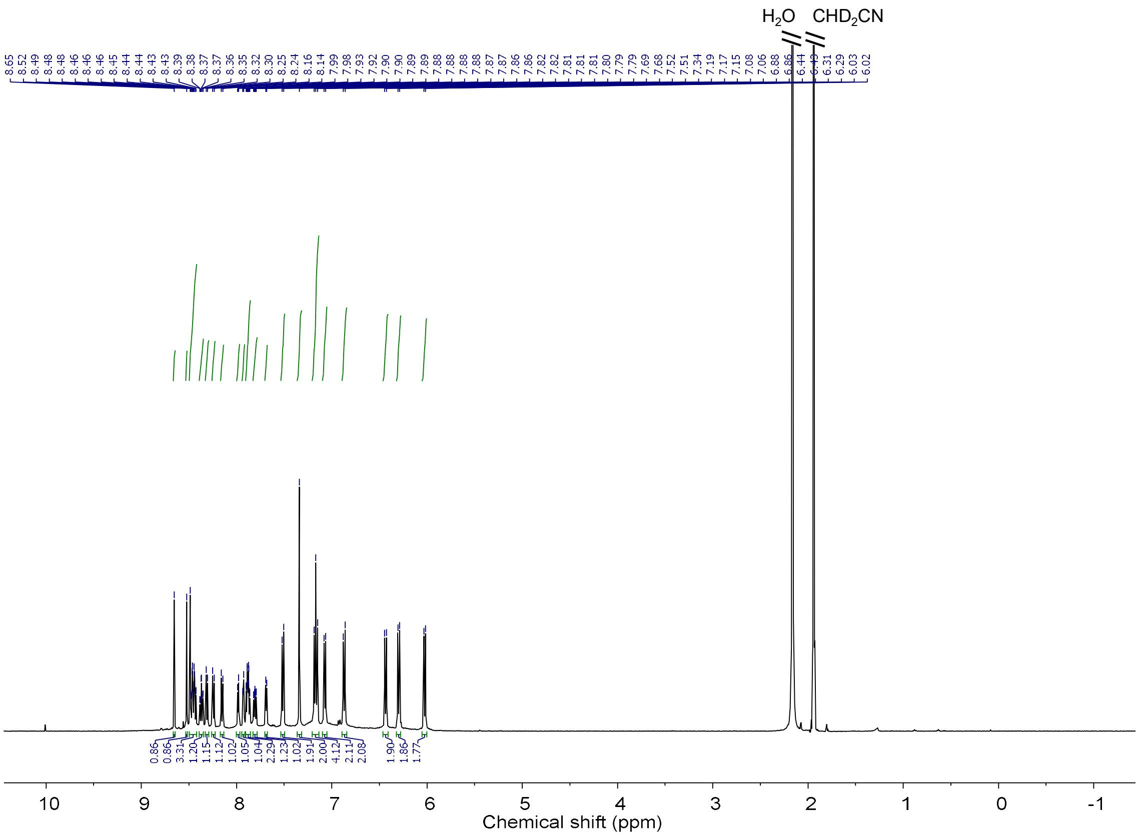

Figure S27. ^{1}H NMR spectrum (500 MHz, CD_3CN, 298 K) of **3**·$(NTf_2)_{12}$.

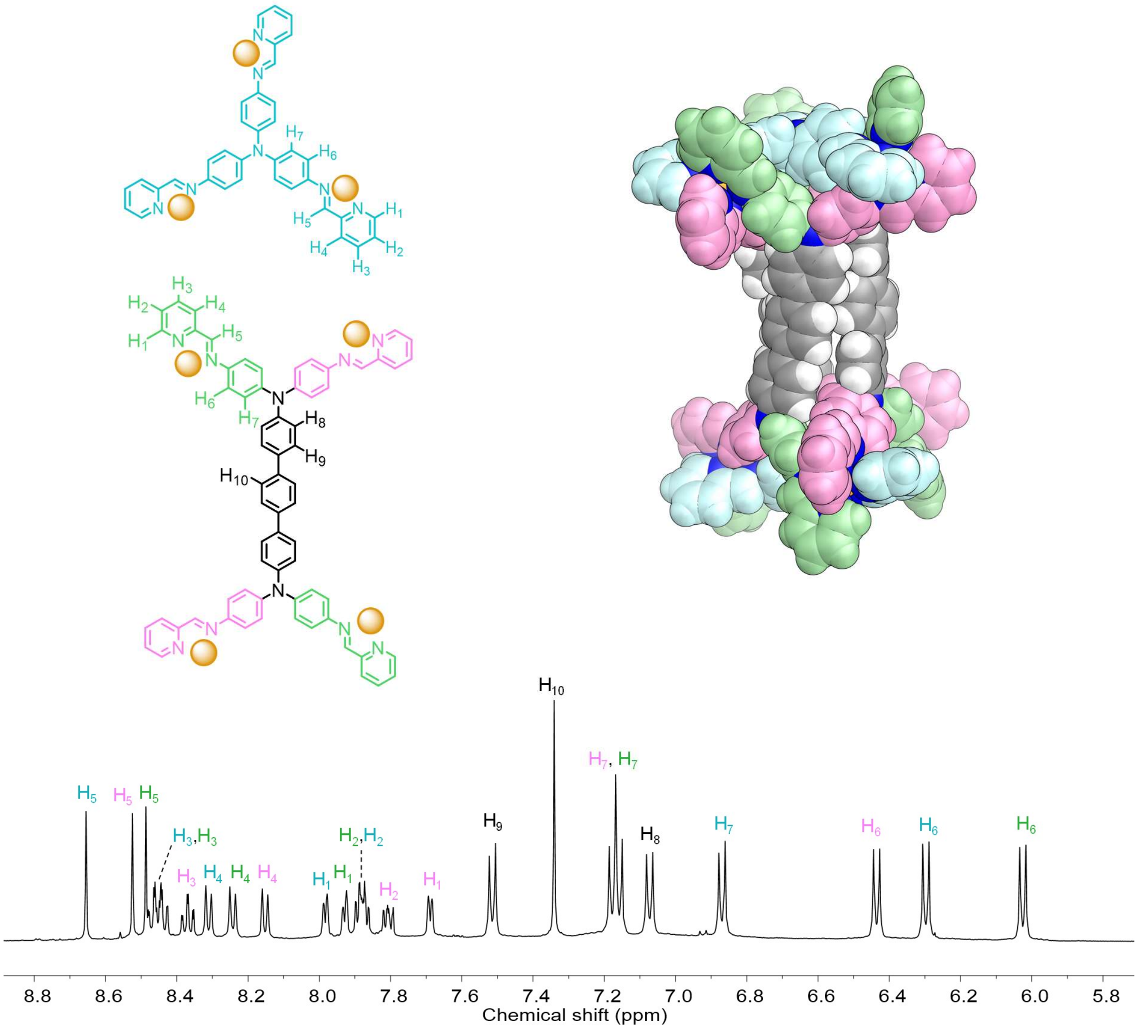

Figure S28. Aromatic region of the ^{1}H NMR spectrum (500 MHz, CD_3CN, 298 K) of **3**$\cdot(NTf_2)_{12}$, with assignment of signals. The assignment of the ^{1}H NMR signals to specific protons in structure **3** was carried out using a similar strategy to that outlined for **1** (Supporting Information Section 3.1). To distinguish between H_1 and H_1, the ^{1}H-^{1}H TOCSY spectrum was used (Figure S33).

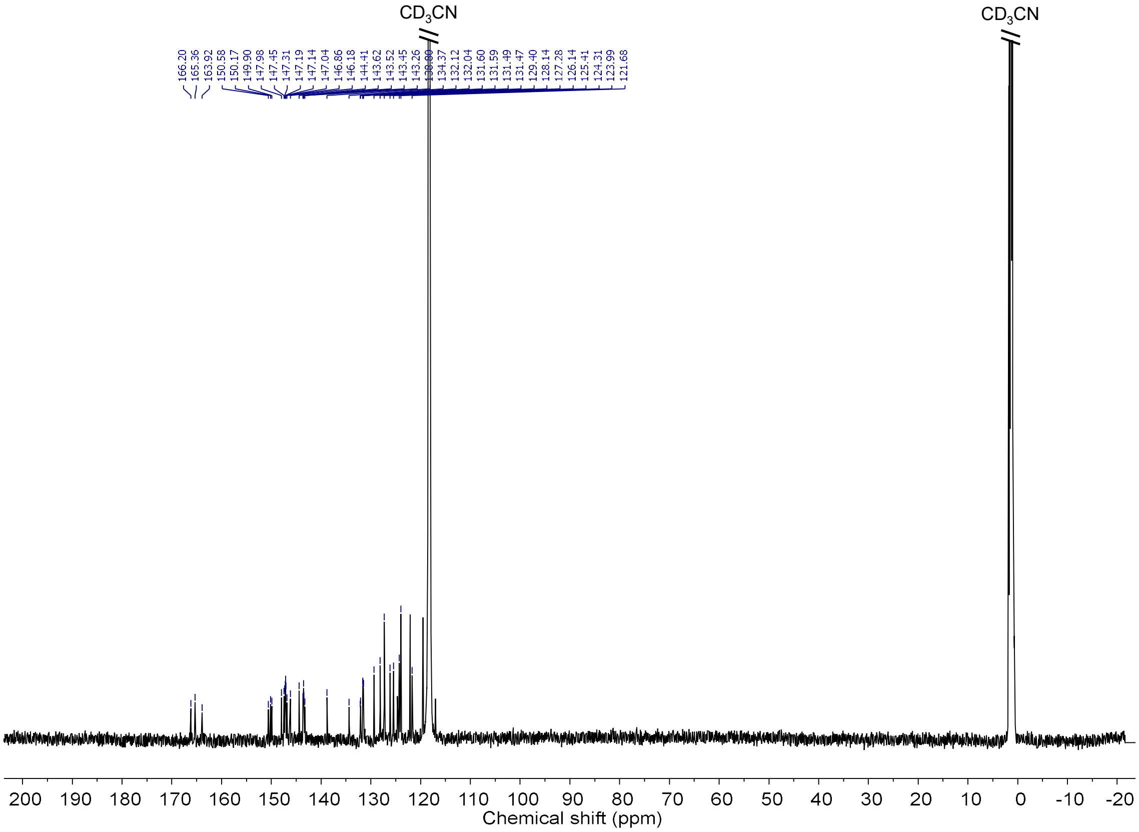

Figure S29. ^{13}C NMR spectrum (126 MHz, CD_3CN, 298 K) of **3**·$(NTf_2)_{12}$.

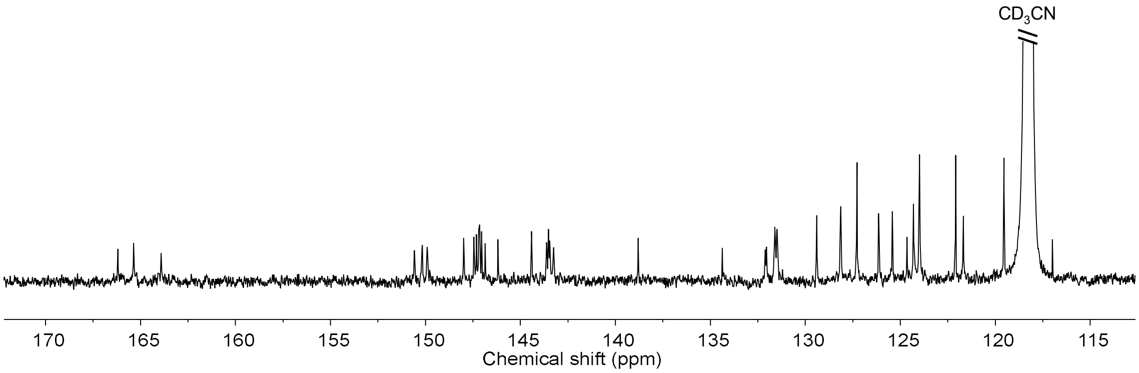

Figure S30. Aromatic region of the ^{13}C NMR spectrum (126 MHz, CD_3CN, 298 K) of **3**·$(NTf_2)_{12}$.

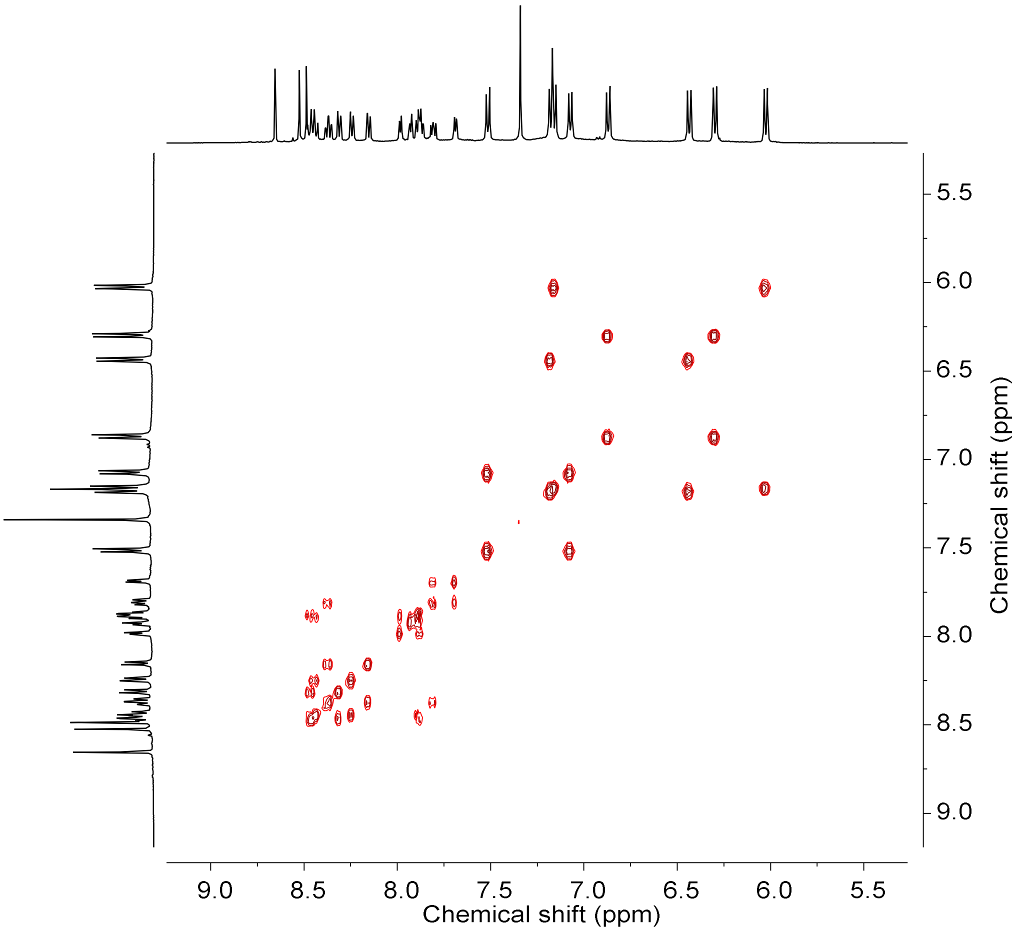

Figure S31. Aromatic region of the ^1H-^1H DQF-COSY spectrum (500 MHz, CD_3CN, 298 K) of **3**·$(NTf_2)_{12}$.

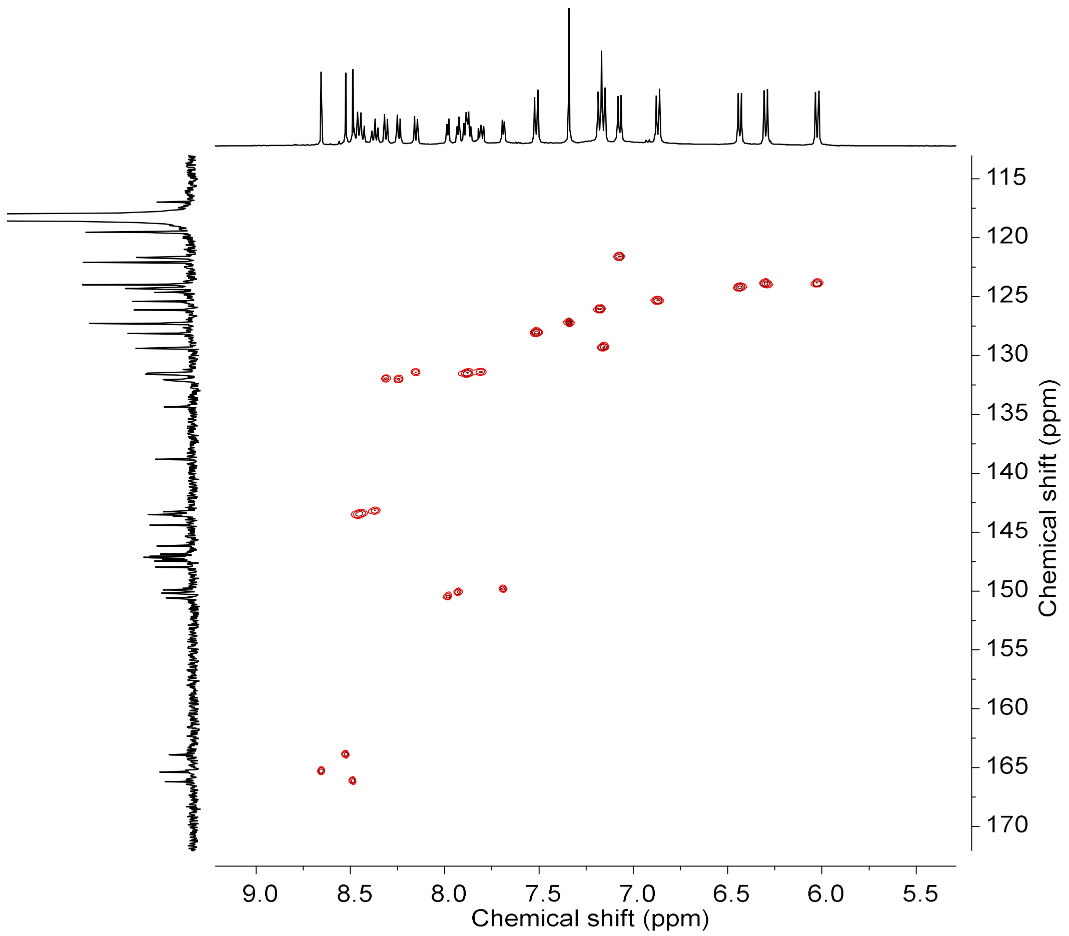

Figure S32. Aromatic region of the ^{1}H-^{13}C edited HSQC spectrum (500 MHz, CD_3CN, 298 K) of **3**·$(NTf_2)_{12}$.

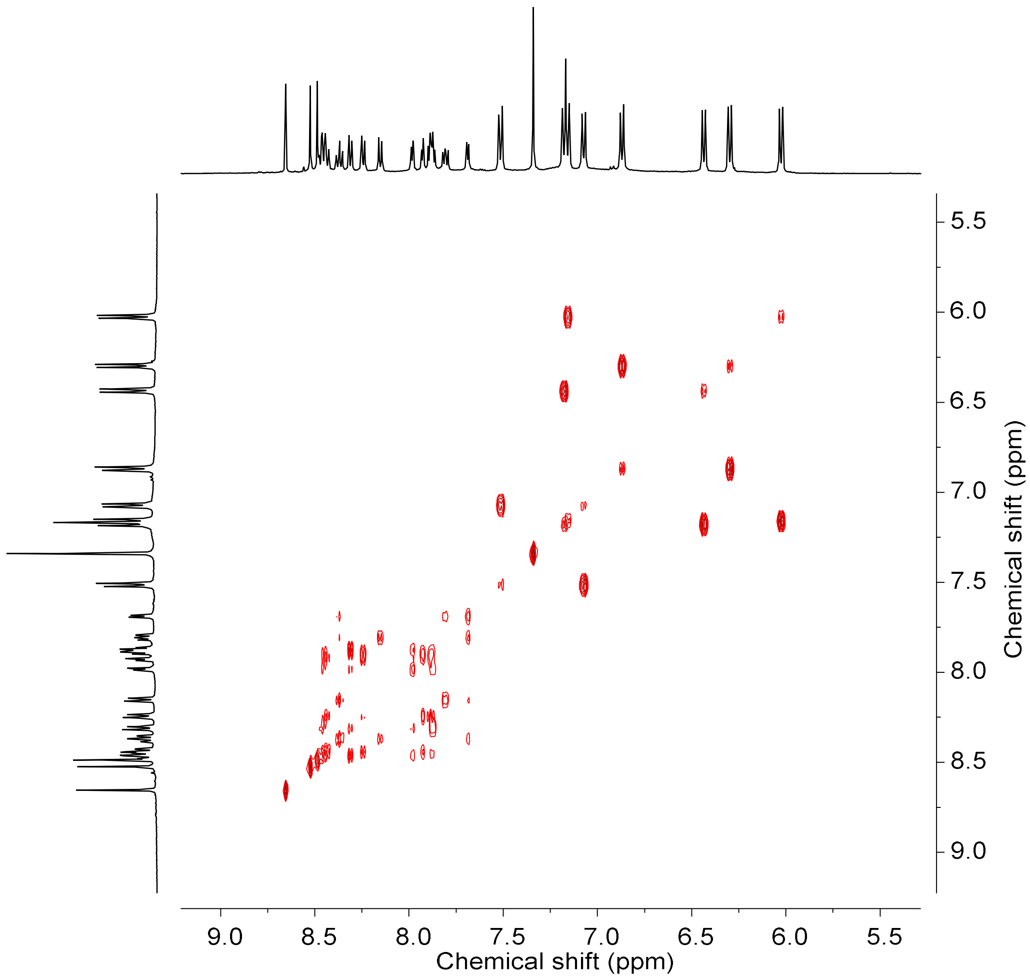

Figure S33. Aromatic region of the ^{1}H-^{1}H TOCSY spectrum (500 MHz, CD_3CN, 298 K) of **3**·$(NTf_2)_{12}$.

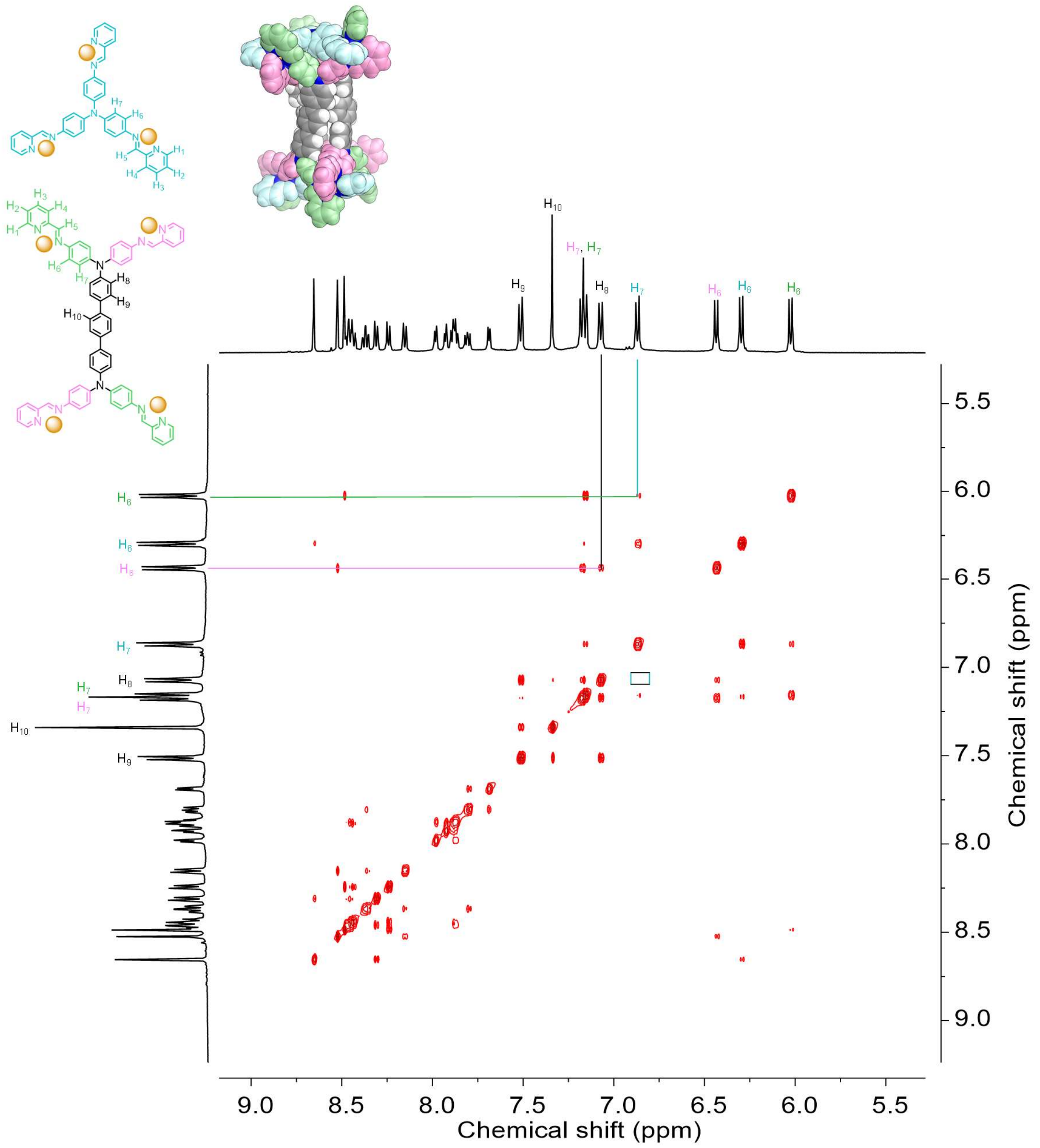

Figure S34. Aromatic region of the ^1H-^1H NOESY spectrum (500 MHz, CD_3CN, 298 K) of **3**·$(NTf_2)_{12}$ with the presence/absence of key NOE correlations for the assignment of the three sets of signals in the ^1H NMR spectrum to specific ligand arms in **3** highlighted.

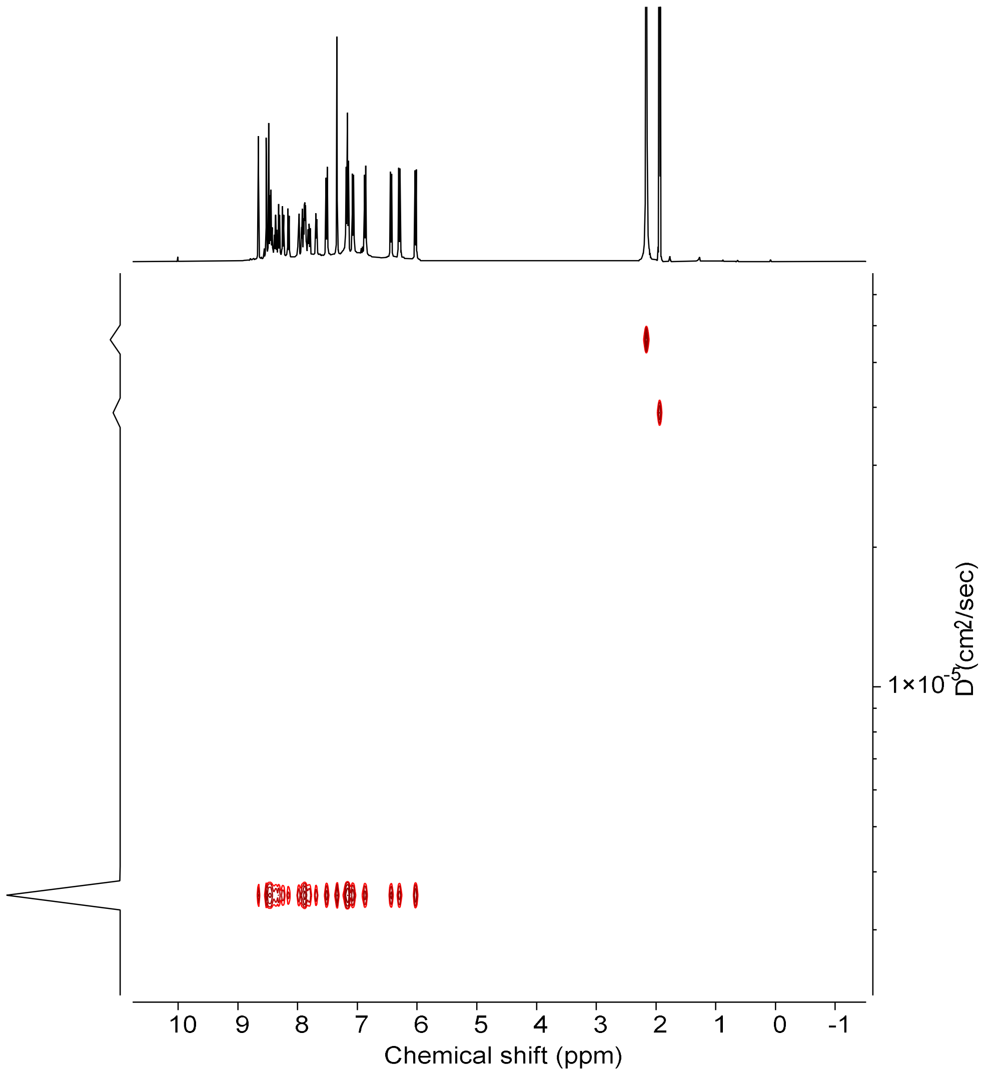

Figure S35. ^{1}H DOSY spectrum (400 MHz, CD_3CN, 298 K) of **3**·$(NTf_2)_{12}$.

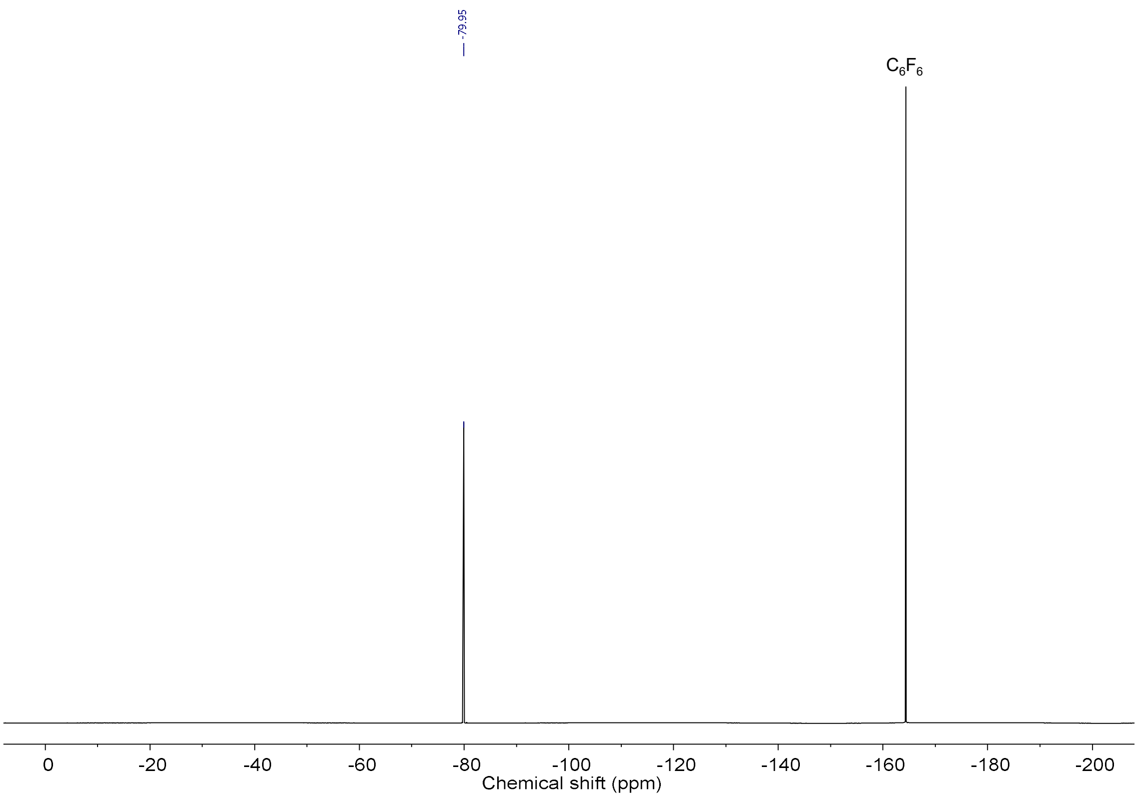

Figure S36. ^{19}F NMR spectrum (376 MHz, CD_3CN, 298 K) of **3**·$(NTf_2)_{12}$.

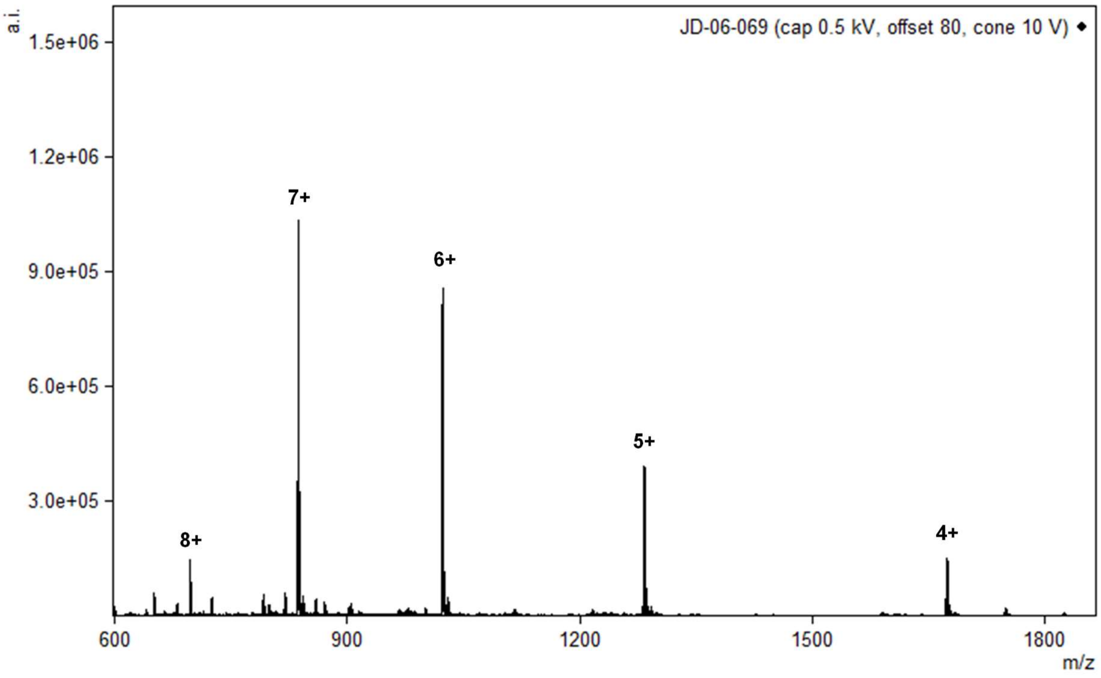

Figure S37. High resolution ESI-mass spectrum for **3**·$(NTf_2)_{12}$.

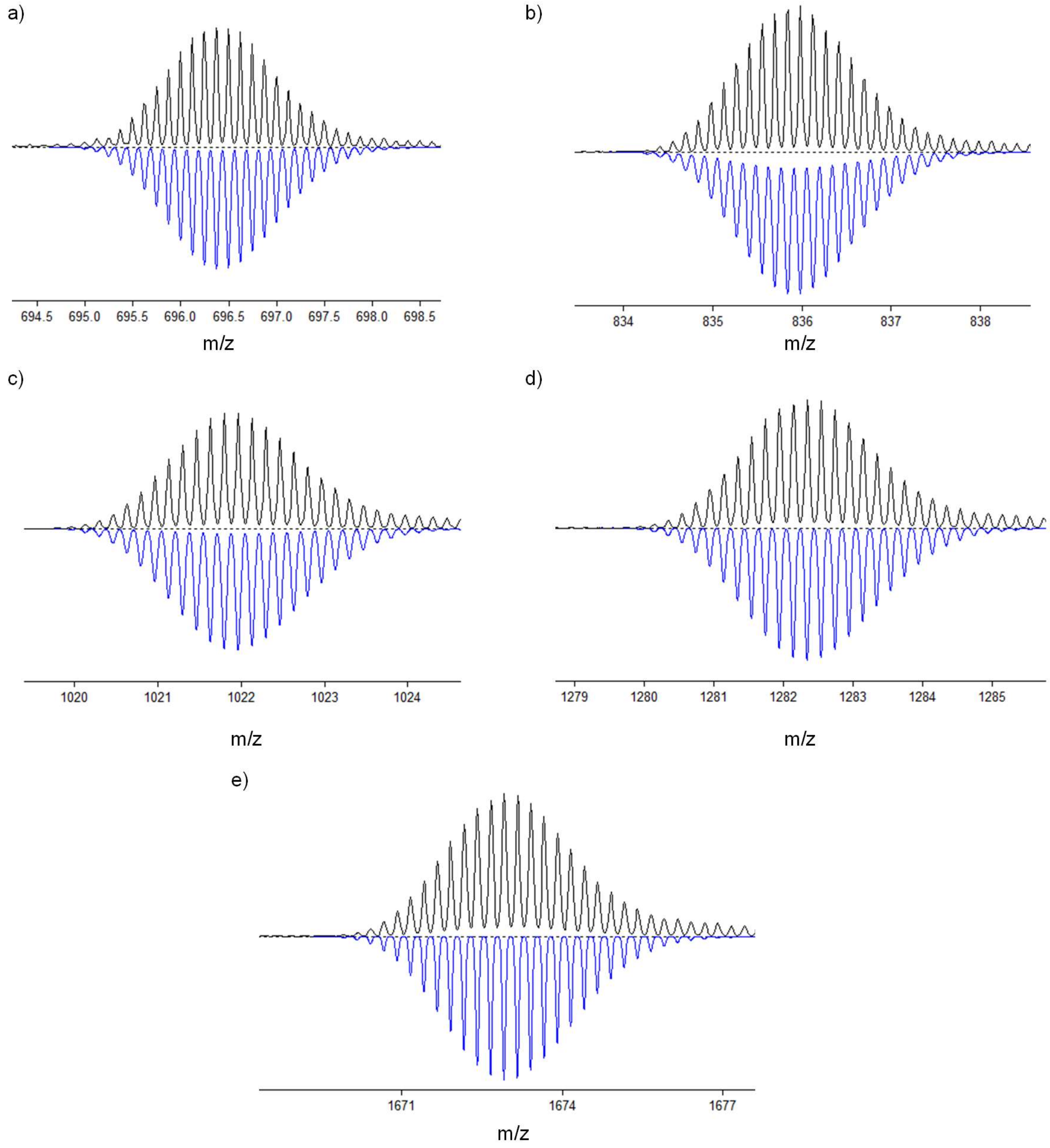

Figure S38. Signals from the high resolution ESI-mass spectrum for **3**·$(NTf_2)_{12}$. Experimental (black) and calculated (blue) signals for a) [**3**$(NTf_2)_4]^{8+}$ b) [**3**$(NTf_2)_5]^{7+}$ c) [**3**$(NTf_2)_6]^{6+}$ d) [**3**$(NTf_2)_7]^{5+}$ e) [**3**$(NTf_2)_8]^{4+}$.

3.4 Synthesis and characterization of 4

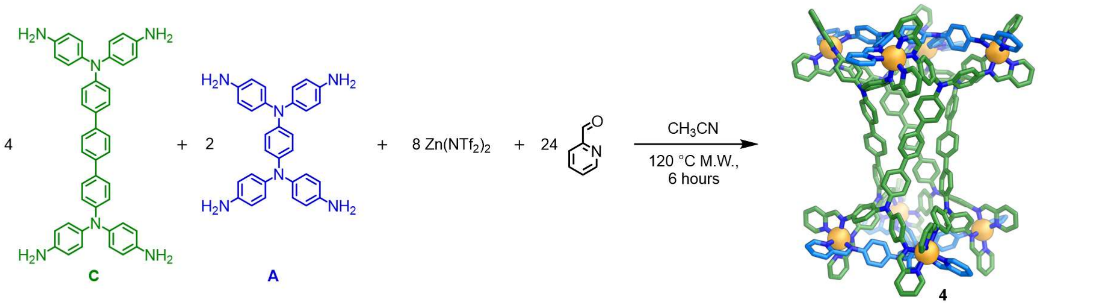

Scheme S5. Subcomponent self-assembly of **4**·$(NTf_2)_{16}$.

To a mixture of tetra-aniline **A** (1.0 mg, 2.1 μmol, 1.0 equiv), tetra-aniline **C** (2.6 mg, 4.2 μmol, 2.0 equiv) and $Zn(NTf_2)_2$ (6.0 mg, 9.6 μmol, 4.6 equiv) in acetonitrile (2.4 mL), 2-formylpyridine (2. 6 μL, 27.3 μmol, 13.0 equiv) was added. The reaction mixture was heated at 120 °C for 6 hours in a microwave reactor. The reaction mixture was allowed to cool to room temperature, filtered through a glass fibre plug and concentrated to a small volume. Diethyl ether (8 mL) was added and the solid was washed with diethyl ether (3 × 8 mL). The product **4**·$(NTf_2)_{16}$ was obtained as a dark red/orange solid (11.5 mg, quant.).

^{1}H NMR (500 MHz, CD_3CN, 298 K) δ 8.77 (s, 4H), 8.76 (s, 4H), 8.62 (s, 4H), 8.58 (s, 4H), 8.58 (s, 4H), 8.47 (td, *J* = 7.7, 1.5 Hz, 4H), 8.44–8.38 (m, 12H), 8.36–8.30 (m, 12H*), 8.28–8.20 (m, 16H), 8.16 (s, 4H), 8.11 (d, *J* = 7.8 Hz, 4H), 8.08–8.05 (m, 8H), 7.93–7.87 (m, 12H*), 7.84–7.79 (m, 8H), 7.78–7.72 (m, 8H), 7.71 (d, *J* = 5.1 Hz, 4H), 7.54 (d, *J* = 4.7 Hz, 4H), 7.48 (d, *J* = 8.5 Hz, 8H), 7.41 (d, *J* = 5.0 Hz, 4H), 7.35–7.26 (m, 24H), 7.25–7.19 (m, 16H), 7.14 (d, *J* = 8.9 Hz, 8H), 7.12–7.06 (m, 24H), 7.01 (d, *J* = 8.8 Hz, 8H), 6.85 (d, *J* = 8.6 Hz, 8H), 6.69 (s, 8H), 6.55 (d, *J* = 9.0 Hz, 8H), 6.53 (d, *J* = 8.9 Hz, 8H), 6.44 (d, *J* = 8.8 Hz, 8H), 6.32 (d, *J* = 8.8 Hz, 8H), 6.15 (d, *J* = 8.7 Hz, 8H), 5.86 (d, *J* = 8.8 Hz, 8H). Only the signals attributed to the 'major' species are listed. *The relative integrals for these signals in the ^{1}H NMR spectrum were slightly higher than expected (and quoted) due to signal overlap, and potentially due to the presence of the signals for the additional 'minor' species which also overlap with the signals for the 'major' species.

^{13}C NMR (126 MHz, CD_3CN, 298 K) δ 165.3, 165.1, 165.1, 164.9, 163.1, 162.6, 150.9, 150.5, 150.4, 149.9, 149.9, 149.6, 148.5, 148.3, 148.2, 148.1, 147.5, 147.4, 147.4, 147.3, 147.1, 147.0, 146.9, 146.9, 146.7, 146.7, 145.9, 145.6, 143.8, 143.7, 143.5, 143.5, 143.4, 143.2, 143.1, 142.8, 142.2, 142.1, 140.1, 139.8, 136.5, 136.2, 132.4, 132.3, 132.2, 131.9, 131.7, 131.6, 131.5, 131.4, 131.4, 131.2, 130.2, 128.9, 128.6, 128.2, 128.1, 126.0, 125.9, 125.4, 124.8, 124.6 (overlapping with the furthest left peak of the quartet for -**C**F_3 in $^-NTf_2$), 124.5, 124.2, 124.1, 124.0, 123.4, 121.8,120.8 (q, *J* = 321 Hz, $^-NTf_2$), 117.4. The identification of slightly fewer signals than the expected number of distinct ^{13}C environments was attributed to signal overlap and reduced signal intensity, arising from the desymmetrisation of the ligand in the cage structure.

^{19}F NMR (376 MHz, CD_3CN, 298 K) δ −79.91.

ESI-HRMS ([**4**$(NTf_2)_{16}$] = $C_{372}H_{272}N_{60}Zn_8(C_2F_6NO_4S_2)_{16}$) *m/z* = 896.2430 [**4**$(NTf_2)_7$]$^{9+}$ (calc. 896.2398), 1043.2634 [**4**$(NTf_2)_8$]$^{8+}$ (calc. 1043.2597), 1232.4327 [**4**$(NTf_2)_9$]$^{7+}$ (calc. 1232.4278), 1484.3251

[**4**$(NTf_2)_{10}]^{6+}$ (calc. 1484.3189), 1837.1761 [**4**$(NTf_2)_{11}]^{5+}$ (calc. 1837.1662), 2366.7058 [**4**$(NTf_2)_{12}]^{4+}$ (calc. 2366.6866).

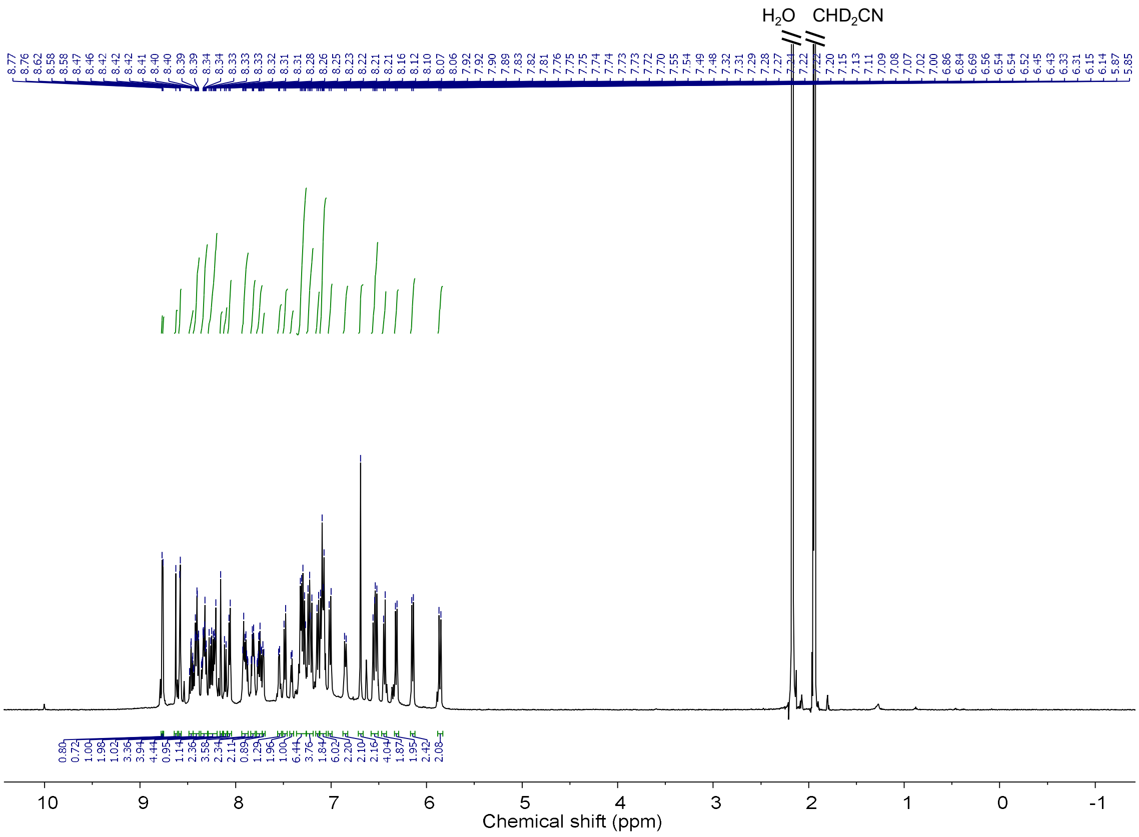

Figure S39. ^{1}H NMR spectrum (500 MHz, CD_3CN, 298 K) of **4**·$(NTf_2)_{16}$.

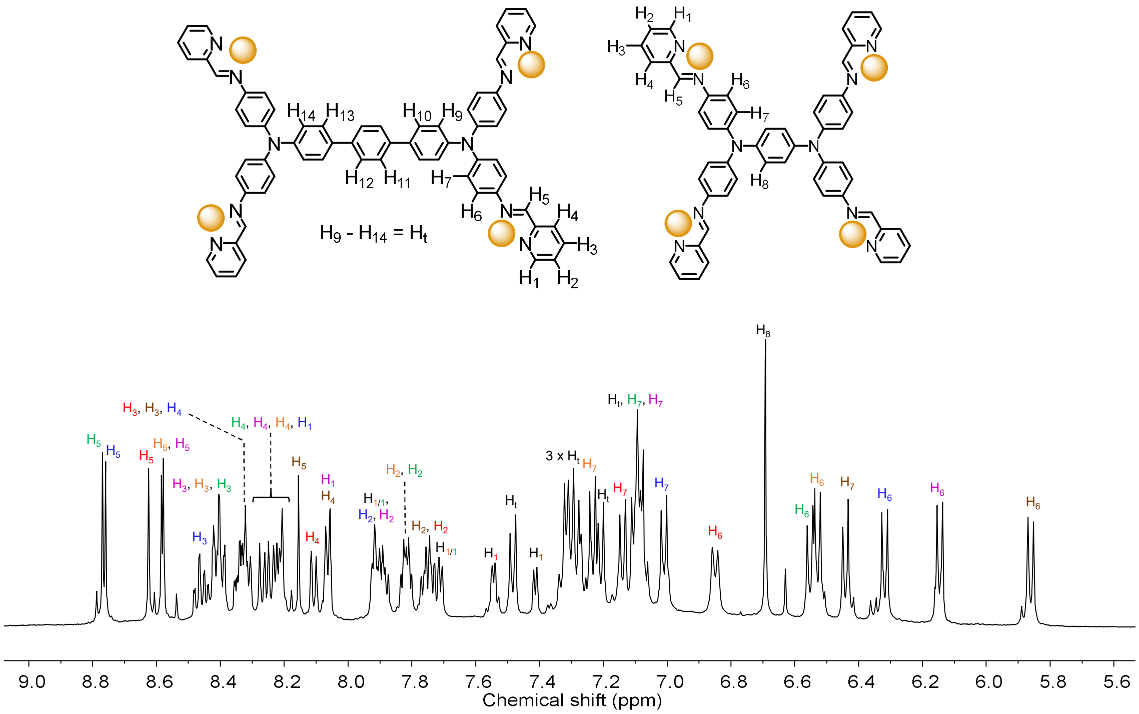

Figure S40. Aromatic region of the ^1H NMR spectrum (500 MHz, CD_3CN, 298 K) of **4**·$(NTf_2)_{16}$, with assignment of signals for the major species. The ^1H-^{13}C edited HSQC spectrum (Figure S44) enabled identification of six imine (H_5) signals. The ^1H-^1H NOESY spectrum (Figure S46) then allowed identification of the signals for six H_4 and H_6 proton environments. The ^1H-^1H DQF-COSY spectrum (Figure S43) enabled complete identification of the six sets of signals for the proton environments labelled H_1–H_7 on the six unique ligand arms (shown with different coloured labels above). The ^1H-^1H TOCSY spectrum (Figure S47) was used to distinguish between H_1 and H_1. The signals for H_8 and H_t (H_9–H_{14}) were identified using a combination of ^1H–^1H DQF-COSY (Figure S43) and ^1H-^1H NOESY (Figure S46) spectra , and relative integrals of signals in the ^1H NMR spectrum (Figure S39). It remained challenging to conclusively assign each of the six sets of signals to a specific ligand arm in the structure, as well as to assign signals H_9–H_{14} to specific proton environments. Both were due to a lack of characteristic cross peaks in the ^1H-^1H NOESY spectrum of **4**·$(NTf_2)_{16}$, because of overlapping signals in the ^1H NMR spectrum. The signals for the minor species could not be assigned due to their relatively low intensity and the overlap of signals.

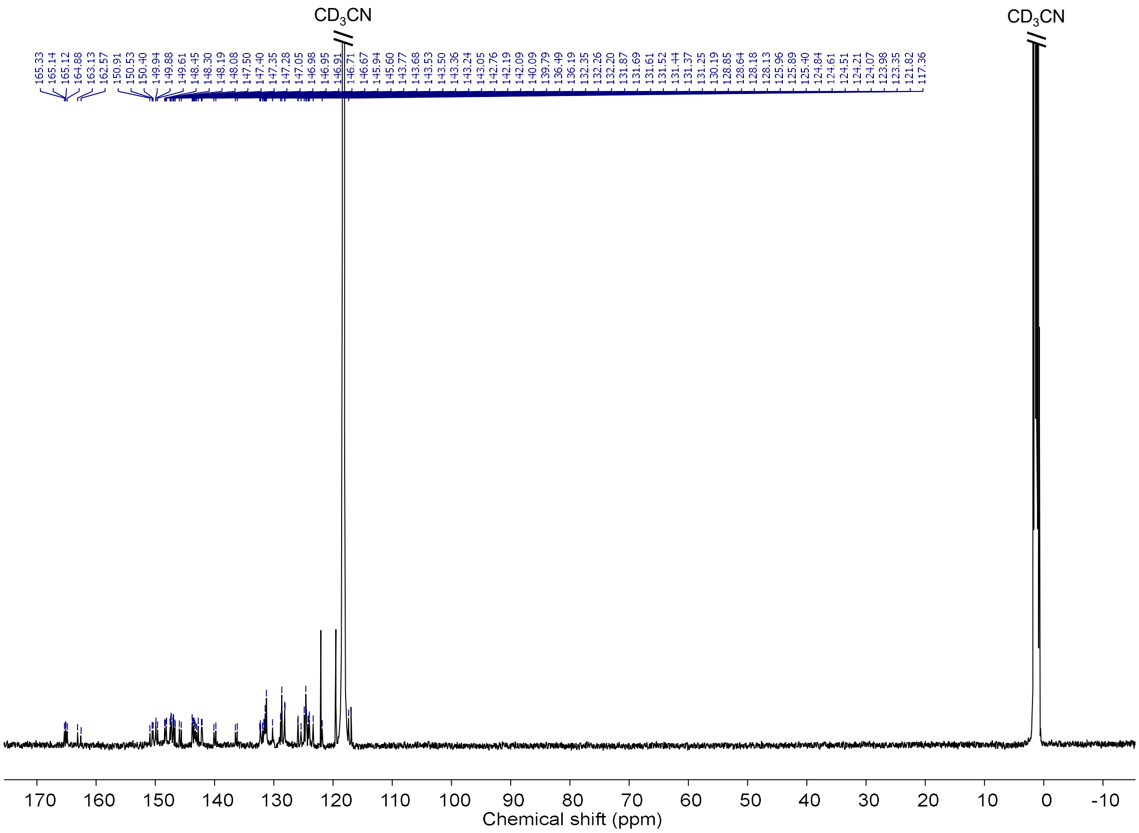

Figure S41. ^{13}C NMR spectrum (126 MHz, CD_3CN, 298 K) of **4**·$(NTf_2)_{16}$.

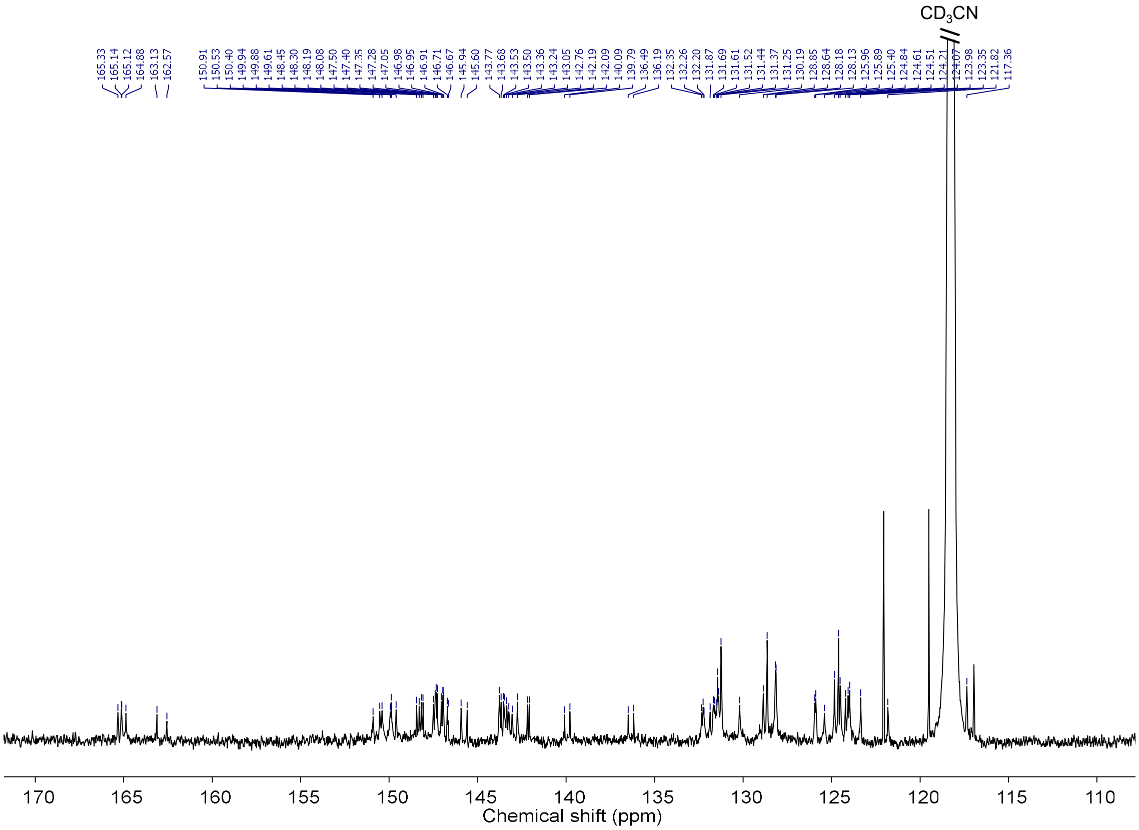

Figure S42. Aromatic region of the ^{13}C NMR spectrum (126 MHz, CD_3CN, 298 K) of **4**·$(NTf_2)_{16}$.

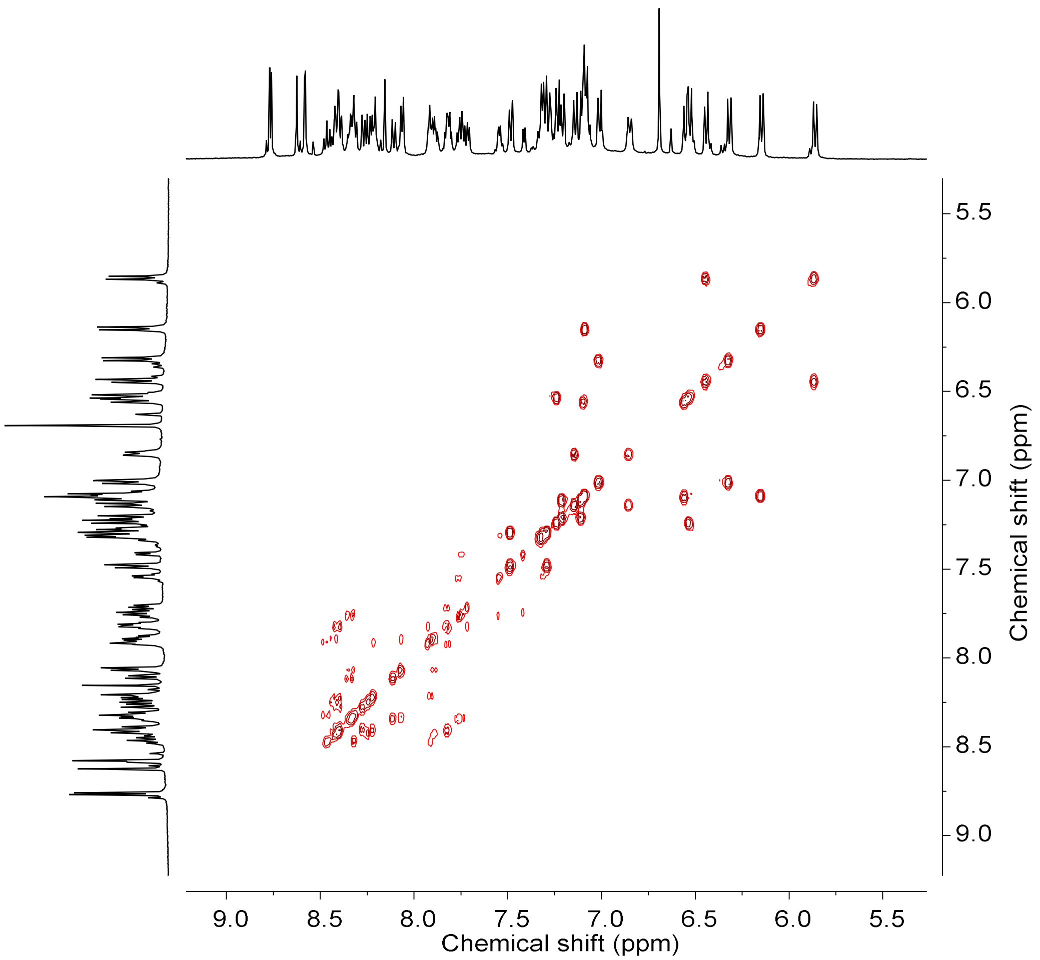

Figure S43. Aromatic region of the ^1H-^1H DQF-COSY spectrum (500 MHz, CD_3CN, 298 K) of **4**·$(NTf_2)_{16}$.

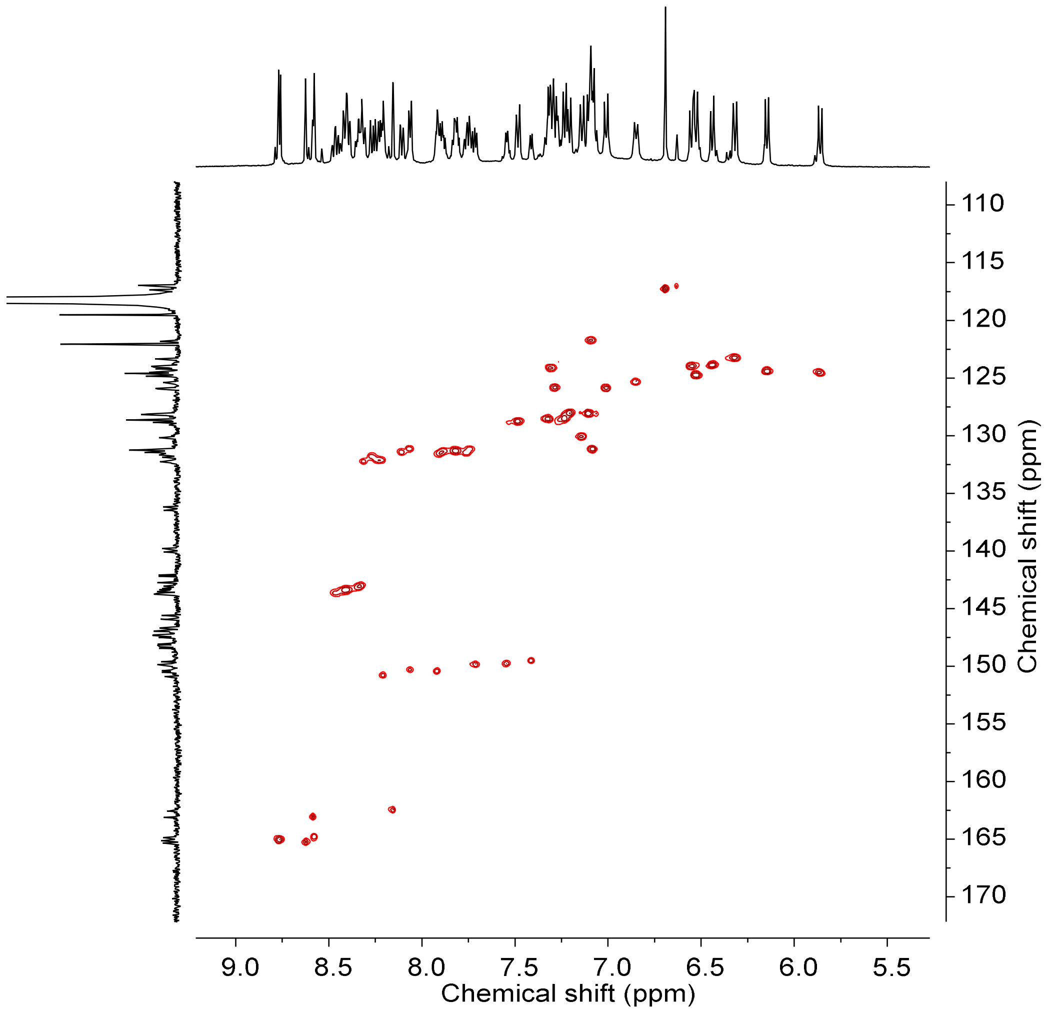

Figure S44. Aromatic region of the ^{1}H-^{13}C edited HSQC spectrum (500 MHz, CD_3CN, 298 K) of **4**·$(NTf_2)_{16}$.

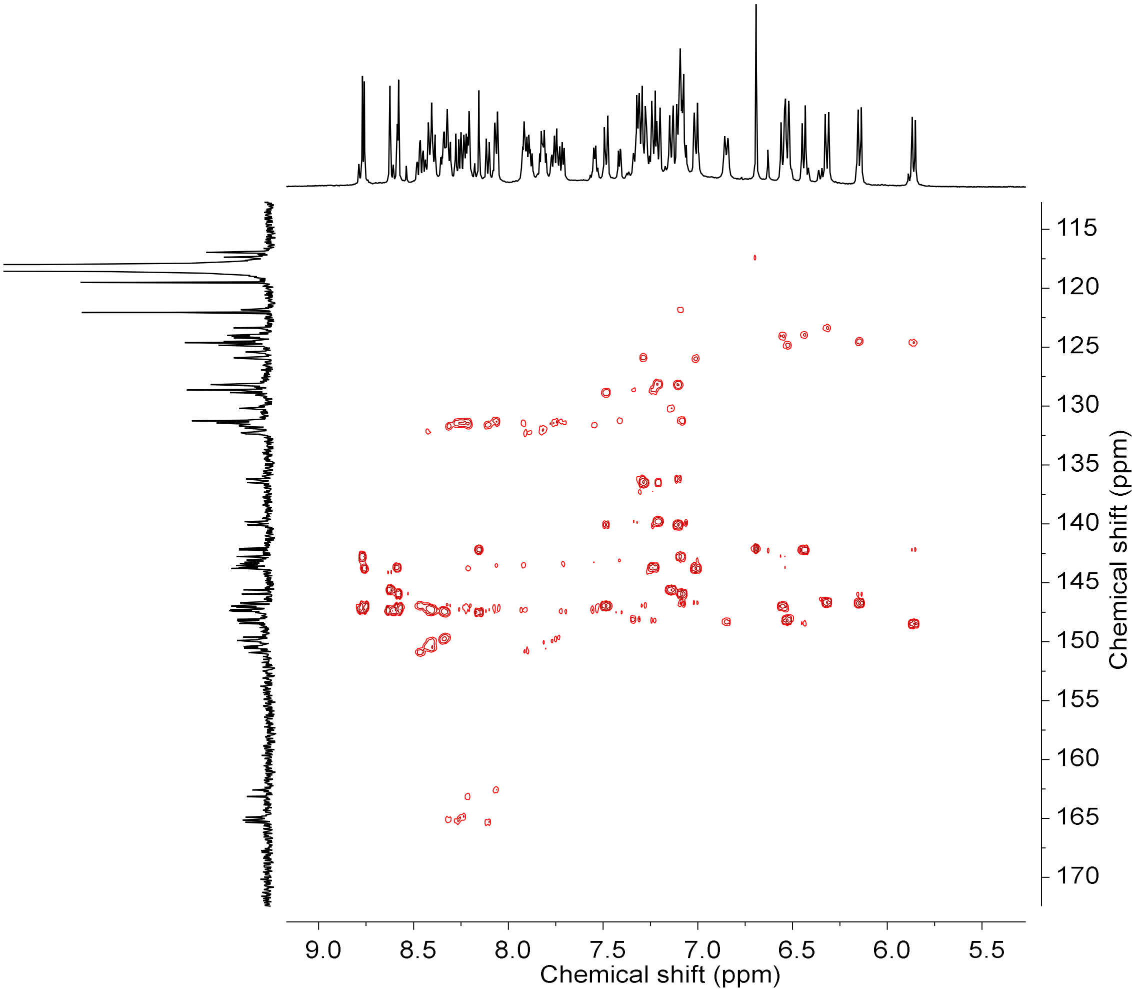

Figure S45. Aromatic region of the ^{1}H-^{13}C HMBC spectrum (500 MHz, CD_3CN, 298 K) of **4**·$(NTf_2)_{16}$.

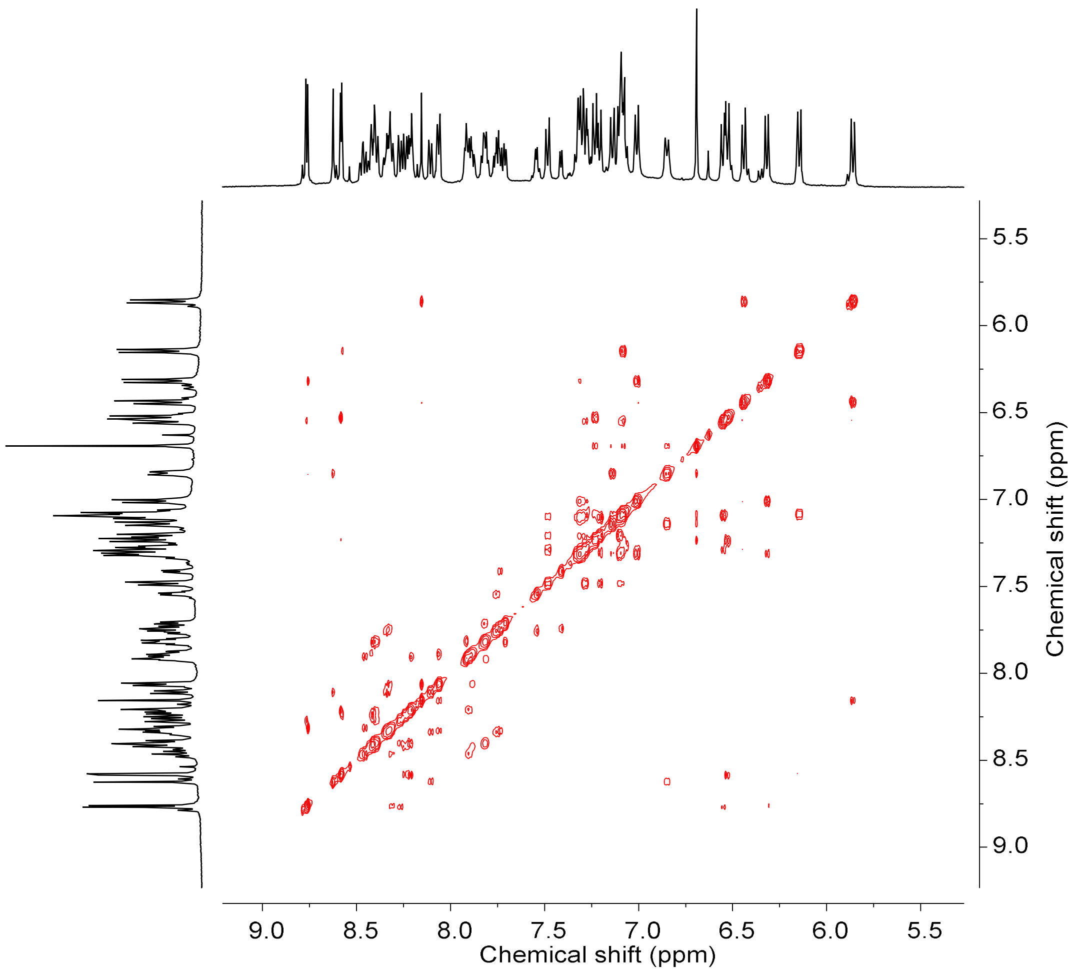

Figure S46. Aromatic region of the ^1H-^1H NOESY spectrum (500 MHz, CD_3CN, 298 K) of **4**·$(NTf_2)_{16}$.

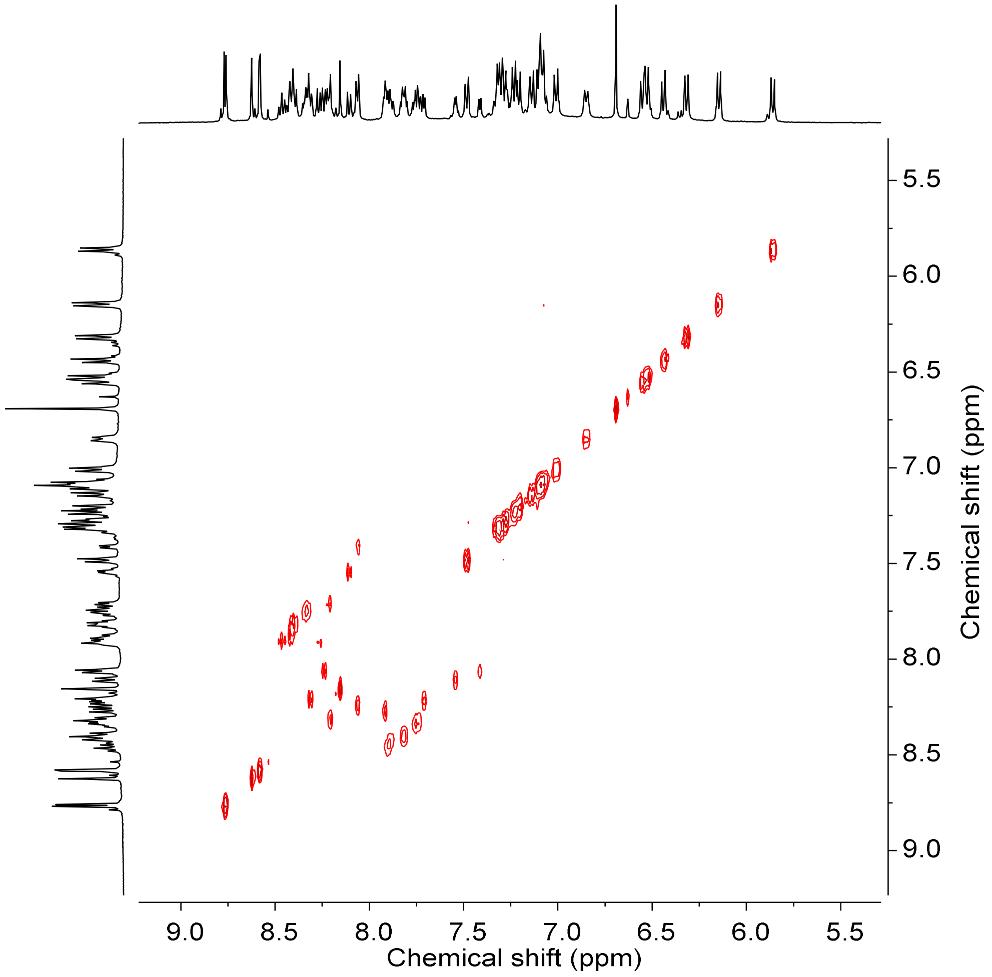

Figure S47. Aromatic region of the ^1H-^1H TOCSY spectrum (500 MHz, CD_3CN, 298 K) of **4**·$(NTf_2)_{16}$.

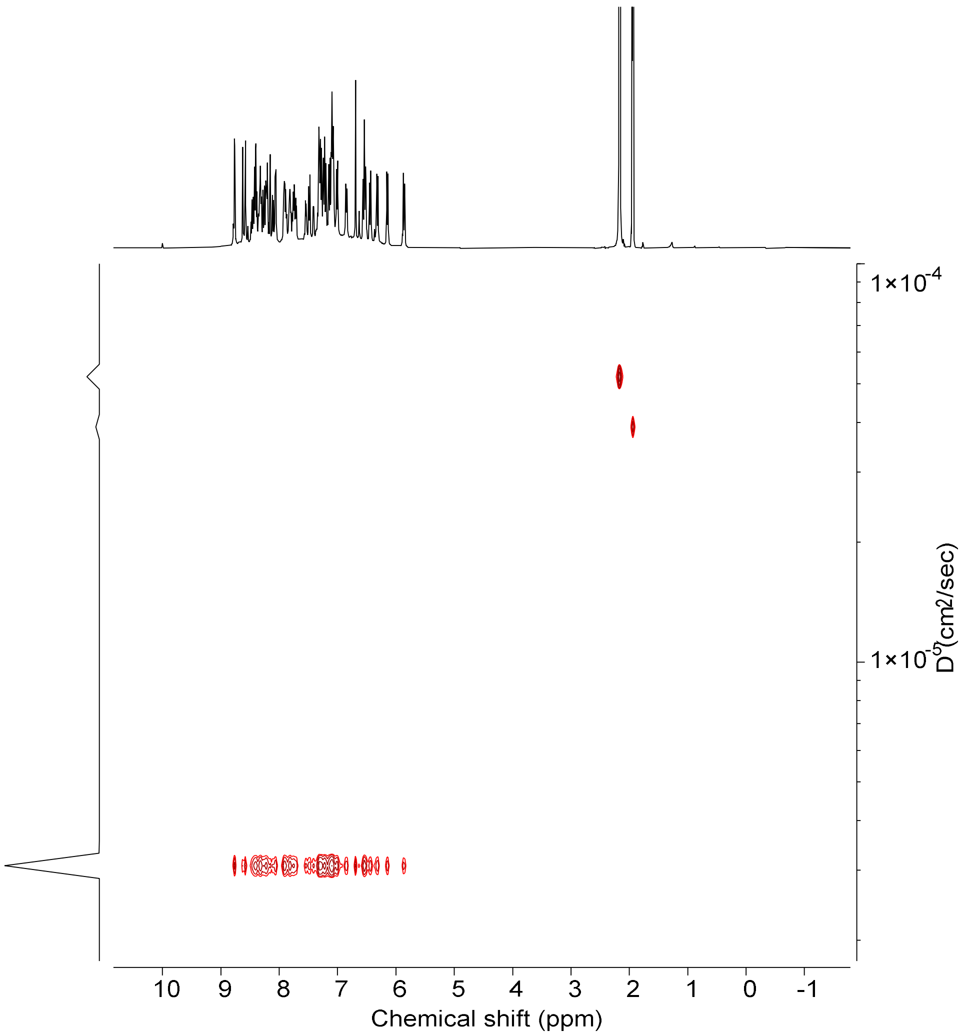

Figure S48. ^{1}H DOSY spectrum (400 MHz, CD_3CN, 298 K) of **4**·$(NTf_2)_{16}$.

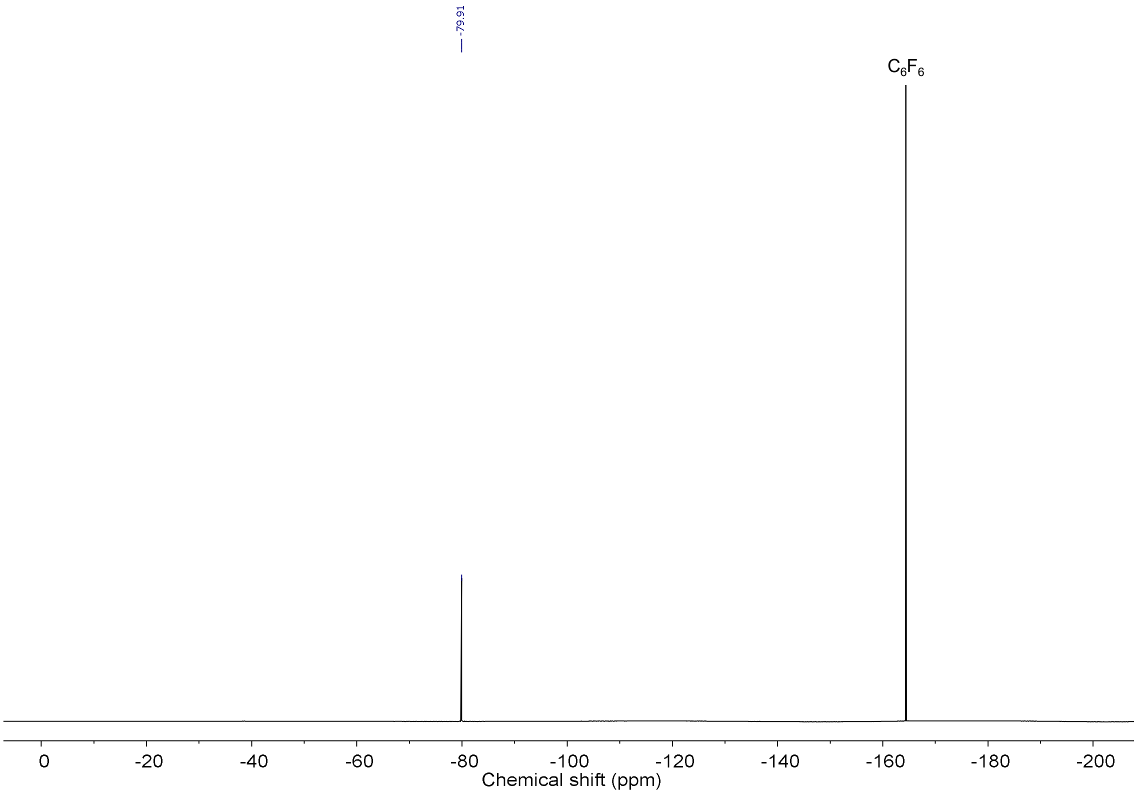

Figure S49. ^{19}F NMR spectrum (376 MHz, CD_3CN, 298 K) of **4**·$(NTf_2)_{16}$.

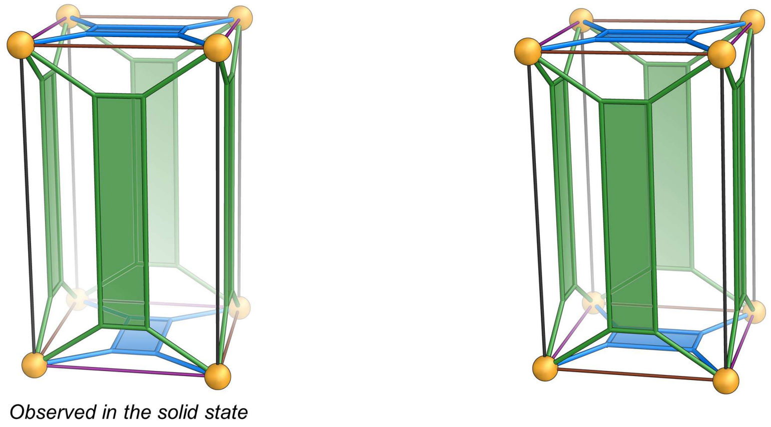

Figure S50. Schematic representation of two diastereomeric configurations of a tetragonal prism, differing based on the orientations of the low aspect ratio (blue) tetra-aniline residues with respect to each other. In the configuration on the left the long axes of the two low aspect ratio tetra-aniline residues are oriented perpendicular with respect to each other, while in the configuration on the right they are aligned parallel. Given that they contain the same edge types, with the same frequency, it was hypothesised that these configurations may have similar energies. In both configurations, all eight metal centres have the same handedness.

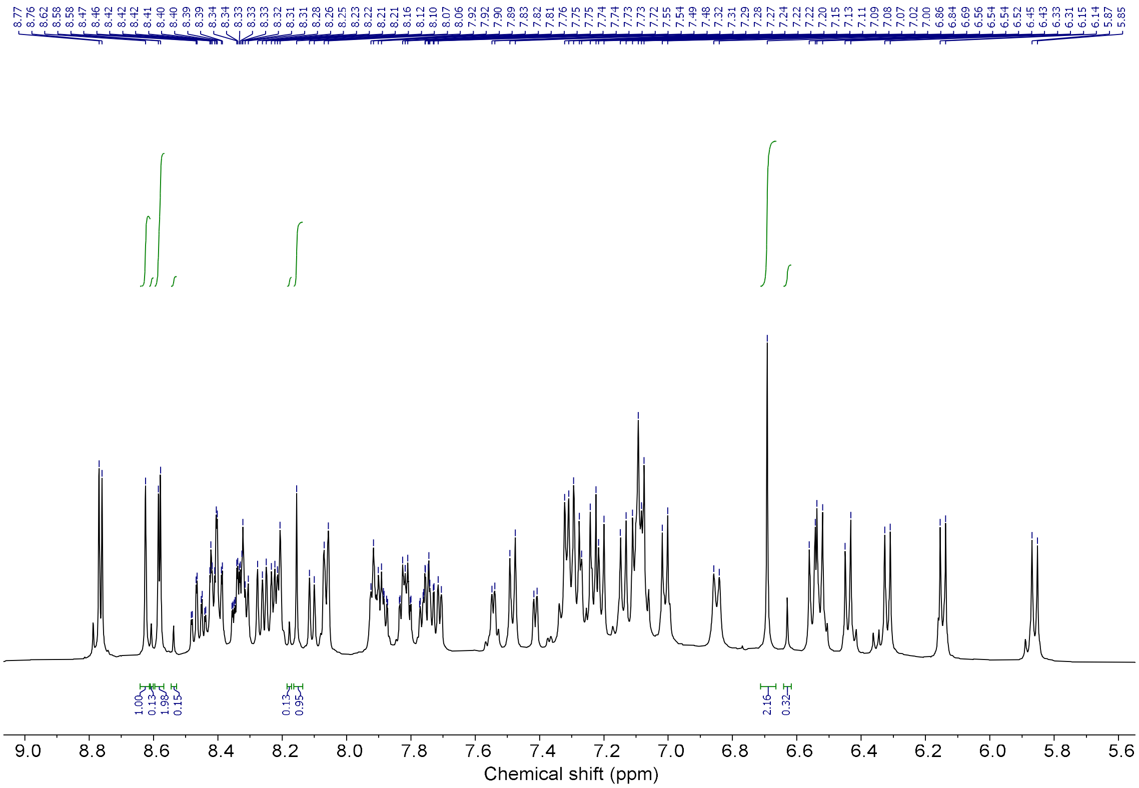

Figure S51. Aromatic region of the ^{1}H NMR spectrum (500 MHz, CD_3CN, 298 K) of **4**·$(NTf_2)_{16}$, with the relative integrated peak intensities used to estimate the diastereomeric ratio to be approximately 7:1 highlighted.

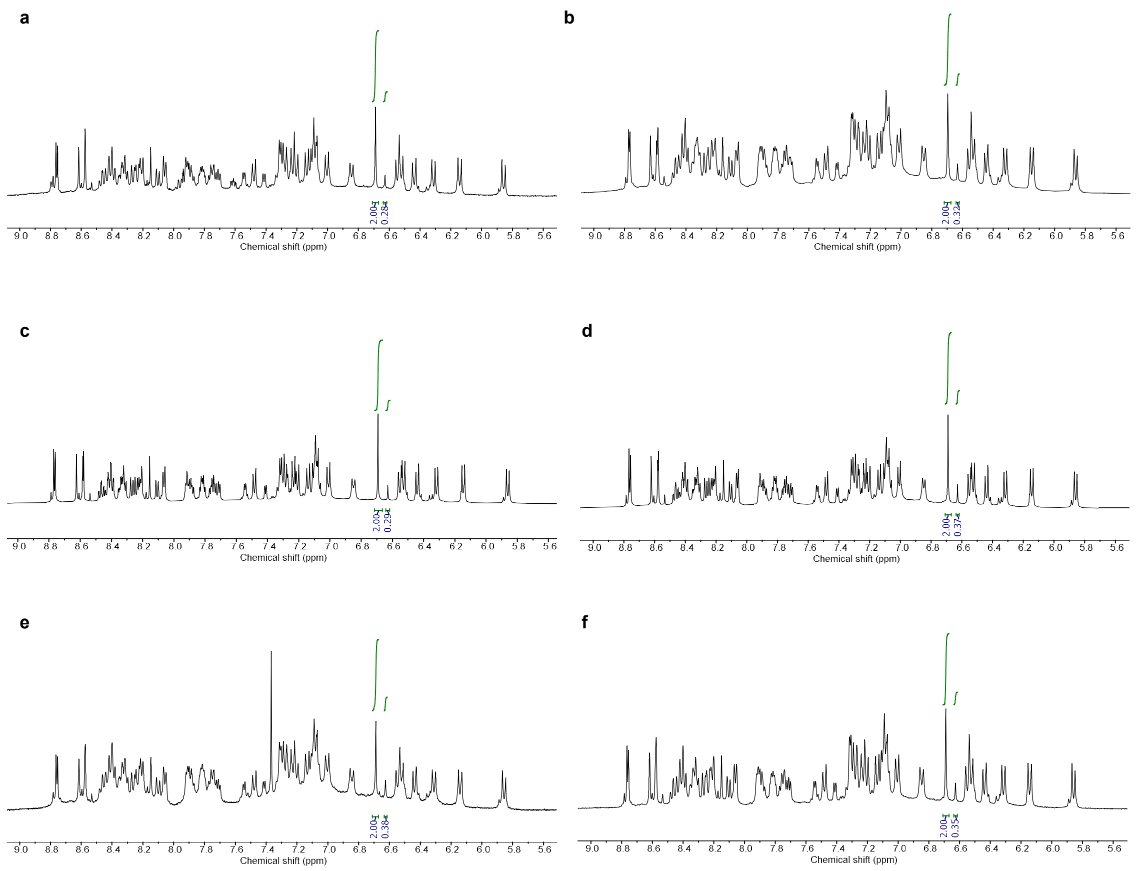

Figure S52. Aromatic region of the ^{1}H NMR spectra (400 MHz (a, b, e, f) or 500 MHz (c, d), CD_3CN, 298 K) of **4**·$(NTf_2)_{16}$ prepared under a range of different experimental conditions. The relative integrated peak intensities used to estimate the diastereomeric ratio are highlighted. a) [**A**] = 2.2 mM, heated at 343 K for 16 hours. b) [**A**] = 0.3 mM, heated at 393 K in a microwave reactor for 2 hours. c) [**A**] = 0.6 mM, heated at 393 K in a microwave reactor for 10 hours. d) [**A**] = 0.9 mM, heated at 393 K in a microwave reactor for 6 hours. e) [**A**] = 1.0 mM, heated at 393 K in a microwave reactor for 2 hours. f) [**A**] = 2.0 mM, heated at 393 K in a microwave reactor for 4 hours.

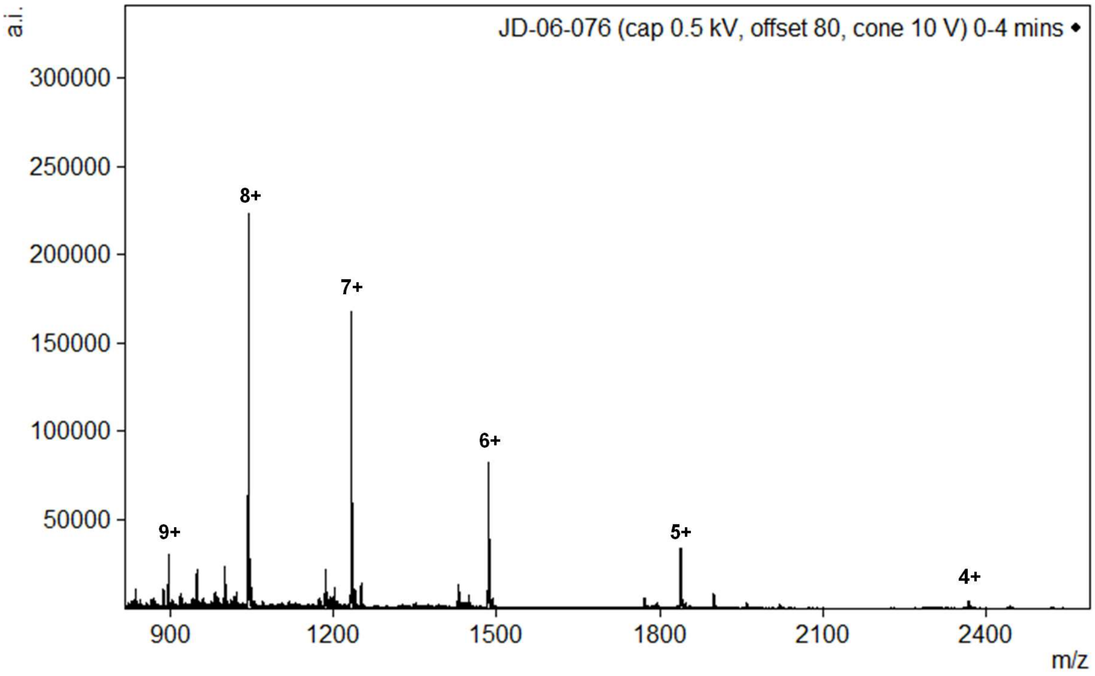

Figure S53. High resolution ESI-mass spectrum for **4**·$(NTf_2)_{16}$.

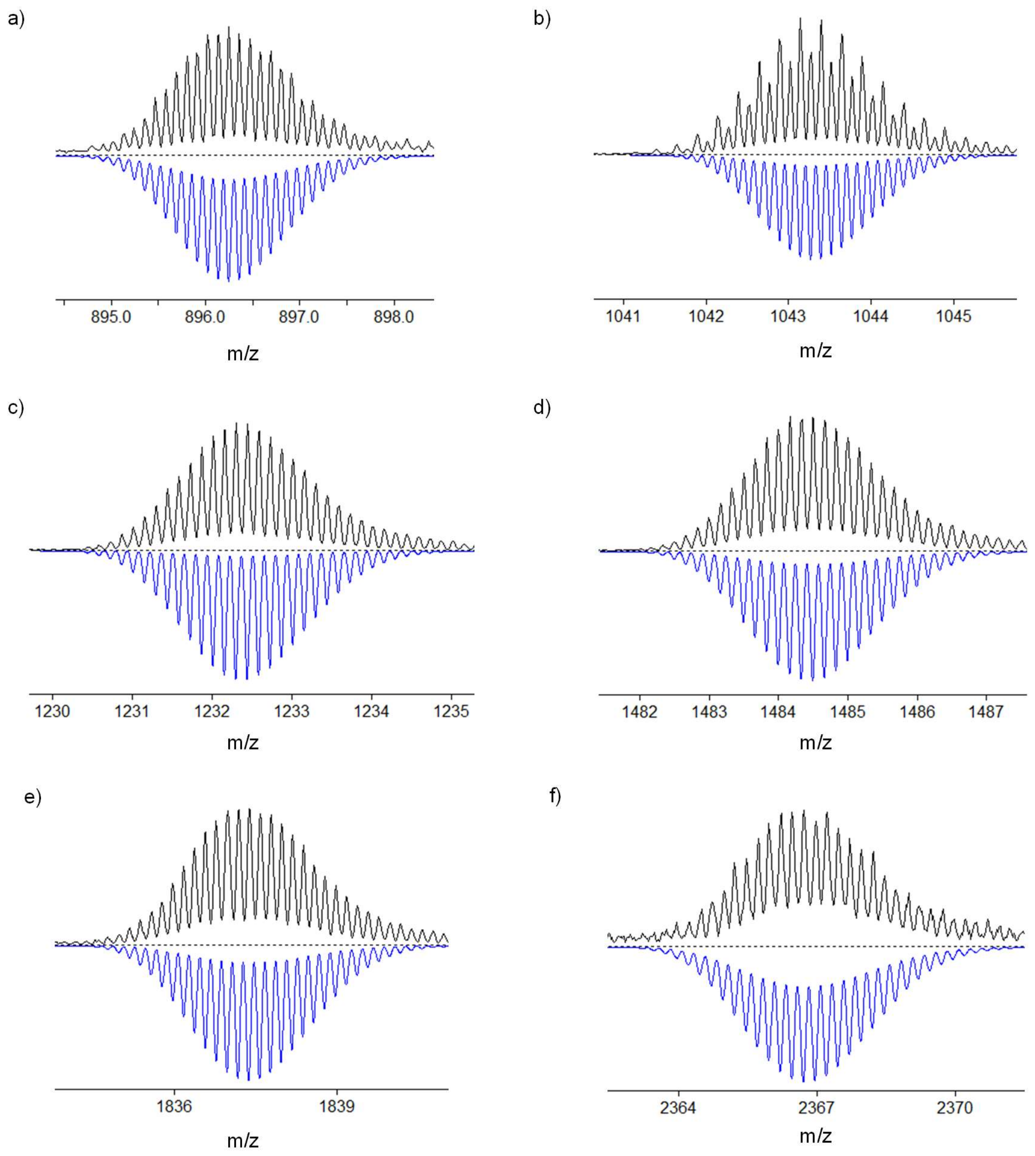

Figure S54. Signals from the high resolution ESI-mass spectrum for **4**·$(NTf_2)_{16}$. Experimental (black) and calculated (blue) signals for a) [**4**$(NTf_2)_7]^{9+}$ b) [**4**$(NTf_2)_8]^{8+}$ c) [**4**$(NTf_2)_9]^{7+}$ d) [**4**$(NTf_2)_{10}]^{6+}$ e) [**4**$(NTf_2)_{11}]^{5+}$ f) [**4**$(NTf_2)_{12}]^{4+}$.

3.5 Synthesis and characterization of 5

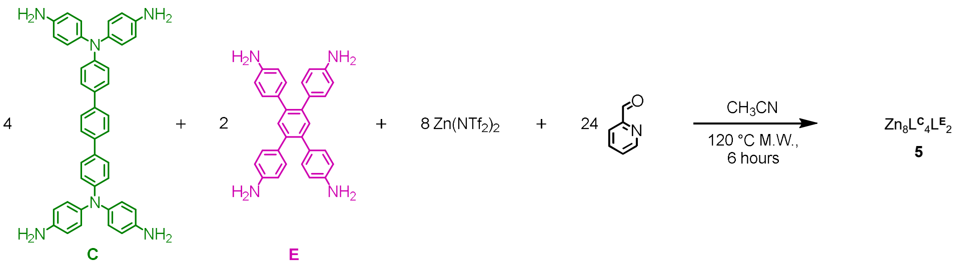

Scheme S6. Subcomponent self-assembly of **5**·$(NTf_2)_{16}$.

To a mixture of tetra-aniline **E** (1.2 mg, 2.7 μmol, 1.0 equiv), tetra-aniline **C** (3.6 mg, 5.8 μmol, 2.1 equiv) and $Zn(NTf_2)_2$ (7.7 mg, 12.3 μmol, 4.6 equiv) in acetonitrile (3.0 mL), 2-formylpyridine (3.4 μL, 35.7 μmol, 13.2 equiv) was added. The reaction mixture was heated at 120 °C for 6 hours in a microwave reactor. The reaction mixture was allowed to cool to RT, filtered through a glass fibre plug, concentrated to a small volume and diethyl ether (8 mL) was added. The solid was washed with diethyl ether (3 × 8 mL) and the product **5**·$(NTf_2)_{16}$ was obtained as a very dark red/purple solid (15.1 mg, quant.).

^{1}H NMR (700 MHz, CD_3CN, 298 K) δ 8.84 (app. s, 8H), 8.52 (s, 4H), 8.49 (s, 4H), 8.47–8.41 (m, 16H), 8.38 (s, 4H), 8.37–8.28 (m, 16H), 8.26 (s, 4H), 8.22 (d, *J* = 7.9 Hz, 4H), 8.17 (d, *J* = 7.8 Hz, 4H), 8.10 (d, *J* = 4.8 Hz, 4H), 8.09–8.06 (m, 8H), 8.04 (d, *J* = 4.9 Hz, 4H), 7.99–7.96 (m, 8H), 7.91 (dd, *J* = 7.6, 5.1 Hz, 4H), 7.89–7.82 (m, 12H), 7.77–7.73 (m, 8H), 7.48–7.42 (m, 8H), 7.39 (d, *J* = 7.5 Hz, 4H), 7.34 (app. d, *J* = 4.6 Hz, 8H), 7.27–7.22 (m, 16H), 7.17 (d, *J* = 8.4 Hz, 8H), 7.11–7.07 (m, 8H), 7.06–6.95 (m, 8H), 6.60–6.43 (m, 20H), 6.29 (d, *J* = 8.0 Hz, 8H), 6.15 (d, *J* = 8.2 Hz, 8H), 6.03–5.91 (m, 8H). Some expected peaks could not be identified due to the broadening of signals in the region δ = 7.50–5.90 ppm. Due to the broadening of signals and signal overlap, the relative integrations of some signals in the region δ = 7.50–5.90 ppm are thus likely to be different than expected. The broadening of the signals is caused by dynamic processes occurring at an intermediate rate with respect to the NMR timescale. A variable temperature ^{1}H NMR spectroscopy study allowed the unidentified signals to be located (Figure S64).

^{13}C NMR (176 MHz, CD_3CN, 298 K) δ 165.6, 165.1, 165.0, 164.4, 163.8, 163.4, 150.9, 150.8, 150.5, 150.2, 149.8, 149.7, 149.1, 148.2, 147.7, 147.4, 147.4, 147.3, 147.2, 147.1, 146.9, 146.9, 146.5, 146.1, 144.8, 143.7, 143.6, 143.3, 143.3, 143.0, 142.7, 142.5, 141.7, 140.3, 140.1, 139.7, 139.1, 137.0, 136.8, 135.4, 132.4, 132.1, 131.9, 131.9, 131.7, 131.6, 131.5, 131.4, 131.4, 131.2, 131.1, 128.8, 128.4, 128.2, 127.5, 125.4, 124.8, 124.1, 123.0, 122.9, 121. 5, 120.8 (q, *J* = 321 Hz). Some of the expected peaks were not identified, likely due to signal overlap and dynamic processes occurring at room temperature.

ESI-HRMS ([**5**$(NTf_2)_{16}$] = $C_{372}H_{268}N_{56}Zn_8(C_2F_6NO_4S_2)_{16}$) *m/z* = 889.5646 [**5**$(NTf_2)_7$]$^{9+}$ (calc. 889.5685), 1035.8751 [**5**$(NTf_2)_8$]$^{8+}$ (calc. 1035.8790), 1223.8467 [**5**$(NTf_2)_9$]$^{7+}$ (calc. 1223.8503), 1474.4749 [**5**$(NTf_2)_{10}$]$^{6+}$ (calc. 1474.4779), 1825.3554 [**5**$(NTf_2)_{11}$]$^{5+}$ (calc. 1825.3574).

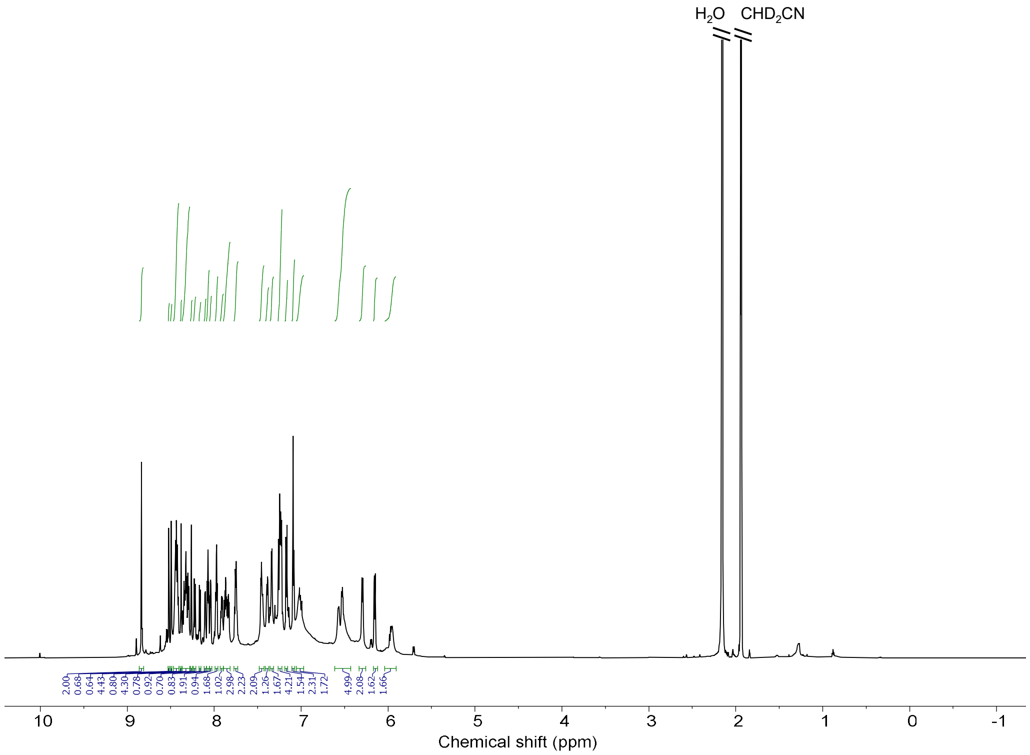

Figure S55. ^{1}H NMR spectrum (700 MHz, CD_3CN, 298 K) of **5**·$(NTf_2)_{16}$.

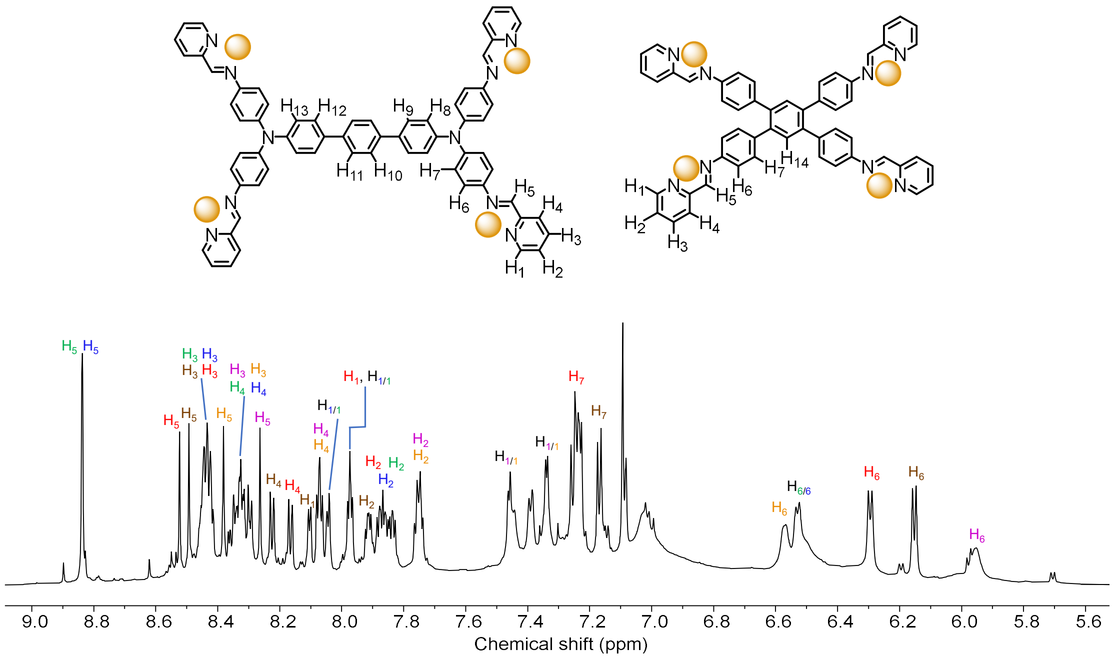

Figure S56. Aromatic region of the ^{1}H NMR spectrum (700 MHz, CD_3CN, 298 K) of **5**·$(NTf_2)_{16}$, with assignment of signals for the major species. The ^{1}H NMR signals for the proton environments $H_{6/6}$, H_7, H_7, H_7, H_7, H_{8-14} could not be assigned. Assignment of the signals for these proton environments was carried out following a variable temperature ^{1}H NMR spectroscopy study (Figure S64).

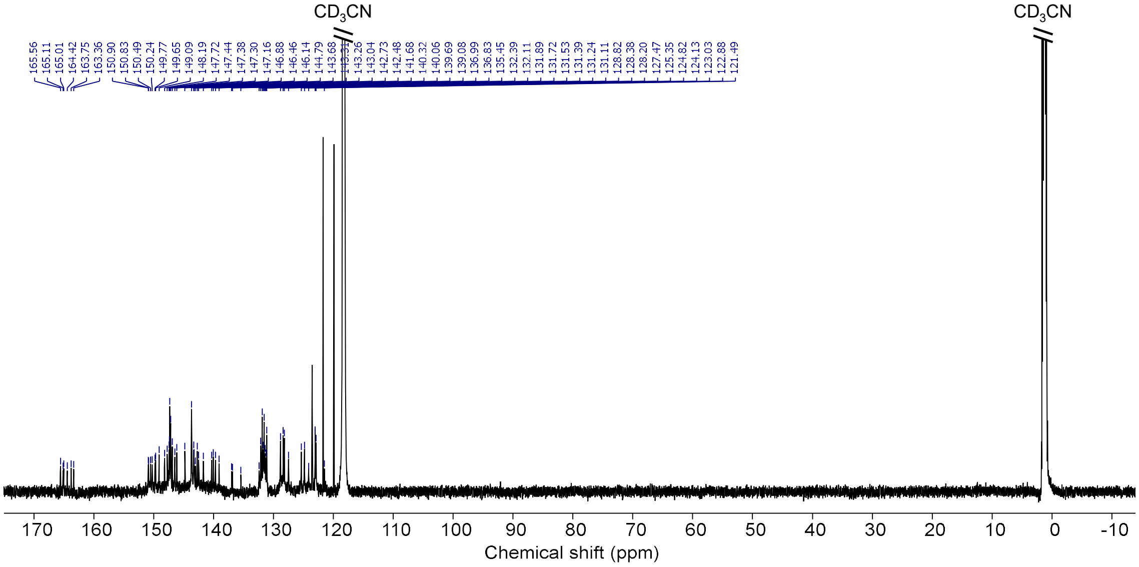

Figure S57. ^{13}C NMR spectrum (176 MHz, CD_3CN, 298 K) of **5**·$(NTf_2)_{16}$.

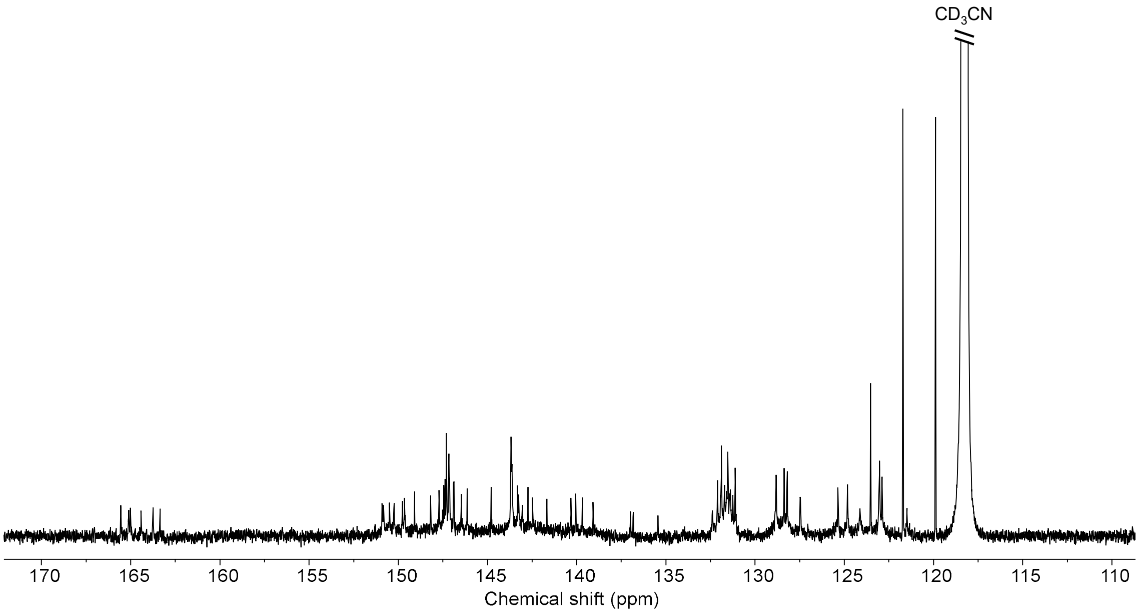

Figure S58. Aromatic region of the ^{13}C NMR spectrum (176 MHz, CD_3CN, 298 K) of **5**·$(NTf_2)_{16}$.

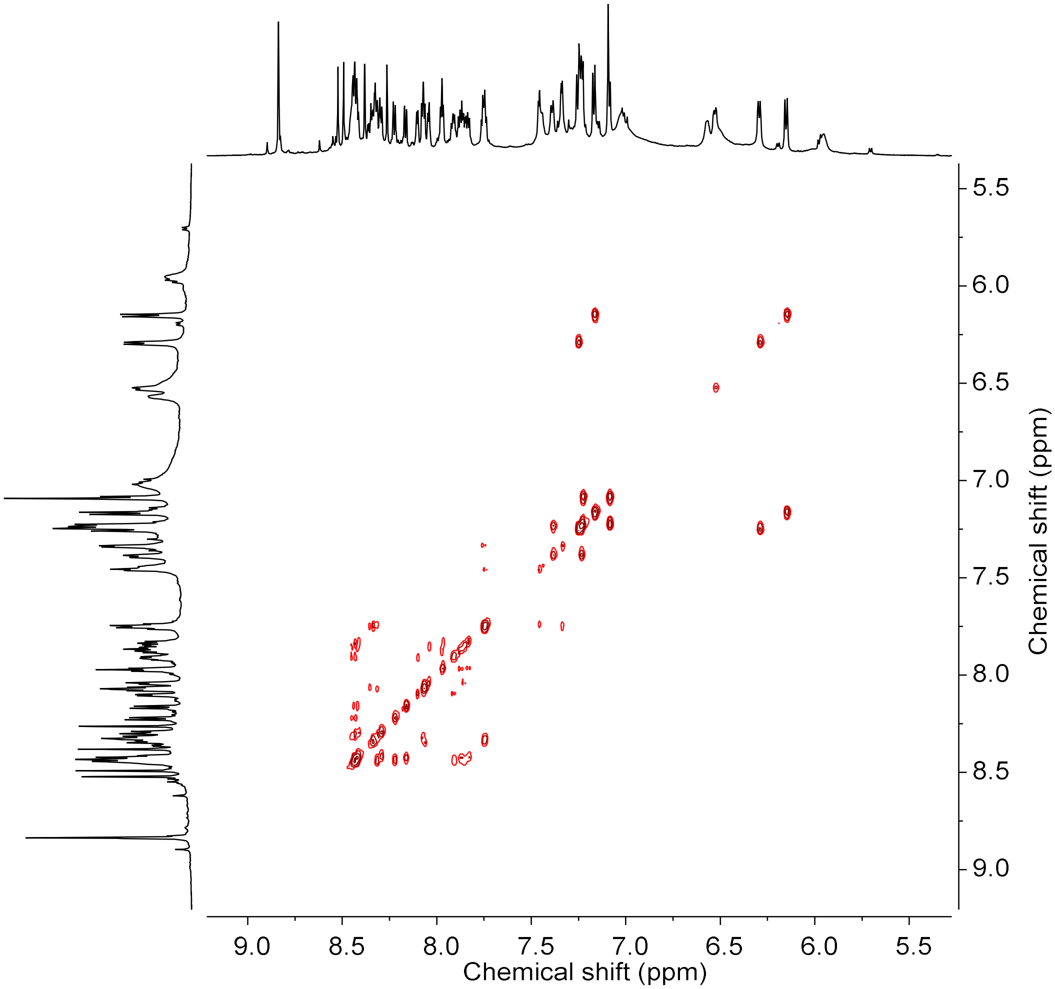

Figure S59. Aromatic region of the ^1H-^1H DQF-COSY spectrum (700 MHz, CD_3CN, 298 K) of **5**·$(NTf_2)_{16}$.

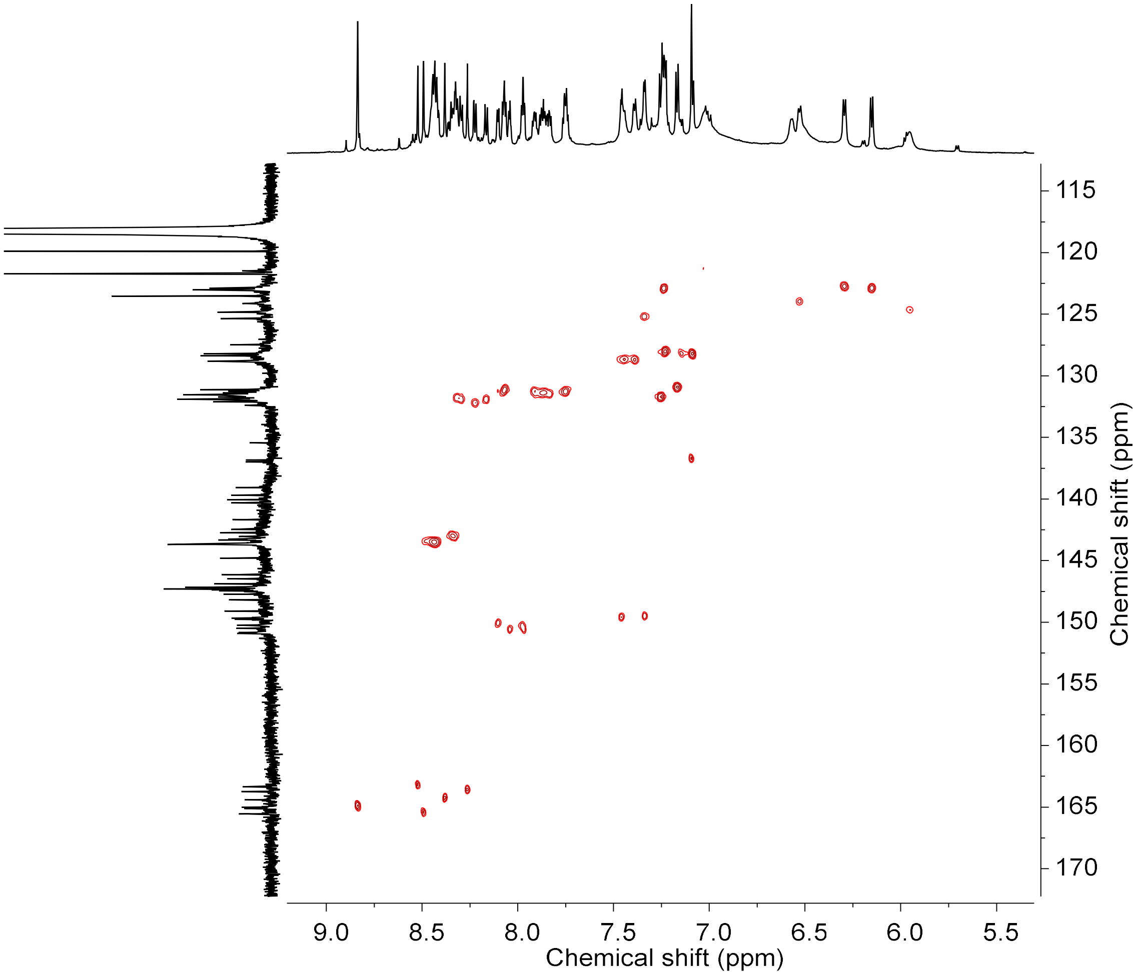

Figure S60. Aromatic region of the ^{1}H-^{13}C edited HSQC spectrum (700 MHz, CD_3CN, 298 K) of **5**·$(NTf_2)_{16}$.

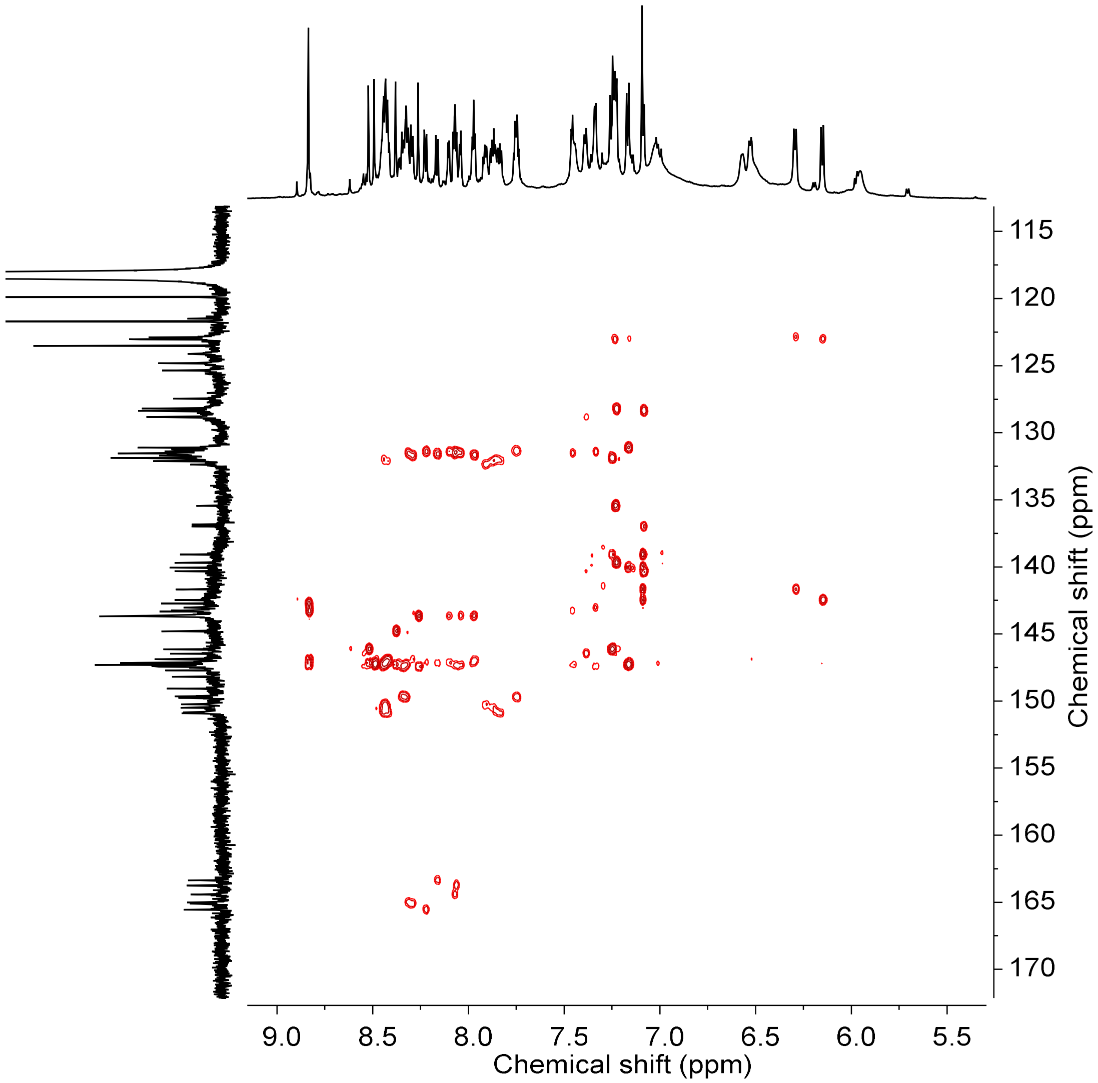

Figure S61. Aromatic region of the ^{1}H-^{13}C HMBC spectrum (700 MHz, CD_3CN, 298 K) of **5**·$(NTf_2)_{16}$.

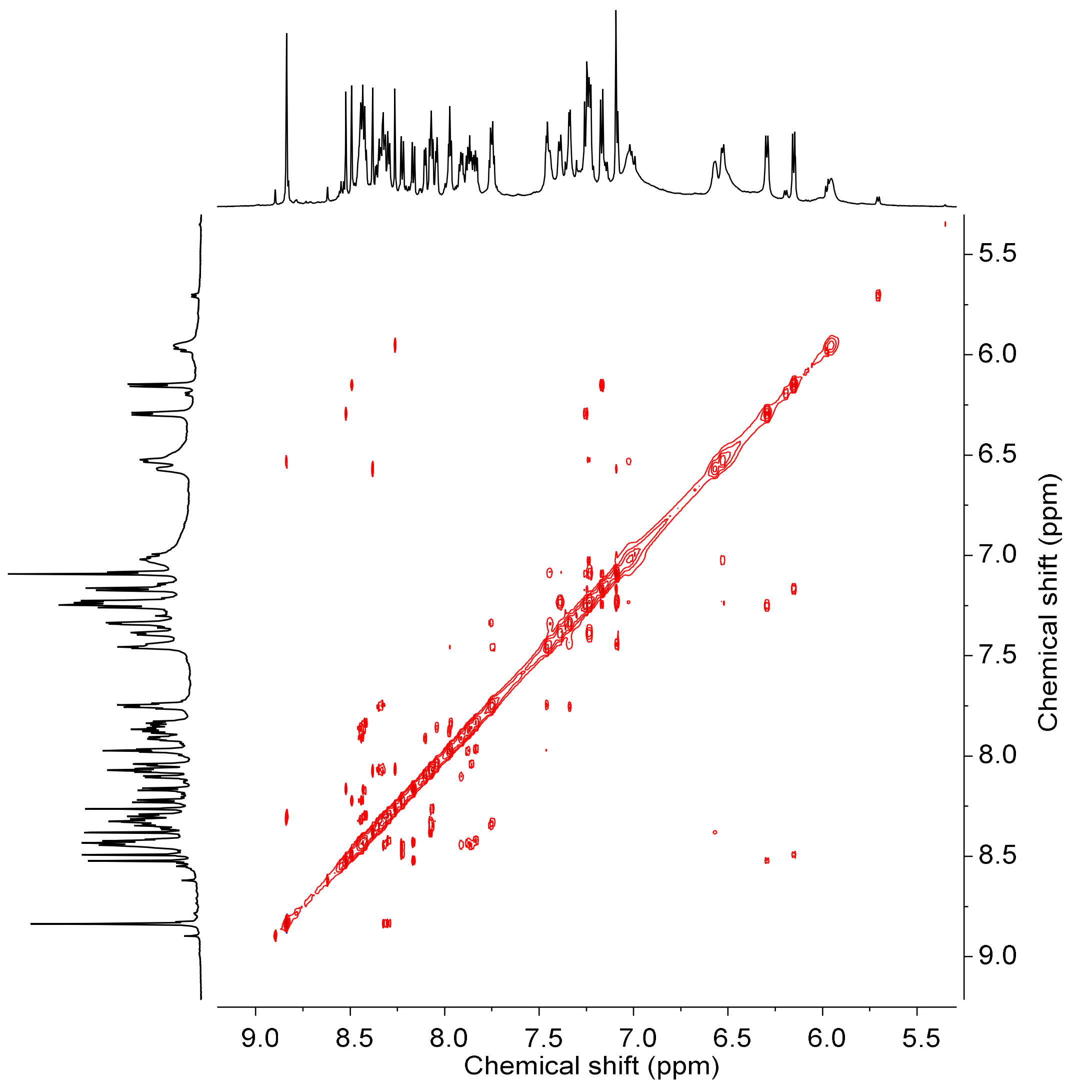

Figure S62. Aromatic region of the ^1H-^1H NOESY spectrum (700 MHz, CD_3CN, 298 K) of **5**·$(NTf_2)_{16}$.

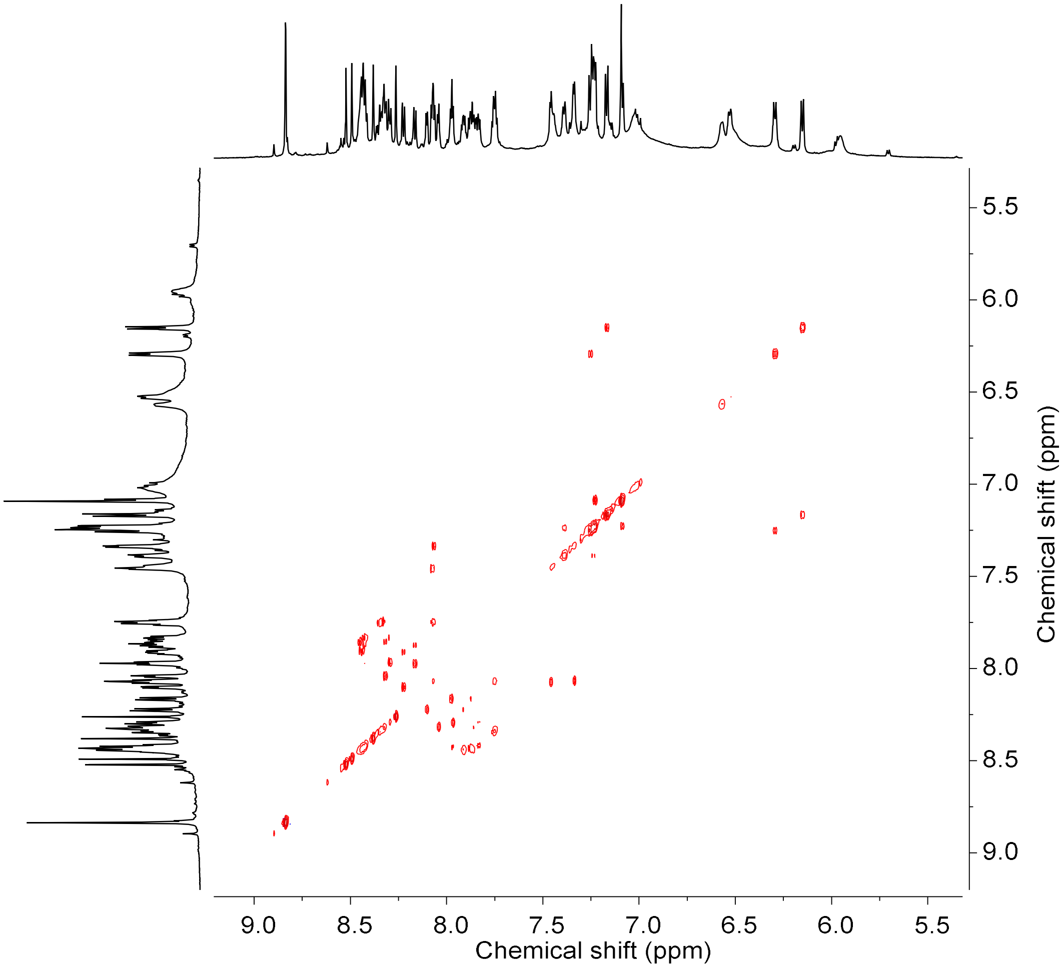

Figure S63. Aromatic region of the ^1H-^1H TOCSY spectrum (700 MHz, CD_3CN, 298 K) of **5**·$(NTf_2)_{16}$.

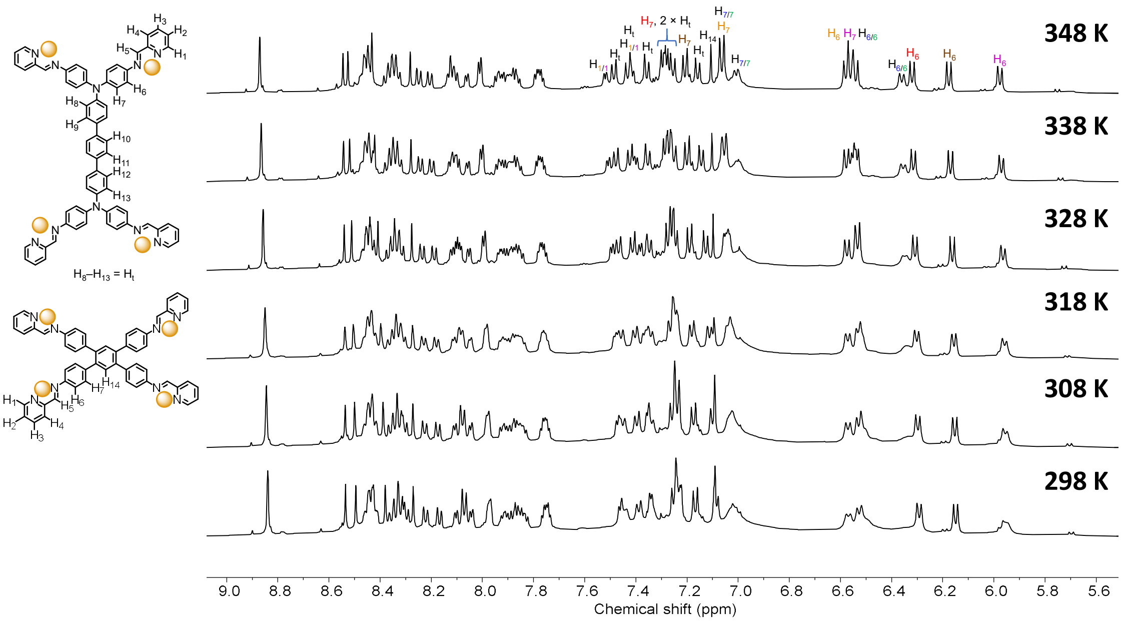

Figure S64. Aromatic region of ^{1}H NMR spectra (500 MHz, CD_3CN) of **5**·$(NTf_2)_{16}$ at 298 K, 308 K, 318 K, 328 K, 338 K, 348 K. The signals at δ = 7.50–5.90 ppm became sharper for the spectrum recorded at 348 K (compared to 298 K), allowing the assignment of signals to proton environments on the ligands to be completed.

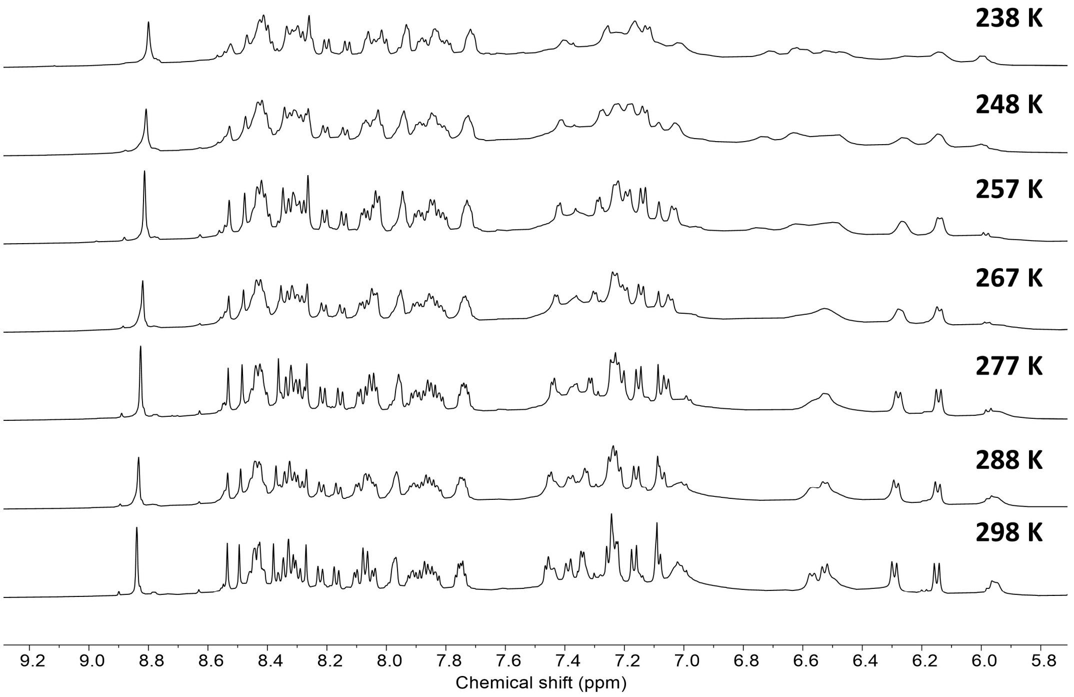

Figure S65. Aromatic region of ^{1}H NMR spectra (500 MHz, CD_3CN) of **5**·$(NTf_2)_{16}$ at 298 K, 288 K, 277 K, 267 K, 257 K, 248 K, 238 K. The signals became broader with decreasing temperature.

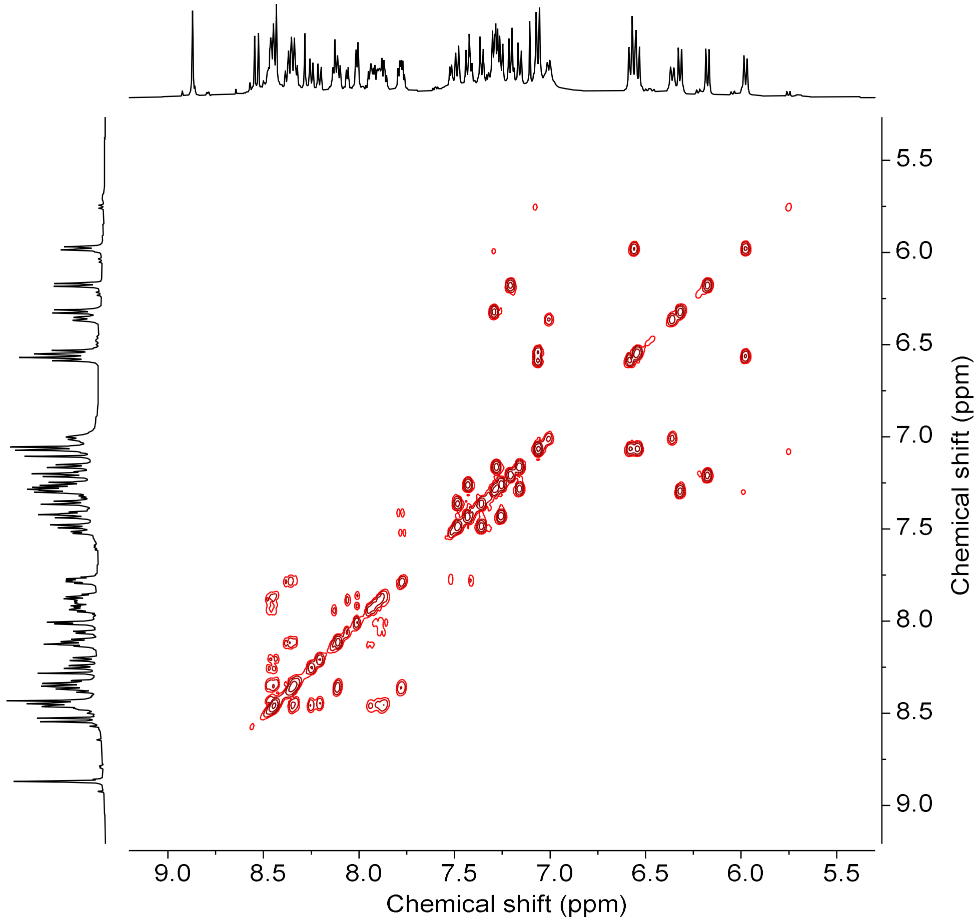

Figure S66. Aromatic region of the ^1H-^1H COSY spectrum (500 MHz, CD_3CN, 348K) of **5**·$(NTf_2)_{16}$.

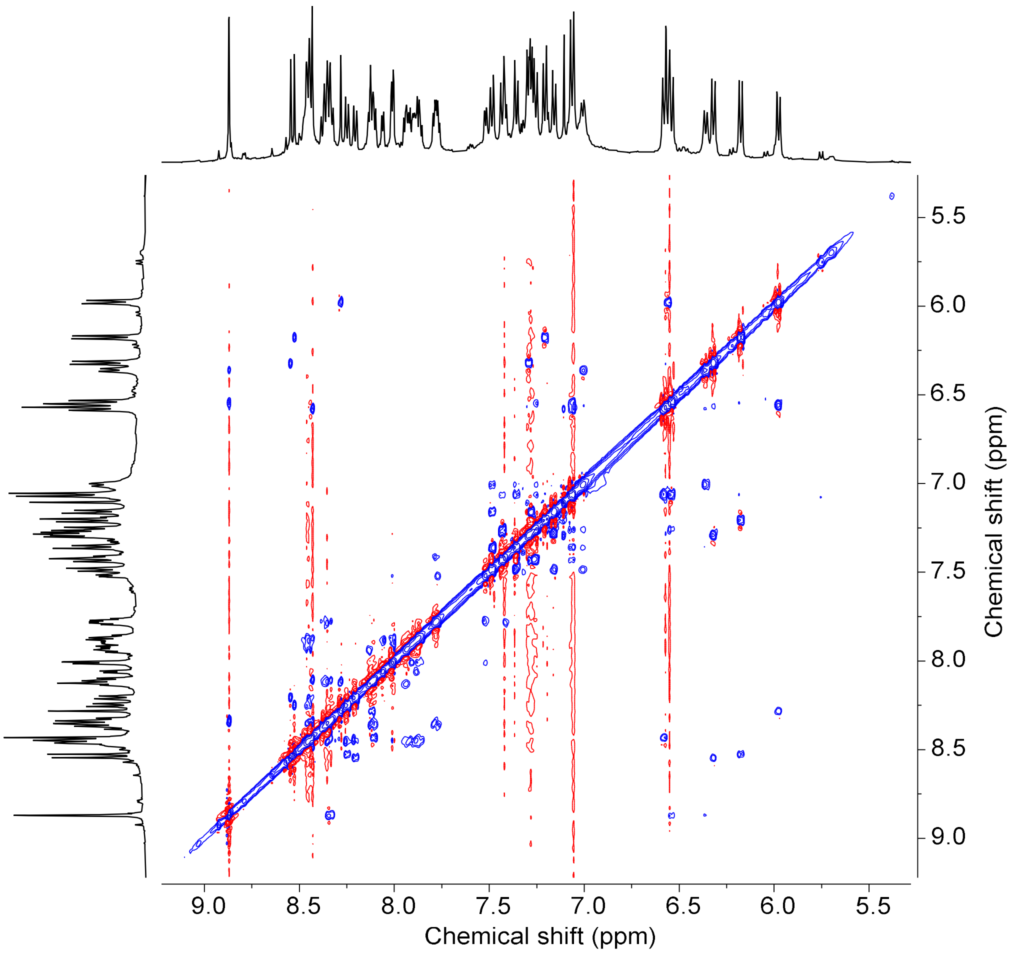

Figure S67. Aromatic region of the ^1H-^1H NOESY spectrum (500 MHz, CD_3CN, 348 K) of **5**·$(NTf_2)_{16}$.

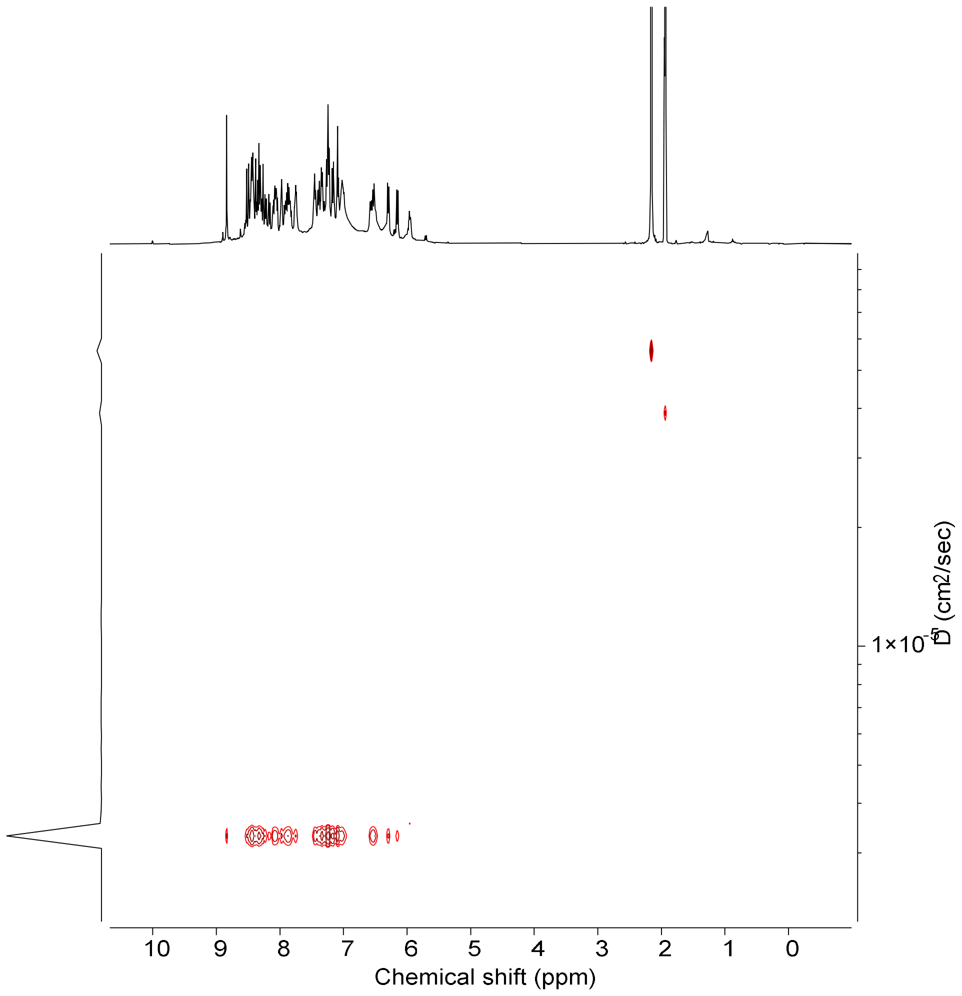

Figure S68. ^{1}H DOSY spectrum (400 MHz, CD_3CN, 298 K) of **5**·$(NTf_2)_{16}$.

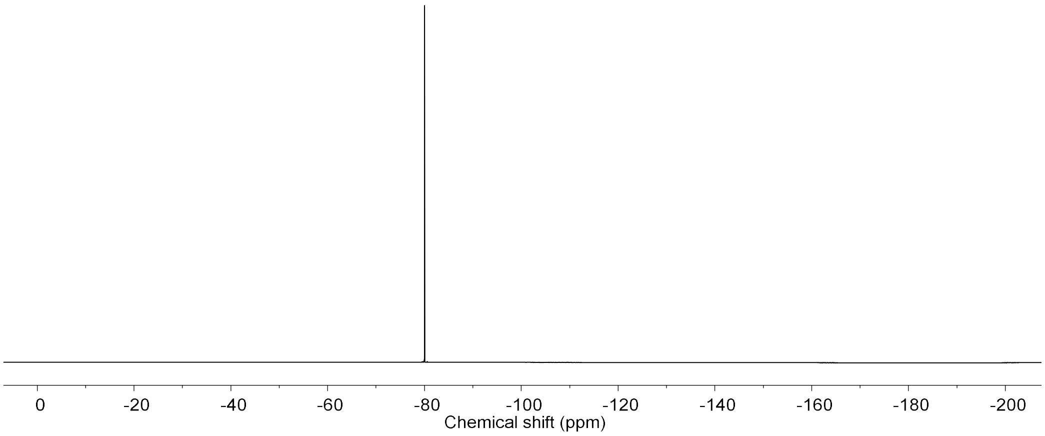

Figure S69. ^{19}F NMR spectrum (376 MHz, CD_3CN, 298 K) of **5**·$(NTf_2)_{16}$.

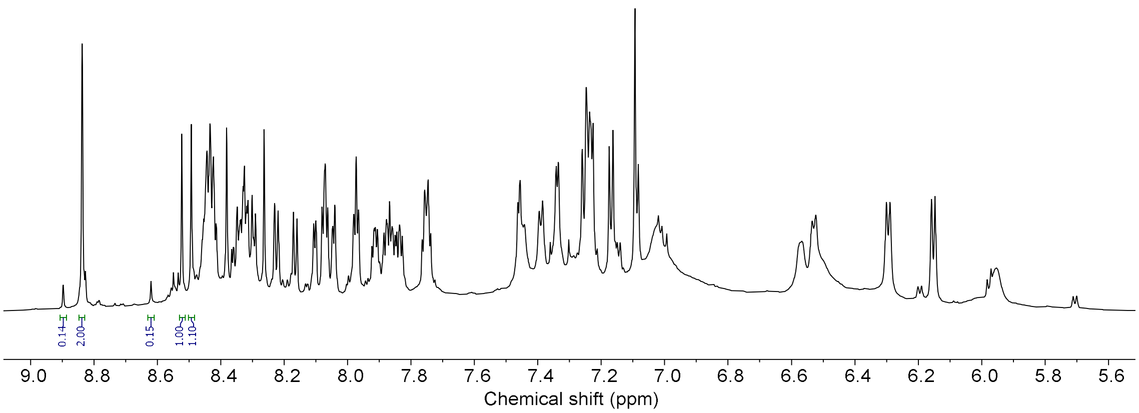

Figure S70. Aromatic region of the ^{1}H NMR spectrum (700 MHz, CD_3CN, 298 K) of **5**·$(NTf_2)_{16}$. The relative integrated peak intensities used to estimate the diastereomeric ratio to be approximately 7:1 are highlighted.

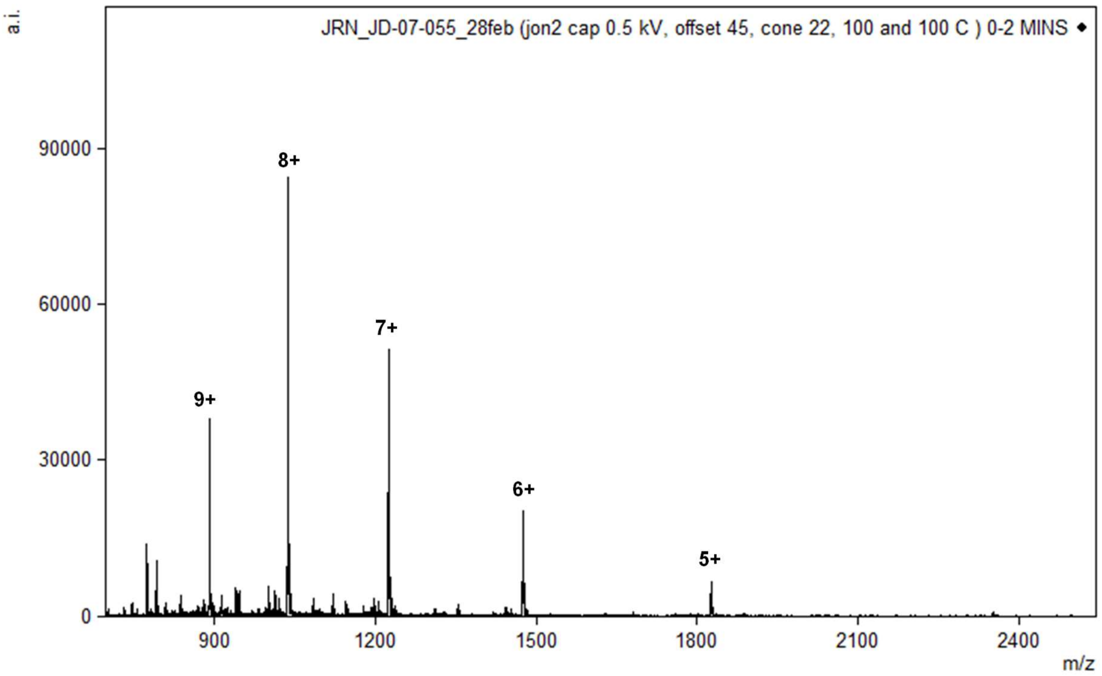

Figure S71. High resolution ESI-mass spectrum for **5**·$(NTf_2)_{16}$.

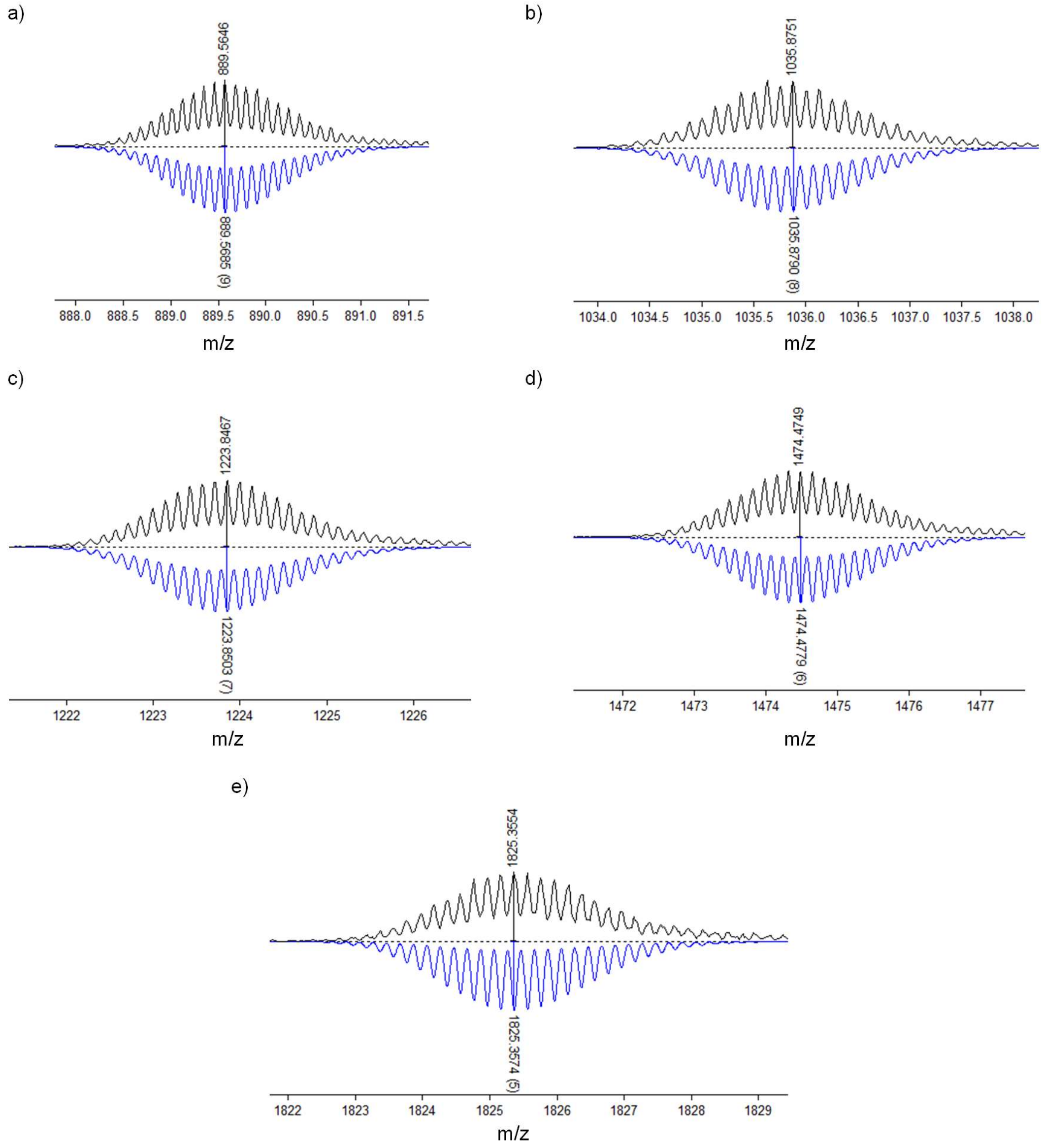

Figure S72. Signals from the high resolution ESI-mass spectrum for **5**·$(NTf_2)_{16}$. Experimental (black) and calculated (blue) signals for a) [**5**$(NTf_2)_7]^{9+}$ b) [**5**$(NTf_2)_8]^{8+}$ c) [**5**$(NTf_2)_9]^{7+}$ d) [**5**$(NTf_2)_{10}]^{6+}$ e) [**5**$(NTf_2)_{11}]^{5+}$.

4 X-ray crystallography

Data were collected at Beamline I19 of Diamond Light Source employing silicon double crystal monochromated synchrotron radiation (0.6889 Å) with ω and ψ scans at 100(2) K.[4] Data integration and reduction were undertaken with Xia2.[5] Subsequent computations were carried out using the WinGX-32 graphical user interface.[6] Multi-scan empirical absorption corrections were applied to the data using the AIMLESS[7] tool in the CCP4 suite.[8] The structures were solved by direct methods using SHELXT[9] then refined and extended with SHELXL.[10] In general, non-hydrogen atoms with occupancies

greater than 0.5 were refined anisotropically. Carbon-bound hydrogen atoms were included in idealized positions and refined using a riding model. Disorder was modelled using standard crystallographic methods including constraints, restraints and rigid bodies where necessary. Crystallographic data along with specific details pertaining to the refinement follow. Crystallographic data have been deposited with the CCDC (2179386 and 2179388–2179390).

1·12ReO$_4$·4.75CH$_3$CN·1.5H$_2$O [+ solvent]

Formula $C_{243.50}H_{191.25}N_{48.75}O_{49.50}Re_{12}Zn_6$, *M* 7218.81, Monoclinic, space group P 21/c (#14), *a* 21.5610(2), *b* 38.2329(4), *c* 34.7766(3) Å, β 99.0420(10), *V* 28311.5(5) Å^3, D_c 1.694 g cm^{-3}, *Z* 4, crystal size 0.050 by 0.040 by 0.030 mm, colour red brown, habit block, temperature 100(2) Kelvin, λ(synchrotron) 0.6889 Å, μ(synchrotron) 5.201 mm^{-1}, *T*(Analytical)$_{min,max}$ 0.9780239538362973, 1.0, 2θ_{max} 45.00, *hkl* range -23 23, -42 42, -38 34, *N* 171430, N_{ind} 39995(R_{merge} 0.0776), N_{obs} 19461(I > 2σ(I)), N_{var} 3450, residuals* *R*1(*F*) 0.0684, *wR*2(F^2) 0.1853, GoF(all) 1.002, $\Delta\rho_{min,max}$ -1.266, 1.447 e$^-$ Å^{-3}.

*$R1 = \Sigma||F_o| - |F_c||/\Sigma|F_o|$ for $F_o > 2\sigma(F_o)$; $wR2 = (\Sigma w(F_o^2 - F_c^2)^2/\Sigma(wF_c^2)^2)^{1/2}$ all reflections

$w=1/[\sigma^2(F_o^2)+(0.0900P)^2]$ where $P=(F_o^2+2F_c^2)/3$

Specific refinement details:

The crystals of **1**·12ReO$_4$·4.75CH$_3$CN·1.5H$_2$O [+ solvent] were grown by diffusion of diisopropyl ether into an acetonitrile solution of **1**·12NTf$_2$ containing excess ^{n}Bu$_4$N·ReO$_4$ (^{n}Bu$_4$N = tetrabutylammonium). The crystals employed immediately lost solvent after removal from the mother liquor and rapid handling prior to flash cooling in liquid nitrogen was required to collect data. Despite these measures and the use of synchrotron radiation few reflections at greater than 0.9 Å resolution were observed and the data were trimmed accordingly. Nevertheless, the quality of the data is far more than sufficient to establish the connectivity of the structure. The asymmetric unit was found to contain one complete $Zn_6L_3L'_2$ assembly and associated counterions and solvent molecules. Due to the limited resolution bond lengths and angles within pairs of chemically identical organic ligands were restrained to be similar to each other and thermal parameter restraints (SIMU, RIGU) were applied to all atoms except for zinc and rhenium.

The anions within the structure also show evidence of substantial disorder. All located perrhenate anions were modelled as disordered over two or three locations and the occupancies of all located anions were freely refined. The oxygen atoms of some very low occupancy perrhenate anions located close to higher occupancy anions were not modelled. The overall refined occupancy of Re results in a discrepancy of ca. 1.6 anions per $Zn_6L_3L'_2$ assembly which could not be resolved, presumably due to them being highly disordered. The perrhenate anions were restrained to be approximately tetrahedral

and most low occupancy oxygen atoms were modelled with isotropic thermal parameters. Some electron density peaks and holes remain close to the disordered perrhenates, either indicating further minor disorder which could not be resolved or absorption effects. Restraints were also applied to the partial occupancy acetonitrile molecules. The hydrogen atoms of some water and acetonitrile molecules could not be located in the electron density map and were therefore not included in the model.

Further reflecting the solvent loss and poor diffraction properties there is a significant amount of void volume in the lattice containing smeared electron density from disordered solvent and the remaining anions. Consequently the SQUEEZE[11] function of PLATON[12] was employed to remove the contribution of the electron density associated with these remaining anions and further highly disordered solvent, which gave a potential solvent accessible void of 3718 Å^3 per unit cell (a total of approximately 1111 electrons). Diffuse solvent molecules could not be assigned to acetonitrile or diisopropyl ether and were therefore not included in the formula. Consequently, the molecular weight and density given above are underestimated.

CheckCIF gives six A and three B level alerts. The A level alerts all result from low occupancy perrhenate anions for which the oxygen atoms could not be modelled (apparent isolated metal atoms). The B level alerts result from the limited resolution, and the acetonitrile or water molecules for which the hydrogens were not modelled (apparent singly bonded carbon or isolated oxygen atom).

2·12NTf$_2$·2CH$_3$CN [+ solvent]

Formula $C_{280}H_{192}F_{72}N_{58}O_{48}S_{24}Zn_6$, *M* 7666.56, Triclinic, space group P-1 (#2), *a* 24.6082(8), *b* 25.2244(10), *c* 32.5068(14) Å, α 80.935(4), β 71.464(3), γ 69.421(3)°, *V* 17886.5(13) Å^3, D_c 1.423 g cm^{-3}, *Z* 2, crystal size 0.030 by 0.020 by 0.015 mm, colour red brown, habit block, temperature 100(2) Kelvin, λ(Synchrotron) 0.6889 Å, μ(Synchrotron) 0.589 mm^{-1}, T(Analytical)$_{min,max}$ 0.919224730858137, 1.0, $2\theta_{max}$ 33.36, *hkl* range -20 20, -21 21, -27 27, *N* 36480, N_{ind} 20613(R_{merge} 0.1531), N_{obs} 9809(I > 2σ(I)), N_{var} 3947, residuals* *R*1(*F*) 0.2216, *wR*2(F^2) 0.5291, GoF(all) 1.483, $\Delta\rho_{min,max}$ -0.553, 1.219 e$^-$ Å^{-3}.

*$R1 = \Sigma||F_o| - |F_c||/\Sigma|F_o|$ for $F_o > 2\sigma(F_o)$; $wR2 = (\Sigma w(F_o^2 - F_c^2)^2/\Sigma(wF_c^2)^2)^{1/2}$ all reflections

$w=1/[\sigma^2(F_o^2)+(0.2000P)^2]$ where $P=(F_o^2+2F_c^2)/3$

Specific refinement details:

The crystals of **2**·12NTf$_2$·2CH$_3$CN [+ solvent] were grown by diffusion of diethyl ether into an acetonitrile solution of **2**·12NTf$_2$. The crystals employed immediately lost solvent after removal from the mother liquor and rapid handling prior to flash cooling in liquid nitrogen was required to collect data. The crystals were very small and weakly diffracting and were subject to rapid beam damage during data collection using synchrotron radiation resulting in only 95% completeness. Consequently the quality of the

integration is also less than ideal. Few reflections at greater than 1.2 Å resolution were observed and data were trimmed accordingly. As a consequence of the poor diffraction properties of the sample, the values of the R1, wR and wR2 factors are larger than typical small molecule structures. Nevertheless, the quality of the data is far more than sufficient to establish the connectivity of the structure. The asymmetric unit was found to contain one complete $Zn_6L_3L'_2$ assembly as well as associated counterions and solvent molecules.

Due to the limited resolution, bond length and angle restraints were required in order to obtain a reasonable model for the organic parts of the structure. The GRADE program[13] was thus employed, using the GRADE Web Server,[14] to generate a full set of bond distance and angle restraints (DFIX, DANG, FLAT) for each type of organic ligand. Thermal parameter restraints (SIMU, RIGU, ISOR) were also applied to all atoms except for zinc to facilitate anisotropic refinement.

The anions within the structure also show evidence of disorder. Three triflimide anions were modelled as disordered over two locations and several others show evidence of further disorder which could not be modelled due to the limited resolution of the data. The occupancies of most located anions were freely refined. Substantial bond length and thermal parameter restraints were applied to facilitate stable refinement of the anions and the lower occupancy anions were modelled with isotropic thermal parameters.

Further reflecting the solvent loss and poor diffraction properties there is a significant amount of void volume in the lattice containing smeared electron density from disordered solvent and ca. 5.6 unresolved anions per assembly. No satisfactory model for these anions could be found despite numerous attempts at modelling. Consequently the SQUEEZE[11] function of PLATON[12] was employed to remove the contribution of the electron density associated with these anions and further highly disordered solvent, which gave a potential solvent accessible void of 5116 Å^3 per unit cell (a total of approximately 1727 electrons). Diffuse solvent molecules could not be assigned to acetonitrile or diethyl ether and were therefore not included in the formula. Consequently, the molecular weight and density given above are underestimated.

CheckCIF gives 4 level A alerts and 2 B level alerts. These alerts all result from the poor diffraction properties of the sample (low resolution, low completeness, low data to parameter ratio, high residuals, low bond precision) as described above.

3·12BF$_4$·6.5CH$_3$CN [+ solvent]

Formula $C_{283}H_{217.50}B_{12}F_{48}N_{50.50}Zn_6$, *M* 5759.50, Monoclinic, space group P 21/n (#14), *a* 28.1082(2), *b* 35.1636(2), *c* 30.5826(2) Å, β 92.8230(10), *V* 30190.7(3) Å^3, D_c 1.267 g cm^{-3}, *Z* 4, crystal size 0.060 by 0.040 by 0.030 mm, colour red brown, habit block, temperature 100(2) Kelvin, λ(Synchrotron) 0.6889 Å, μ(Synchrotron) 0.512 mm^{-1}, T(Analytical)$_{min,max}$ 0.8742621909246152, 1.0, $2\theta_{max}$ 40.30, *hkl* range -27 28, -35 35, -29 29, *N* 102481, N_{ind} 30468(R_{merge} 0.0558), N_{obs}

16905($I > 2\sigma(I)$), N_{var} 3839, residuals* $R1(F)$ 0.0803, $wR2(F^2)$ 0.2568, GoF(all) 0.966, $\Delta\rho_{min,max}$ -0.547, 1.157 $e^- Å^{-3}$.

*$R1 = \Sigma||F_o| - |F_c||/\Sigma|F_o|$ for $F_o > 2\sigma(F_o)$; $wR2 = (\Sigma w(F_o^2 - F_c^2)^2/\Sigma(wF_c^2)^2)^{1/2}$ all reflections

$w=1/[\sigma^2(F_o^2)+(0.1794P)^2]$ where $P=(F_o^2+2F_c^2)/3$

Specific refinement details:

The crystals of **3**·12BF$_4$·6.5CH$_3$CN [+ solvent] were grown by diffusion of diisopropyl ether into an acetonitrile solution of **3**·12NTf$_2$ containing excess ^{n}Bu$_4$N·BF$_4$. The crystals employed immediately lost solvent after removal from the mother liquor and rapid handling prior to flash cooling in liquid nitrogen was required to collect data. Despite these measures and the use of synchrotron radiation few reflections at greater than 1.0 Å resolution were observed and the data were trimmed accordingly. Nevertheless, the quality of the data is far more than sufficient to establish the connectivity of the structure. The asymmetric unit was found to contain one complete $Zn_6L_3L'_2$ assembly and associated counterions and solvent molecules.

Due to the limited resolution bond lengths and angles within pairs of chemically identical organic ligands were restrained to be similar to each other and thermal parameter restraints (SIMU, RIGU) were applied to all atoms except for zinc. Three phenyl rings within the tetratopic ligands were modelled as disordered over two locations and bond length and angle restraints were applied to achieve a reasonable model.

The anions within the structure show evidence of disorder. Four of the tetrafluoroborate anions were modelled as disordered over two locations and all anions were restrained to be approximately tetrahedral. The occupancies of all located anions were allowed to refine freely. Some additional minor occupancy positions of the anions could not be located in the electron density map and were not included in the model resulting in a discrepancy of ca. 1.3 anions per $Zn_6L_3L'_2$ assembly. Bond length restraints were also applied to some acetonitrile solvent molecules.

Further reflecting the solvent loss and poor diffraction properties there is a significant amount of void volume in the lattice containing smeared electron density from disordered solvent and the remaining anions. Consequently the SQUEEZE[11] function of PLATON[12] was employed to remove the contribution of the electron density associated with the remaining anions and further highly disordered solvent, which gave a potential solvent accessible void of 4762 $Å^3$ per unit cell (a total of approximately 1457 electrons). Diffuse solvent molecules could not be assigned to acetonitrile or diisopropyl ether and were therefore not included in the formula. Consequently, the molecular weight and density given above are underestimated.

CheckCIF gives one A and one B level alert. These alerts result from the limited resolution and consequent poor data to parameter ratio.

4·16PF$_6$·6.75CH$_3$CN·0.5iPr$_2$O [+ solvent]

Formula $C_{388.50}H_{299.25}F_{96}N_{66.75}O_{0.50}P_{16}Zn_8$, *M* 8753.16, Triclinic, space group P-1 (#2), *a* 23.7853(2), *b* 29.0325(3), *c* 39.4785(3) Å, α 75.3430(10), β 75.1210(10), γ 77.7410(10)°, *V* 25171.7(4) Å^3, D_c 1.155 g cm^{-3}, *Z* 2, crystal size 0.050 by 0.040 by 0.020 mm, colour red-brown, habit block, temperature 100(2) Kelvin, λ(Synchrotron) 0.6889 Å, μ(Synchrotron) 0.469 mm^{-1}, T(Analytical)$_{min,max}$ 0.8518939105929694, 1.0, $2\theta_{max}$ 40.30, *hkl* range -23 23, -29 29, -39 39, *N* 221289, N_{ind} 52629(R_{merge} 0.0596), N_{obs} 18035(I > 2σ(I)), N_{var} 5302, residuals* *R*1(*F*) 0.0830, *wR*2(F^2) 0.2509, GoF(all) 0.783, $\Delta\rho_{min,max}$ -0.294, 0.507 e$^-$ Å^{-3}.

*$R1 = \Sigma||F_o| - |F_c||/\Sigma|F_o|$ for $F_o > 2\sigma(F_o)$; $wR2 = (\Sigma w(F_o^2 - F_c^2)^2/\Sigma(wF_c^2)^2)^{1/2}$ all reflections

$w=1/[\sigma^2(F_o^2)+(0.1612P)^2]$ where $P=(F_o^2+2F_c^2)/3$

Specific refinement details:

The crystals of **4**·16PF$_6$·6.75CH$_3$CN·0.5iPr$_2$O [+ solvent] were grown by diffusion of diisopropyl ether into an acetonitrile solution of **4**·16NTf$_2$ containing excess ^{n}Bu$_4$N·PF$_6$ and anthraquinone. The crystals employed immediately lost solvent after removal from the mother liquor and rapid handling prior to flash cooling in liquid nitrogen was required to collect data. Despite these measures and the use of synchrotron radiation few reflections at greater than 1.0 Å resolution were observed and the data were trimmed accordingly. Furthermore there was a significant drop-off in diffraction intensity after around 1.25 Å resolution resulting in a low ratio of observed/unique reflections. Nevertheless, the quality of the data is far more than sufficient to establish the connectivity of the structure. The asymmetric unit was found to contain one complete Zn$_8$L$_4$L′$_2$ assembly and associated counterions and solvent molecules.

Due to the limited resolution bond lengths and angles within pairs of chemically identical organic ligands were restrained to be similar to each other and thermal parameter restraints (SIMU, RIGU) were applied to all atoms except for zinc. Additional DFIX and DANG restraints were applied to a pyridyl ring displaying a high degree of thermal motion.

The anions within the structure show evidence of disorder. Two of the hexafluorophosphate anions were modelled as disordered over two locations and bond length and thermal parameter restraints were required to obtain a reasonable model for most of the anions. The occupancies of all located anions were allowed to refine freely. Despite the use of thermal parameter restraints several of the anions show large average Ueq values as a consequence of high levels of thermal motion. Attempts to model these anions as disordered over multiple discrete locations were not successful. Some additional minor occupancy positions of the anions could not be located in the electron density map and were not included in the model resulting in a discrepancy of ca. 2.7 anions per Zn$_8$L$_4$L′$_2$ assembly. Bond length

restraints were also applied to the encapsulated diisopropyl ether molecule and some acetonitrile solvent molecules.

Further reflecting the solvent loss and poor diffraction properties there is a significant amount of void volume in the lattice containing smeared electron density from disordered solvent and the remaining anions. Consequently the SQUEEZE[11] function of PLATON[12] was employed to remove the contribution of the electron density associated with the remaining anions and further highly disordered solvent, which gave a potential solvent accessible void of 7668 Å^3 per unit cell (a total of approximately 2117 electrons). Diffuse solvent molecules could not be assigned to acetonitrile or diisopropyl ether and were therefore not included in the formula. Consequently, the molecular weight and density given above are underestimated.

CheckCIF gives one A and six B level alerts. These alerts result from the limited resolution of the data and thermal motion of the hexafluorophosphate anions (high Ueq values) as described above.

5 Volume calculations

The volume of the interior cavity of each of metal-organic prisms **1**–**4** was calculated from the corresponding crystal structure using MoloVol.[15] The "probe-occupied volume" (V_{occ}) was calculated in "single-probe mode", using the following parameters:

Small probe radius: 1.2 Å for **1** and **4**, 1.3 Å for **2** and 1.5 Å for **3**.

Grid resolution: 0.1 Å

Optimisation depth: 4 (default value)

Element radii. Zn : 2.39 Å, N : 1.66 Å, H : 1.20 Å, C : 1.77 Å

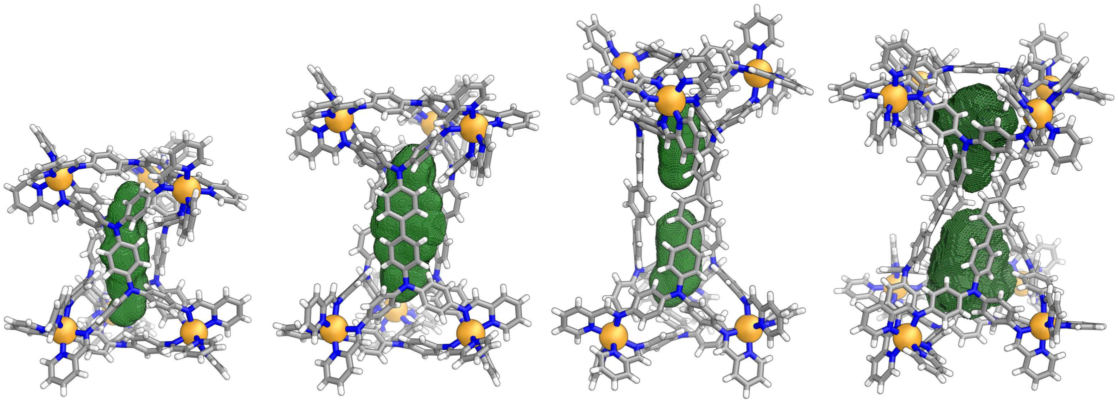

140 Å^3 | 210 Å^3 | 86 Å^3 + 72 Å^3 | 246 Å^3 + 168 Å^3

1 | **2** | **3** | **4**

Figure S73. Void space (green mesh) within the crystal structures of **1**–**4** calculated using MoloVol.[15]

6 References

(1) Rosenau, C. P.; Jelier, B. J.; Gossert, A. D.; Togni, A. Exposing the Origins of Irreproducibility in Fluorine NMR Spectroscopy. *Angew. Chem. Int. Ed.* **2018**, *57*, 9528-9533.

(2) Wu, D. H.; Chen, A.; Johnson, C. S. An Improved Diffusion-Ordered Spectroscopy Experiment Incorporating Bipolar-Gradient Pulses. *J. Magn. Reson., Ser. A* **1995**, *115*, 260-264.

(3) Davies, J. A.; Tarzia, A.; Ronson, T. K.; Auras, F.; Jelfs, K. E.; Nitschke, J. R. Tetramine Aspect Ratio and Flexibility Determine Framework Symmetry for Zn_8L_6 Self-Assembled Structures. *Angew. Chem. Int. Ed.* **2023**, *62*, e202217987.

(4) Allan, D. R.; Nowell, H.; Barnett, S. A.; Warren, M. R.; Wilcox, A.; Christensen, J.; Saunders, L. K.; Peach, A.; Hooper, M. T.; Zaja, L.; Patel, S.; Cahill, L.; Marshall, R.; Trimnell, S.; Foster, A. J.; Bates, T.; Lay, S.; Williams, M. A.; Hathaway, P. V.; Winter, G.; Gerstel, M.; Wooley, R. W. A Novel Dual Air-Bearing Fixed-χ Diffractometer for Small-Molecule Single-Crystal X-ray Diffraction on Beamline I19 at Diamond Light Source. *Crystals* **2017**, *7*, 336.

(5) (a) Evans, P. Scaling and Assessment of Data Quality. *Acta Cryst.* **2006**, *D62*, 72-82. (b) Winter, G. xia2: An Expert System for Macromolecular Crystallography Data Reduction. *J. Appl. Crystallogr.* **2010**, *43*, 186-190. (c) Winter, G.; Waterman, D. G.; Parkhurst, J. M.; Brewster, A. S.; Gildea, R. J.; Gerstel, M.; Fuentes-Montero, L.; Vollmar, M.; Michels-Clark, T.; Young, I. D.; Sauter, N. K.; Evans, G. *DIALS*: Implementation and Evaluation of a New Integration Package. *Acta Cryst.* **2018**, *D74*, 85-97.

(6) Farrugia, L. J. WinGX and ORTEP for Windows: An Update. *J. Appl. Crystallogr.* **2012**, *45*, 849-854.

(7) Evans, P. R.; Murshudov, G. N. How Good Are My Data and What is the Resolution? *Acta Cryst.* **2013**, *D69*, 1204-1214.

(8) Winn, M. D.; Ballard, C. C.; Cowtan, K. D.; Dodson, E. J.; Emsley, P.; Evans, P. R.; Keegan, R. M.; Krissinel, E. B.; Leslie, A. G. W.; McCoy, A.; McNicholas, S. J.; Murshudov, G. N.; Pannu, N. S.; Potterton, E. A.; Powell, H. R.; Read, R. J.; Vagin, A.; Wilson, K. S. Overview of the *CCP4* Suite and Current Developments. *Acta Cryst.* **2011**, *D67*, 235-242.

(9) Sheldrick, G. M. *SHELXT* - Integrated Space-Group and Crystal-Structure Determination. *Acta. Cryst.* **2015**, *A71*, 3-8.

(10) Sheldrick, G. M. Crystal Structure Refinement with *SHELXL*. *Acta. Cryst.* **2015**, *C71*, 3-8.

(11) van der Sluis, P.; Spek, A. L. BYPASS: An Effective Method for the Refinement of Crystal Structures Containing Disordered Solvent Regions. *Acta Cryst.* **1990**, *A46*, 194-201.

(12) Spek, A. L., *PLATON: A Multipurpose Crystallographic Tool*. Utrecht University: Utrecht, The Netherlands, 2008.

(13) Smart, O. S.; Sharff, A.; Holstein, J.; Womack, T. O.; Flensburg, C.; Keller, P.; Paciorek, W.; Vonrhein, C.; Bricogne, G. *Grade2 version 1.3.0*. Global Phasing Ltd.: Cambridge, United Kingdom, 2021.

(14) Smart, O. S.; Womack, T. O., *Grade Web Server*. Global Phasing Ltd.: 2014.

(15) Maglic, J. B.; Lavendomme, R. MoloVol: An Easy-to-Use Program for Analyzing Cavities, Volumes and Surface Areas of Chemical Structures. *J. Appl. Crystallogr.* **2022**, *55*, 1033–1044.
